# Isoguanine and 5-Methyl-Isocytosine Bases, In Vitro and In Vivo

**DOI:** 10.1002/chem.201406392

**Published:** 2015-02-13

**Authors:** Omprakash Bande, Rania Abu El Asrar, Darren Braddick, Shrinivas Dumbre, Valérie Pezo, Guy Schepers, Vitor B Pinheiro, Eveline Lescrinier, Philipp Holliger, Philippe Marlière, Piet Herdewijn

**Affiliations:** [a] Medicinal Chemistry, Rega Institute for Medical Research KU Leuven, Minderbroedersstraat 10, 3000 Leuven (Belgium) E-mail : Piet.Herdewijn@rega.kuleuven.be; [b] ISSB, Génopole genavenir 6, Equipe Xénome 5 rue Henri Desbruères 91030 Evry Cedex (France); [c] CEA, DSV, IG, Genoscopeya2 rue Gaston Crémieux 91057 Evry Cedex (France); [d] Institute of Structural and Molecular Biology, University College, University of London Darwin Building, Gower Street, London, WC1E 6BT (UK); [e] MRC Laboratory of Molecular Biology, Francis Crick Avenue, Cambridge Biomedical Campus Cambridge CB2 0QH (UK)

**Keywords:** HNA, isoG, polymerase, nucleosides, XNA plasmid

## Abstract

The synthesis, base-pairing properties and in vitro and in vivo characteristics of 5-methyl-isocytosine (isoC^Me^) and isoguanine (isoG) nucleosides, incorporated in an HNA(h) (hexitol nucleic acid)–DNA(d) mosaic backbone, are described. The required h-isoG phosphoramidite was prepared by a selective deamination as a key step. As demonstrated by *T*_m_ measurements the hexitol sugar showed slightly better mismatch discrimination against dT. The d-isoG base mispairing follows the order T>G>C while the h-isoG base mispairing follows the order G>C>T. The h- and d-isoC^Me^ bases mainly mispair with G. Enzymatic incorporation experiments show that the hexitol backbone has a variable effect on selectivity. In the enzymatic assays, isoG misincorporates mainly with T, and isoC^Me^ misincorporates mainly with A. Further analysis in vivo confirmed the patterns of base-pair interpretation for the deoxyribose and hexitol isoC^Me^/isoG bases in a cellular context, through incorporation of the bases into plasmidic DNA. Results in vivo demonstrated that mispairing and misincorporation was dependent on the backbone scaffold of the base, which indicates rational advances towards orthogonality.

## Introduction

Encoding heritable information in polymers other than DNA and RNA in vivo will result in the creation of XNA (Xeno nucleic acid) modified organisms (XMO’s) unable to communicate this information with the environment.[1] Several artificial nucleic acids have been considered as XNA,[2] and few of them have been evaluated in vivo.[3] Transliteration of XNA to DNA in vivo has been described for base-modified XNA,[3b] for sugar-modified XNA[3a,c] and for XNA’s in which both sugar and base are modified.[3c,d] The substitution of the thymine base by chlorouracil in a bacterial genome[4] demonstrates the feasibility of supporting life with a genome consisting of a non-natural base.

A recent breakthrough in the field of XNA has been reported by Malyshev et al.,[3e] which shows recognition of a synthetic base pair in vivo, and by Pinheiro et al.,[5] which shows that up to six artificial polymers can be used to propagate genetic information in vitro. Whatever the design may be, an ideal orthogonal genomic XNA should preferentially contain a modified backbone as well as modified base pairs.

**Figure 1:**
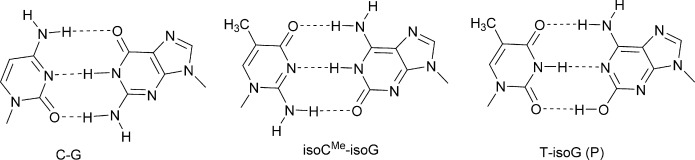
Putative base-pair motifs of duplexes with antiparallel strand orientation.

**Figure 2:**
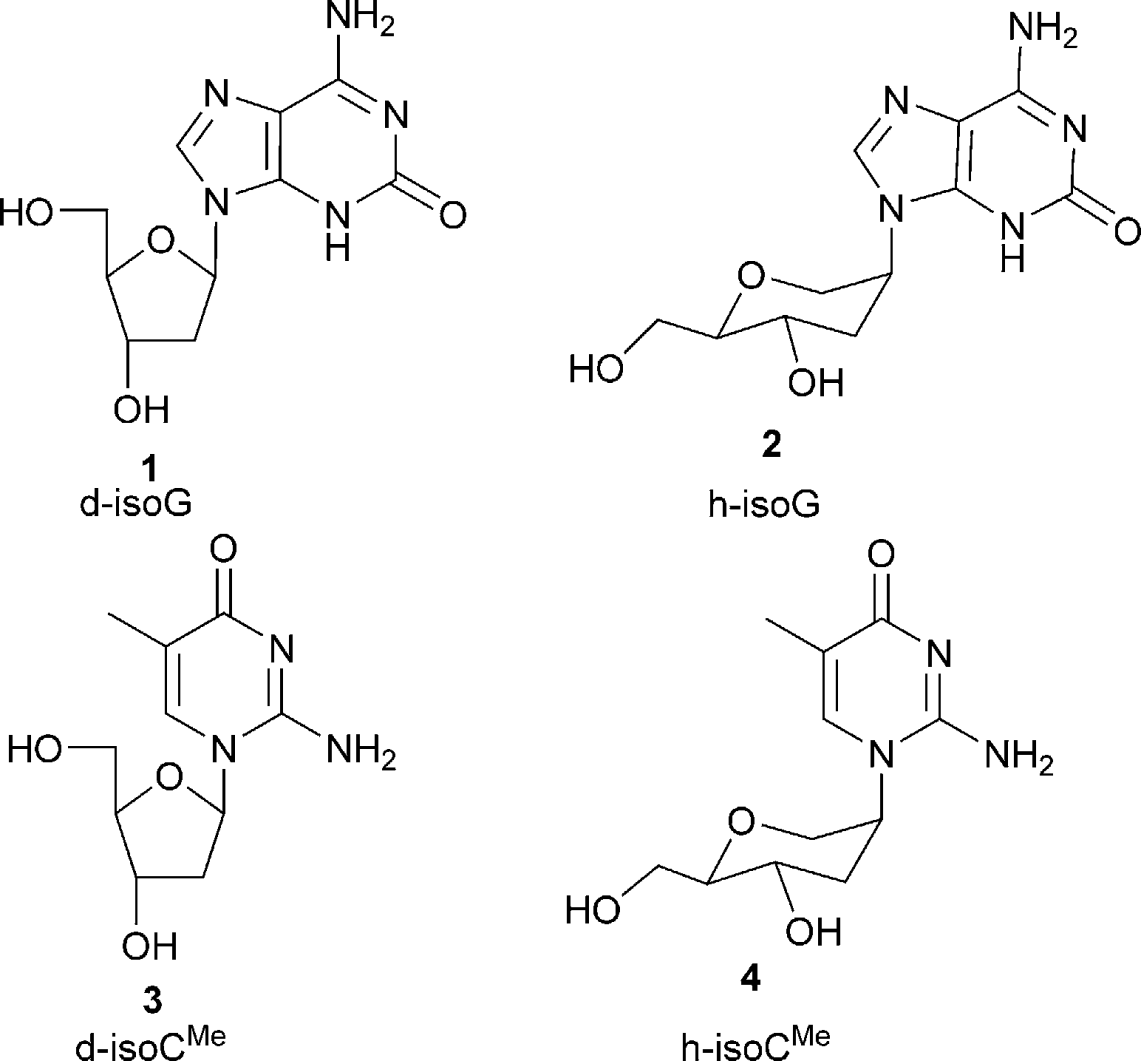
Deoxyribose and hexitol isoG and isoC^Me^ nucleosides.

The development of an alternative (third) base pair has been primarily pursued for the expansion of the genetic alphabet of DNA. The isoguanine[6] (isoG):isocytosine[7] (isoC) nonstandard base pair (Figure 1) was first proposed by A. Rich,[8] and it forms three H-bonds similar to the guanine (G):cytosine (C) base pair. Due to the low stability of isoC under alkaline conditions, the 5-methyl derivative (isoC^Me^) was used instead.[9] This unnatural isoG:isoC^Me^ base pair was studied by S. Benner,[10] and evaluated in PCR amplification showing 96 % fidelity.[11] Results from these studies revealed that the pairing selectivity in DNA was not adequate and restricted its applications. During studies on the incorporation of isoG opposite to isoC^Me^ in DNA by polymerase misincorporation of thymidine (T) and/or uridine (U) opposite to the minor enol tautomer[12] of isoG was observed. Progress was achieved using 7-deaza-isoG,[13] halogenated 7-deaza-isoG,[14] and 8-aza-isoG[15] as analogues of isoG, showing a shift towards the keto form in the tautomeric keto–enol equilibrium. However, the error rate per cycle for the amplification of isoC^Me^:isoG systems was still too high, which may result in unidirectional loss of information during amplification[16] (i.e. revert to an all-natural genetic system). Changing the chemistry of the nucleic acid backbone may have an influence on base pairing. In addition, studies regarding enzymatic incorporation of sugar-modified isoC^Me^ and isoG have not been reported. Hexitol nucleic acid (HNA)[17] is one example of a modified backbone that can be used to study how sugar modification influences duplex stability, the encoding of information, and its passage through heredity.[3a,c] HNA has an additional methylene group between C1′ and O4′ of the β-d-2-deoxyribose moiety and forms a very stable self-complementary duplex and stable duplexes with their natural counterparts DNA and RNA.[17a] The order of duplex stability is: HNA–HNA>HNA–RNA>HNA–DNA.[17a] HNA forms A-type duplexes. An HNA-dependent DNA polymerase and a DNA-dependent HNA polymerase are available for biological applications.[5] Here, as part of our continuing research on the selection of base- and sugar-modified nucleic acids as orthogonal information systems,[3c,d, 18] we investigated the isoG:isoC^Me^ base-pairing system in an HNA context. Therefore, the compounds depicted in Figure 2 were synthesised and investigated for their base-pairing properties. The compounds were analysed experimentally, utilising in vitro hybridization and DNA polymerase incorporation assays as well as an in vivo HNA-dependent DNA synthesis assay.

## Results and Discussion

**Scheme 1:**
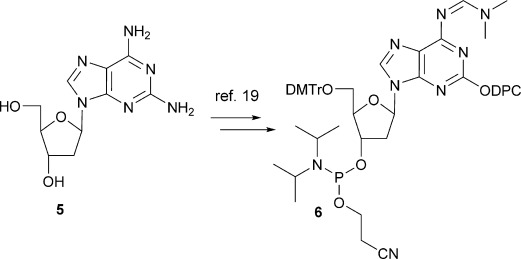
Synthesis of phosphoramidite of protected deoxyribose-isoG.

The phosphoramidite building blocks of d-isoG **6** was synthesised from **5** by following a reported procedure (Scheme 1).[19]

**Scheme 2:**
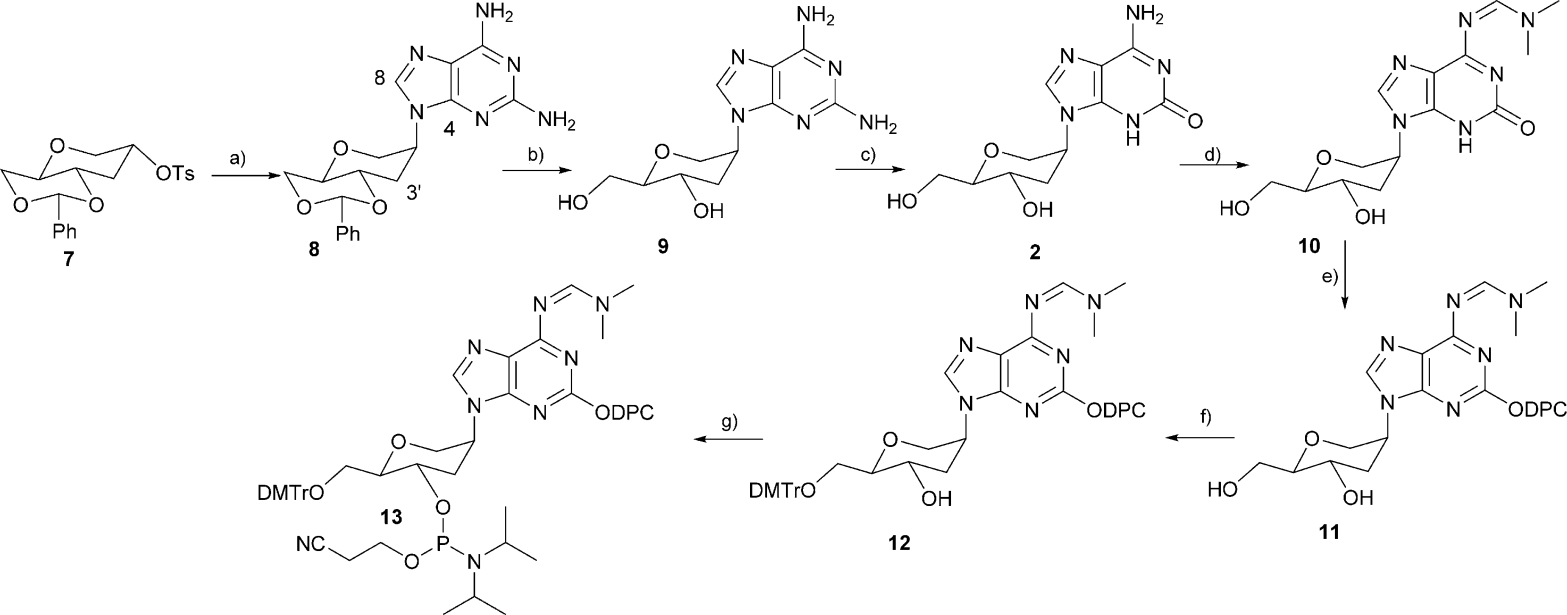
Synthesis of phosphoramidite of protected hexitol-isoG. a) 2,6-Diaminopurine, NaH, DMF, 90 °C, 12 h, 79 %; b) CH_3_COOH/H_2_O 6:4, 60 °C, 4 h, 91 %; c) NaNO_2_, CH_3_COOH, H_2_O, 55 °C, 10 min, 76 %; d) Me_2_NCH(OMe)_2_, CH_3_OH, RT, 12 h, 80 %; e) Ph_2_NCOCl, *i*Pr_2_NEt, pyridine, RT, 1 h, 76 %, f) (MeO)_2_TrCl, pyridine, 0 °C to RT, 12 h, 73 %, g) (*i*Pr_2_N)_2_POC_2_H_4_CN, 1*H*-tetrazole, DCM, 0 °C to RT, 1 h, 75 %.

As depicted in Scheme 2, the synthesis of h-isoG started from the 2-*O*-tosylate of 1,5-anhydro-4,6-*O*-benzylidene-d-glucitol (**7**), which was prepared as previously reported by us.[20] The tosylated sugar **7** was treated with the sodium salt of 2-aminoadenine in DMF at 90 °C for 12 h yielding 79 % of 2,6-diaminopurine hexitol nucleoside **8**. HMBC of 2D NMR was used to illustrate the correct connection of the nucleobase to the sugar moiety. For compound **8**, the HMBC spectrum showed that the proton of C-3′ of the sugar moiety was coupled with the C-4 and C-8 of the 2-aminoadenine moiety, thus confirming that the C-3′ of the sugar moiety is linked to the *N*-9 of the adenine moiety via a C–N bond. Moreover, the spectral and analytical data of **8** were found to be in agreement with a previous report.[21] The cleavage of the benzylidene–acetal protecting group was carried out using acetic acid and water at 60 °C for 4 h, to obtain compound **9** in 91 % yield.

In the next step, the 2-aminoadenosine hexitol **9** was deaminated selectively by diazotisation of the 2-NH_2_ group using sodium nitrate in acetic acid and water at 55 °C for 10 min, affording h-isoG **2** in 76 % yield. The free amino group of compound **2** was protected with an *N*,*N*-dimethylamino methylidene residue by using dimethylformamide dimethyl acetal in methanol at room temperature for 12 h. As has been described for the isoguanosine derivatives, the protection of the 2-oxo group with a diphenylcarbamoyl residue resulted in significantly higher coupling yields during oligonucleotide synthesis.[22] Hence, **10** was treated with diphenylcarbamoyl chloride in pyridine at room temperature for one hour to furnish the 2-oxo-protected nucleoside **11** in 76 % yield. Subsequently, compound **11** was tritylated using dimethoxytrityl chloride (DMTrCl) in pyridine with 4-dimethylaminopyridine (DMAP) as a catalyst. Phosphitylation of the DMTr derivative **12** was performed in anhydrous dichloromethane in the presence of 1*H*-tetrazole and 2-cyanoethyl *N*,*N*,*N*′,*N*′-tetraisopropylphosphorodiamidite, furnishing the phosphoramidite **13**.

**Scheme 3:**
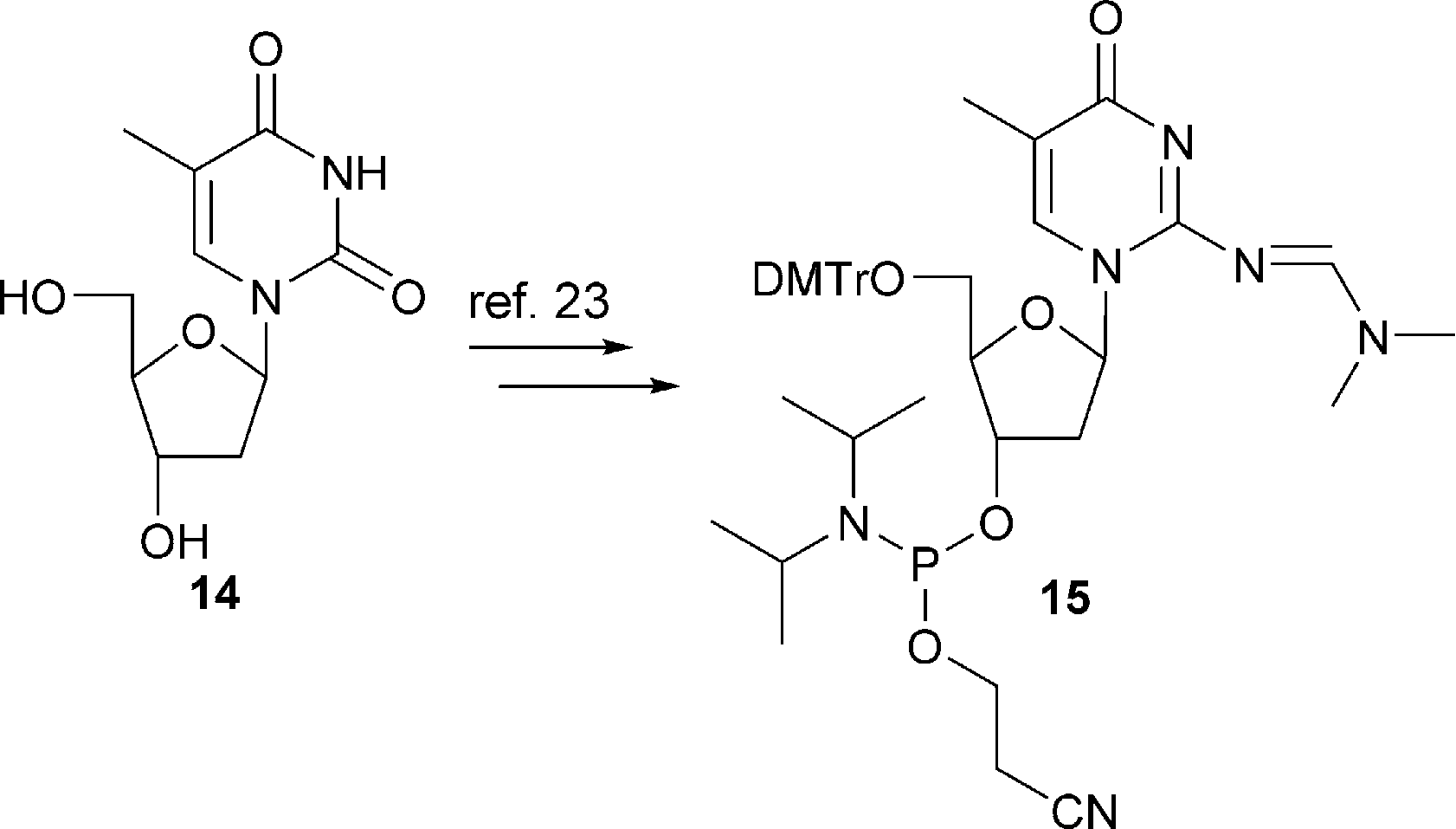
Synthesis of phosphoramidite of protected deoxyribose-isoC^Me^.

The phosphoramidite of d-isoC^Me^ **15** was accessed by following a procedure reported by Seela[23] (Scheme 3), starting from thymidine **14**. For the synthesis of the h-isoC^Me^ **25**, the required 1′,5′-anhydro-4′,6′-*O*-benzylidene-3′-*O*-methanesulfonyl-2′-deoxy-2′-(5-methyl-isocytosin-1-yl)-d-altro-hexitol (**17**) was prepared from commercially available 1,5:2,3-dianhydro-4,6-*O*-benzylidene-d-allitol (**16**) by using an existing procedure.[24] Compound **17** was treated with saturated ammonia (methanol) in a sealed tube at 160 °C for 60 h affording the benzylidene-protected h-isoC^Me^ **19** in 70 % yield. This one-pot two-step reaction implies the in situ generation of a 2,3′-anhydro nucleoside **18** that concomitantly undergoes nucleophilic opening with ammonia at the C2 of the pyrimidinone ring skeleton with an inversion of configuration at the C3′-position. The protection of the C2 amino group of compound **19** was carried out using dimethylformamide dimethyl acetal in methanol at 65 °C for 3 h affording compound **20** in 80 % yield. For the deoxygenation of the C3′-hydroxy group, the Barton–McCombie reaction was used. We first converted the hexitol nucleoside **20** into the corresponding thiocarbamate **21** (88 % yield), which, after radical reduction in the presence of tributylstannane and azobisisobutyronitrile (AIBN), gave the desired N-protected isoC^Me^ nucleoside **22** in 69 % yield.

**Scheme 5:**
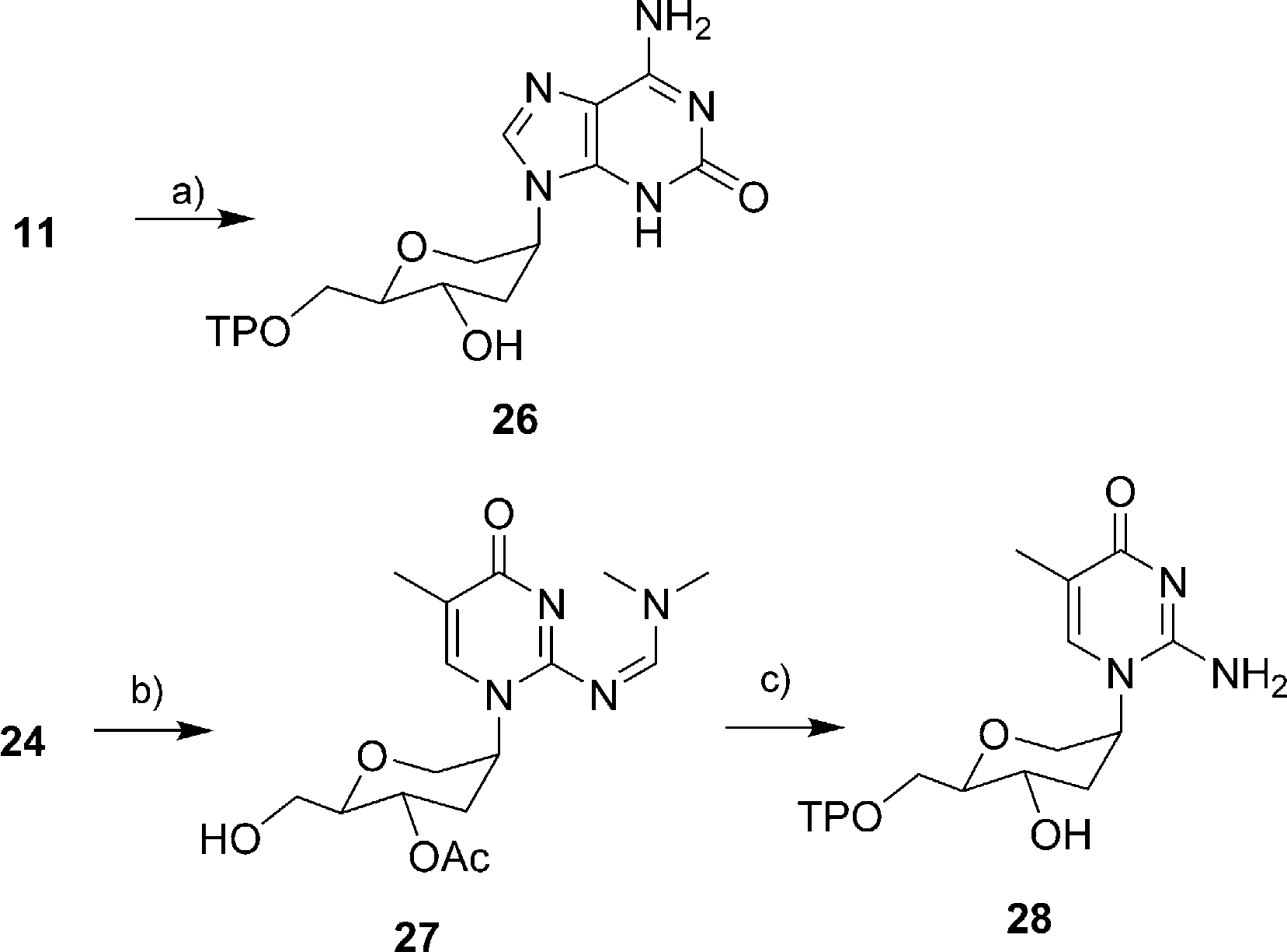
Synthesis of phosphoramidite of protected hexitol-isoC^Me^. a) sat. NH_3_ in CH_3_OH, 160 °C, 60 h, 70 %; b) Me_2_NCH(OMe)_2_, CH_3_OH, 65 °C, 3 h, 88 %; c) Im_2_S, DMF, 12 h, 88 %; d) AIBN, *n*Bu_3_SnH, toluene, 80 °C, 3 h, 69 %; e) CH_3_COOH/H_2_O (6:4), 60 °C, 4 h, 93 %; f) (MeO)_2_TrCl, pyridine, 0 °C to RT, 12 h, 88 %; g) (*i*Pr_2_N)_2_POC_2_H_4_CN, 1*H*-tetrazole, dichloromethane, 0 °C to RT, 1 h, 81 %.

Removal of benzylidene acetal protective group led to compound **23** in 93 % yield. Subsequently, compound **23** was tritylated to **24** and finally phosphitylation was performed to obtain the phosphoramidite **25** (Scheme 5).

### Triphosphate synthesis

**Scheme 4:**
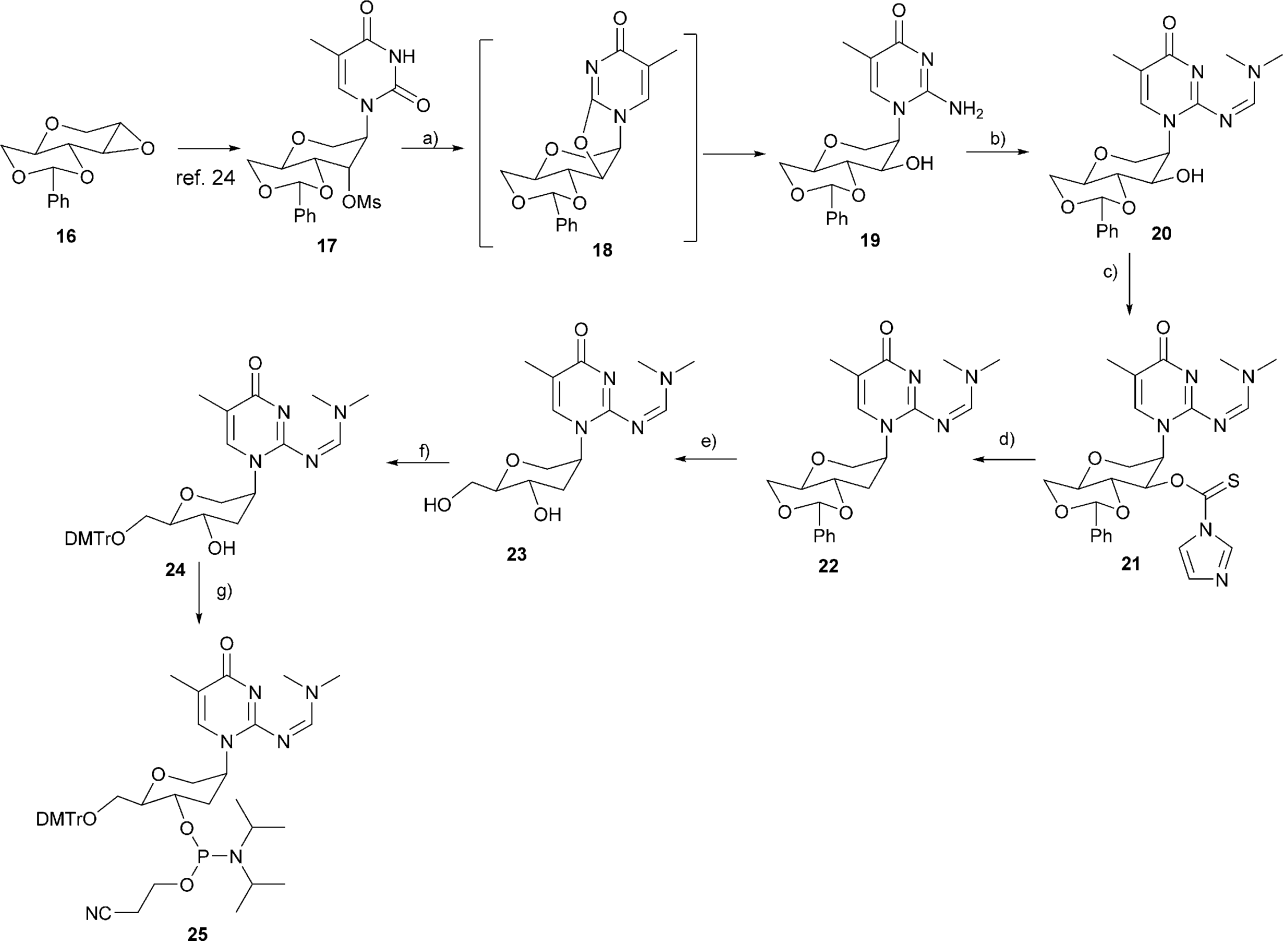
Synthesis of the triphosphates 26 and 28. a) i) TMP, POCl_3_, 0 °C, 5 h; ii) Bu_3_N, (NBu_4_)_3_HP_2_O_5_, 30 min; iii) 25 % NH_3_, 2 h; b) i) Ac_2_O, *N*,*N*-diisopropylethylamine, DMAP, THF, RT, 20 min; ii) dichloroacetic acid, dichloromethane, 0 °C to RT, 30 min; c) i) salicyl phosphorochloridite, pyridine/dioxane, 1 min, ii) (NBu_3_)_2_H_2_P_2_O_7_, NBu_3_, DMF, 30 min; iii) iodine, pyridine/water, 10 min; iv) 25 % NH_3_, 2 h.

The triphosphate building blocks of deoxyribose-isoG (d-isoGTP) and deoxyribose-isoC^Me^ (d-isoC^Me^TP) were synthesised following a reported protocol.[25] For the synthesis of triphosphate hexitol-isoG (h-isoGTP), the protected h-isoG **11** was converted to h-isoGTP **26** in a one-pot reaction by the Ludwig method.[26] In this procedure, regioselective phosphorylation of the 6′-hydroxy group of the sugar moiety was carried out with phosphoryl oxychloride in trimethyl phosphate followed by the addition of tetrabutylammonium pyrophosphate. The reaction product was deprotected with aqueous ammonia, isolated by ion-exchange chromatography and finally purified by RP-HPLC. As depicted in Scheme 4, the synthesis of h-isoC^Me^TP **28** started from DMTr-protected **24**, which was further converted to its 4′-acetate and removal of DMTr protecting group afforded compound **27** in 59 % over two steps. Compound **27** was converted to h-isoC^Me^TP **28** by phosphorylation with salicyl phosphorochloridite followed by addition of tributylammonium pyrophosphate and final oxidation of intermediate with iodine.[27]

The reaction product was deprotected with aqueous ammonia and purified by ion-exchange chromatography and RP-HPLC.

The oligonucleotides were prepared by using the common phosphoramidite method on a solid support employing a DNA synthesiser. The base-pairing properties of d-isoC^Me^ and h-isoC^Me^ with d-isoG and h-isoG were examined by hybridising oligomers with their complementary strands and determining the *T*_m_ of the hybrids by temperature-dependent UV spectroscopy. To investigate the base-pairing properties, the d-isoG nucleoside **1** and the h-isoG nucleoside **2** were incorporated in the antiparallel duplex 5′-(GGT AGC AG*C GGT G) replacing dG residues by **1** and **2**. The d-isoC^Me^ **3** and h-isoC^Me^ **4** nucleosides were incorporated in the sequence 3′-(CCA TCG TC*G CCA C) replacing dC residues by **3** and **4**. The strength of hybridization was studied by thermal denaturation experiments, which were determined at 260 nm in NaCl (0.1 m) buffer with KH_2_PO_4_ (20 mm, pH 7.5) and EDTA (0.1 mm) at a concentration of 4 μm for each strand. Hexitol nucleosides (h-isoG **2** and h-isoC^Me^ **4**) were incorporated opposite a deoxyribo-nucleoside (d-isoC^Me^ **3** and d-isoG **1**). Likewise, h-isoG:h-isoC^Me^ was evaluated as base pair. The stability of the duplexes was compared to the stability of the natural DNA duplex containing C:G (Table 1, entry 1) and isoC^Me^:isoG (entry 2) base pairs.

**Table 1:** *T*_m_ values of non-self-complementary antiparallel-stranded oligonucleotide duplexes containing 1 (d-isoG) and 2 (h-isoG) hybridised against 3 (d-isoC^Me^) and 4 (h-isoC^Me^)

Entry	Duplex	*T*_m_ [°C]
1	5′-GGT AGC AGC GGT G-3′ 3′-CCA TCG TCG CCA C-5′	61.3
2	5′-GGT AGC A**1**C GGT G-3′ 3′-CCA TCG T**3**G CCA C-5′	63.6
3	5′-GGT AGC A**1**C GGT G-3′ 3′-CCA TCG T**4**G CCA C-5′	62.1
4	5′-GGT AGC A**2**C GGT G-3′ 3′-CCA TCG T**3**G CCA C-5′	60.2
5	5′-GGT AGC A**2**C GGT G-3′ 3′-CCA TCG T**4**G CCA C-5′	60.0

The d-isoC^Me^:d-isoG pair gives a somewhat more stable duplex than the C:G pair, which may be due to the presence of a 5-methyl group on the isoC nucleoside. Incorporation of an h-isoG versus a d-isoC^Me^ nucleoside (Table 1, entry 4) gives a larger decrease in *T*_m_ (−3.4 °C) than incorporation of an h-isoC^Me^ versus a d-isoG nucleoside (entry 3) (−1.5 °C). The *T_m_* further decreases (−3.6 °C) when both strands contain an hexitol nucleoside (entry 5), which could be due to the fact that only one single hexitol base pair is introduced into a full DNA duplex.[17b] The experiment demonstrates that a stable isoC^Me^:isoG base pair can be formed within the context of a DNA–HNA mosaic duplex. To investigate the selectivity of base pairing of h-isoG and h-isoC^Me^, the four canonical nucleosides (dA, dT, dC, and dG) were placed opposite to the modification site, and *T*_m_ values were measured (Table 2). This study is done towards the function of the potential use of h-isoG and h-isoC^Me^ in vivo. We wondered what the influence would be on duplex stability when a natural nucleoside is incorporated opposite a hexitol nucleoside with an isoG or isoC^Me^ base. The base-pair stability for h-isoG decreases in the order d-isoC^Me^≈h-isoC^Me^>G≥C>T>A, whereas for d-isoG it is d-isoC^Me^>h-isoC^Me^>T>G>C≫A. This means that d-isoG forms the most stable mismatch with T and then with G, whereas h-isoG forms the most stable mismatch with G and C and then with T. The mismatch discrimination (Δ*T*_m_) is the lowest for h-isoG and the highest for the isoC^Me^ nucleosides. As with d-isoC^Me^, h-isoC^Me^ forms more stable mismatches with G than with the other natural bases. An interesting observation is that the h-isoG:h-isoC^Me^ base pair is 8.8 °C more stable than the h-isoG:dT base pair, whereas the d-isoG:d-isoC^Me^ base pair is 7.8 °C more stable than the d-isoG:dT base pair.

**Table 2:** *T*_m_ Values of non-self-complementary antiparallel-stranded oligonucleotide duplexes containing 1, 2, [5′-GGT AGC A (1 or 2) C GGT G-3′]; 3 and 4 [3′-CCA TCG T (3 or 4) G CCA C-5′] hybridised against complementary oligonucleotides of dA, dT, dC and dG

	A	T	C	G	Δ*T*_m_
d-isoG (**1**)	46.3	55.8	52.9	54.8	9.5
h-isoG (**2**)	48.3	51.2	53.4	53.7	5.4
d-isoC^Me^(**3**)	51.9	43.9	48.4	56.8	12.9
h-isoC^Me^(**4**)	47.7	41.1	46.1	54.8	13.7

### Incorporation experiments

We investigated the role of a hexitol scaffold in affecting the recognition and the selectivity of isoC^Me^:isoG base pairing during polymerase-catalysed oligonucleotide synthesis. To develop a successful orthogonal genetic system, mutual recognition of each base within a pair is imperative. Thus, eight primed (P1) templates (T2, T3, T5, T6, T8, T9, T11 and T12) were used (Table 3) and we tested the incorporation[28] of isoG triphosphates opposite isoC^Me^ nucleotides in the template and its reverse.

**Table 3:** Sequence of the primer and templates used in the DNA polymerase incorporation experiments

	Sequence (5′ 3′)
**P1**	CAG GAA ACA GCT ATG AC
	Sequence (5′ 3′)
**T1**	GTC CTT T GTC GAT ACT G**CT AAA**
**T4**	GTC CTT T GTC GAT ACT G **CCC TAAA**
**T7**	GTC CTT T GTC GAT ACT G **GCT GAA**
**T10**	GTC CTT T GTC GAT ACT G **GGG CTG AA**
**T2, T3, T8, T9**	GTC CTT T GTC GAT ACT G X* GT CAA
(X*=d-isoC^Me^, h-isoC^Me^, d-isoG, h-isoG for T2, T3, T8, T9, respectively)	
**T5, T6, T11, T12**	GTC CTT T GTC GAT ACT G X*X*X* GT CAA
(X*=d-isoC^Me^, h-isoC^Me^, d-isoG, h-isoG for T5, T6, T11, T12, respectively)	

**Figure 3:**
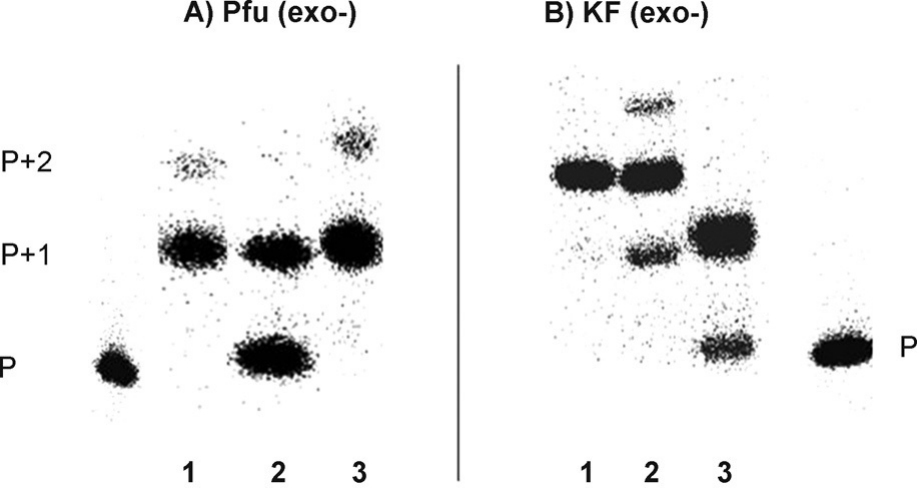
Phosphorimage of the enzymatic incorporation of 100 μm of 2) d-isoGTP into hybrid P1:T2 (1 d-isoC^Me^) and 3) h-isoGTP into hybrid P1:T3 (1 h-isoC^Me^) using 0.08 U μL^−1^ of Pfu (*exo*-) and KF (*exo*-). 0.08 U μL^−1^ of thermostable inorganic pyrophosphatase (TIPP) was included. 1 represents the incorporation of 10 μm dGTP into hybrid P1:T1 (dC) (positive control). Reaction time is 60 min.

**Figure 4:**
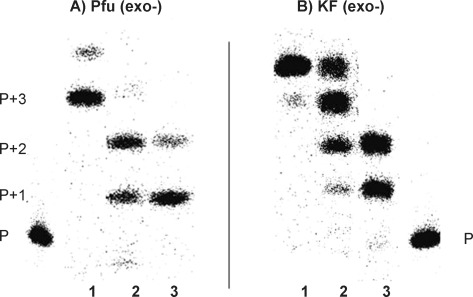
Phosphorimage of the enzymatic incorporation of 500 μm of 2) d-isoGTP into hybrid P1:T5 (3 d-isoC^Me^) and 3) h-isoGTP into hybrid P1:T6 (3 h-isoC^Me^) using 0.08 U μL^−1^ of Pfu (*exo*-) and KF (*exo*-). 0.08 U μL^−1^ of TIPP was included. 1 represents the incorporation of 20 μm dGTP into hybrid P1:T4 (dC) (positive control). Reaction time is 60 min.

Initially, we screened a series of mesophilic and thermostable polymerases for the incorporation of d-isoGMP (using d-isoGTP as reagent) and h-isoGMP (using h-isoGTP as reagent) opposite d-isoC^Me^ and h-isoC^Me^ nucleotides, respectively, in the template. Several of the polymerases tested that is, Taq, Tfi (*exo*-), T7 sequenase and Pol III α-subunit DNA polymerases failed to recognise the base-pairing of isoG:isoC^Me^ using the hexitol sugar moiety (data not shown). However, Pfu (*exo*-), Vent (*exo*-), Klenow fragment (KF) (*exo*-) and T4 (*exo*-) polymerases accepted both deoxyribose and hexitol isoGTP opposite their respective counterparts, albeit with differing degrees. For example, better incorporation was seen with Pfu (*exo*-), Vent (*exo*-) and T4 (*exo*-) when a hexitol scaffold (vs. deoxyribose) was used and the reverse was true for KF (*exo*-). Single incorporation studies and extension experiments (incorporation of isoGMP opposite 3 consecutive isoC nucleotides) are shown in Figures 3 and 4, respectively. Due to the similarity in the results, only incorporation studies with Pfu (*exo*-) and KF (*exo*-) are presented. With the hexitol scaffold, incorporation was limited to only two nucleotides, with the exception of T4 (*exo*-) DNA polymerase that could only incorporate one nucleotide. On the other hand, with the deoxyribose scaffold, an extension by three nucleotides was observed.

We also sought to test two evolved DNA polymerases originating from the archaeon *Thermococus gorgonarius* DNA polymerase. They were developed by the selection strategy called compartmentalised self-replication and are capable of processive DNA to HNA synthesis (Pol6G12 mutant) and HNA to DNA synthesis (RT521L mutant).[5] Utilising a hexitol scaffold, extension is only possible by two nucleotides. Accordingly we wanted to see whether these evolved DNA polymerases are capable of HNA to HNA synthesis of more than two nucleotides; however, incorporation of a third h-isoC^Me^MP (using h-isoC^Me^TP as a reagent) opposite an h-isoG nucleotide was not observed (data not shown).

We then tested the four polymerases for the incorporation of d-isoC^Me^MP and h-isoC^Me^MP opposite d-isoG and h-isoG nucleotides, respectively, in the template. Single incorporation studies, using hybrid P1:T8, showed complete primer elongation by one and two nucleotides (incorporation of d-isoC^Me^MP opposite d-isoG and a misincorporation opposite dG). On the other hand, with hybrid P1:T9, full primer extension by only a single nucleotide, except for KF (*exo*-) that could only extend 50 % of the primer, was observed. With the extension experiments (using hybrids P1:T12 and P1:T11), similar results to the previous experiments were obtained. Two and three nucleotides were incorporated with hexitol and deoxyribose, respectively, as the sugar moiety. T4 (*exo*-) polymerase, however, maintained the single nucleotide incorporation with the hexitol scaffold.

It has been extensively reported that the reduced selectivity, when using isoG in a isoC^Me^:isoG base-pairing system, arises from mispairing with T. The major cause of this is stipulated to be the tautomeric property of isoG.[14, 29] However, formation of a two-hydrogen bonded reverse wobble isoG:T base pair could be another causative factor.[30] To test the effect of hexitol scaffold in influencing selectivity, two primed (P1) templates (T8 and T9) were used to investigate the incorporation of dTMP (using dTTP as reagent) versus d-isoC^Me^MP (using d-isoC^Me^TP as reagent) against both d-isoG and h-isoG in the template. The results showed that altering the sugar moiety influences selectivity. However, the hexitol moiety has a variable effect on the selectivity of isoC^Me^:isoG base pairing (Table 4). An improvement of selectivity with the KF (*exo*-) was seen while a reduction was seen with Vent (*exo*-) and T4 (*exo*-). With Pfu (*exo*-), no effect on selectivity was observed.

**Table 4:** Quantitative data for the incorporation of dTMP and d-isoC^Me^MP opposite both d-isoG (P1:T8) and h-isoG (P1:T9) nucleotides in the template using both Vent (*exo*-) and KF (*exo*-) DNA polymerases^[a]^

	Vent (*exo*-)		KF (*exo*-)	
	dTTP [%	d-isoC^Me^TP [%]	dTTP [%]	d-isoC^Me^TP [%]
d-isoG	27.8	60.1	46.3	74
h-isoG	83.5	85.1	17.7	75.1

[a] The above values correspond to the % of extended primer using 0.5 μm of dTTP or d-isoC^Me^TP, 0.04 U μL^−1^ and 0.02 U/μL for Vent (*exo*-) and KF (*exo*-) respectively and 0.08 U μL^−1^ of TIPP. Reaction time is 2 min.

To further understand the effect that hexitol sugar could have on isoC^Me^:isoG base-pairing system, we investigated the incorporation of d-isoGMP and d-AMP, using the respective triphosphates as reagents, opposite both deoxyribose and hexitol-isoC^Me^ nucleotides in the template. Two primed (P1) templates (T2 and T3) were used in these experiments and the results are shown in Table 5. When hexitol was used instead of deoxyribose, tests with Pfu (*exo*-), Vent (*exo*-) and KF (*exo*-) demonstrated a negative effect on selectivity. On the other hand, the T4 (*exo*-) DNA polymerase shows no discrimination between dATP and d-isoGTP as a substrate; both are incorporated to equal extents regardless of the sugar moiety used. We suspected that the high degree of dAMP incorporation opposite isoC^Me^ may be due to the deamination of isoC^Me^ to T. This notion was already suggested by the group of Steven Benner.[10] They showed that 15 % of the oligonucleotides contained T instead of isoC^Me^, arising during the synthesis or deprotection step. However, analysis of our oligonucleotides carrying deoxyribose and hexitol-isoC^Me^ nucleotides did not show any deamination, even with samples of 2 years old. With deamination excluded, it seems that isoC^Me^ pairs mainly with A, most likely through a wobble base pair.[31]

**Table 5:** Quantitative data for the incorporation of dAMP and deoxy-isoGMP opposite both d-isoC^Me^ (P1:T2) and h-isoC^Me^ (P1:T3) nucleotides in the template using both Pfu (*exo*-) and T4 (*exo*-) DNA polymerases^[a]^

	Pfu (*exo*-)		T4 (*exo*-)	
	dATP [%]	d-isoGTP [%]	dATP [%]	d-isoGTP [%]
d-isoC^Me^	5.25	21.3	78.3	74.15
h-isoC^Me^	39.65	84.2	72.8	67.95

[a] The above values correspond to the % of extended primer using 20 μm (for T4 (*exo*-)) or 200 μm (for Pfu (*exo*-)) of dATP or d-isoGTP, 0.08 U μL^−1^ of Pfu (exo) and T4 (*exo*-) and 0.08 U μL^−1^ of TIPP. Reaction time is 1 for T4 (*exo*-) or 10 min for Pfu (*exo*-).

### HNA-dependent DNA synthesis in vivo

We investigated the roles of sugar modification/deoxyribose and hexitol scaffold affecting base-pairing recognition and selectivity during xenobiotic HNA-dependent DNA synthesis in vivo. To develop a successful orthogonal genetic system there should be minimal cross-recognition with natural bases, as well as specific recognition of partner bases. Accordingly, we studied the capability of templates containing the xenobiotic isoC^Me^ and isoG nucleosides in vivo, using the gapped vector assay through incorporation of sixteen DNA base/modified base mosaic oligomers (O1–O16, Table 6) to modify genetically *E. coli* cells. Oligomers were ligated into the gapped active site of the *E. coli thyA* gene located on a plasmid vector (ampicillin resistance gene *bla*^+^ containing pAK1/pAK2 heteroduplex), before transformation into a *thyA*^−^ strain of *E. coli*—a lethal genotype in thymidine deficient media.[23, 24]

**Table 6:** Sequence of the oligomers used in the HNA-dependent DNA synthesis experiments

	Sequence (5′ 3′)
O1, O2	P-CTA X*CG CCG TGC CAT GCA (X*=h-isoG, d-isoG for O1, O2, respectively)
O3, O4, O9, O10	P-CTA GCG CCG X*GC CAT GCA (X*=h-isoG, d-isoG, h-isoCMe, d-isoCMe for O3, O4, O9, O10, respectively)
O5, O6, O11, O12	P-CTA GCG CCG TGX* CAT GCA (X*=h-isoG, d-isoG, h-isoC^Me^, d-isoC^Me^ for O5, O6, O11, O12, respectively)
O7, O8	P-CTA GCG CCG TGC CX*T GCA (X*=h-isoG, d-isoG for O7, O8, respectively)
O13, O14	P-CTA GCG CCG TGX* X*AT GCA (X*=h-isoC^Me^, d-isoC^Me^ for O13, O14, respectively)
O15, O16	P-CTA GCG CCG X*GC CAX* GCA (X*=h-isoC^Me^, d-isoC^Me^ for O15, O16, respectively)

**Figure 5:**
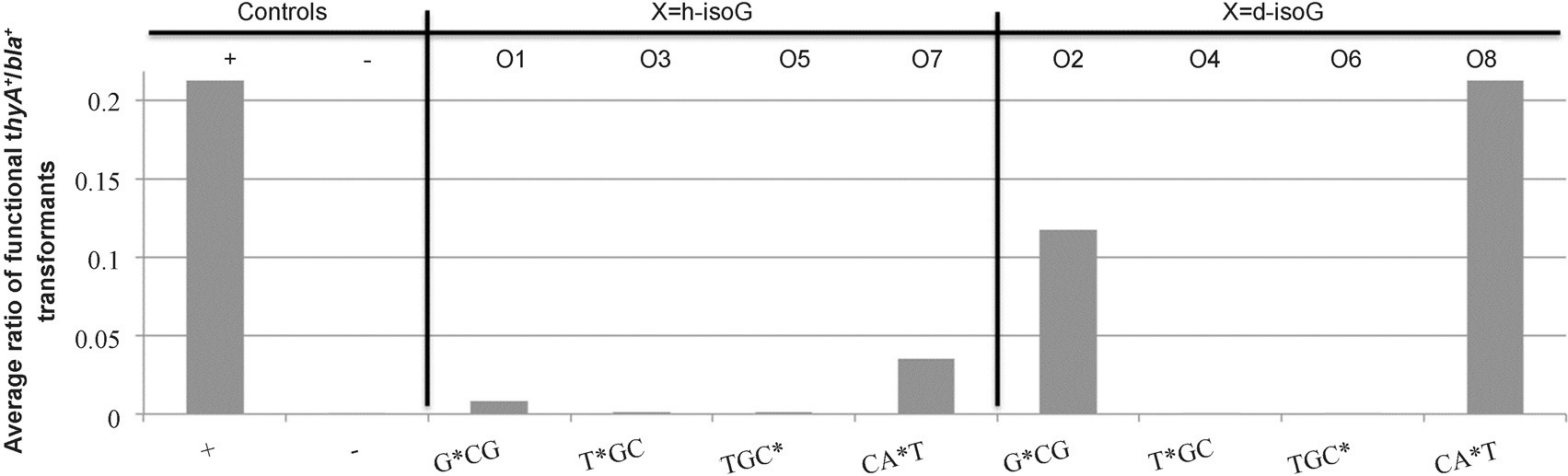
Response of the ligation of h-isoG (O1, O3, O5, O7) and d-isoG (O2, O4, O6, O8) containing oligomers into the gapped vector as a function of restoring the essential thymidylate synthase (*thyA*) gene. The ratio is taken from the number of thymidine-prototrophic colonies (*bla*^+^ *thyA*^+^) within the total number of colonies (*bla*^+^ *thyA*^−^ plus *bla*^+^ *thyA*^+^) as an average of experimental repeats. The modified part of the oligomer sequence is shown with the position of the h- and d-isoG nucleotides highlighted.

Ligation of these oligomers into the heteroduplex plasmid completes the gapped *thyA* gene. Subsequent DNA synthesis will replicate the plasmid (and the gene), which can potentially restore vitality to the strain (permitting survival without supplemental thymidine) should a functional sequence be made. The assay grants selection and quantification of the cellular interpretation of the h- and d-isoG/isoC^Me^ nucleosides. The base-pairing of a natural base with the modified nucleoside in the oligomers during DNA synthesis will cause the replacement of its equivalent partner. As a result, whatever the nucleoside is identified as by the system is what will be placed in its position in subsequent generations. The h- and d-isoG nucleosides were incorporated into oligomers in positions encoding G*CG, T*GC, TGC* and CA*T of the wild-type *thyA* active site, normally yielding Ala, Cys, Cys and His, respectively. The TGC-encoded cysteine is essential for ThyA activity. With these selections, h- and d-isoG have replaced each natural base (A, T, G, C). The response of the in vivo screen follows (Figure 5).

The *E. coli* DNA synthesis machinery appear to identify d-isoG as a G (oligomer O2: G*CG) and an A (O8: CA*T) residue, but not a T or C (in O4: T*GC/O6: TGC*). The TGC codon in both oligomers codes for the active site Cys residue—an essential amino acid in the enzyme that results in a lethal phenotype when changed.[23, 24] It is therefore likely that d-isoG is being strongly recognised as an A and less so a G whilst also being not recognised as a T or C as any recognition for these latter bases would recover a non-lethal phenotype. The response of the equivalent hexitol scaffold nucleosides follows largely the same pattern—higher recognition as A then G, and not at all as T or C, although proportionally the hexitol scaffold is far less recognised.

This demonstrates that the modifications of the backbone from deoxyribose to hexitol increases the potential orthogonality of the isoG base, as the hexitol backbone further reduces the recognition of isoG by the natural nucleosides. In both cases, there seems to be a distinct lack of recognition as T and C which indicates that isoG is not base pairing with A/G, but instead with T primarily, with both backbone scaffolds. IsoG gives a mispairing order of T>C≫A=G, matching well the in vitro findings.

**Figure 6:**
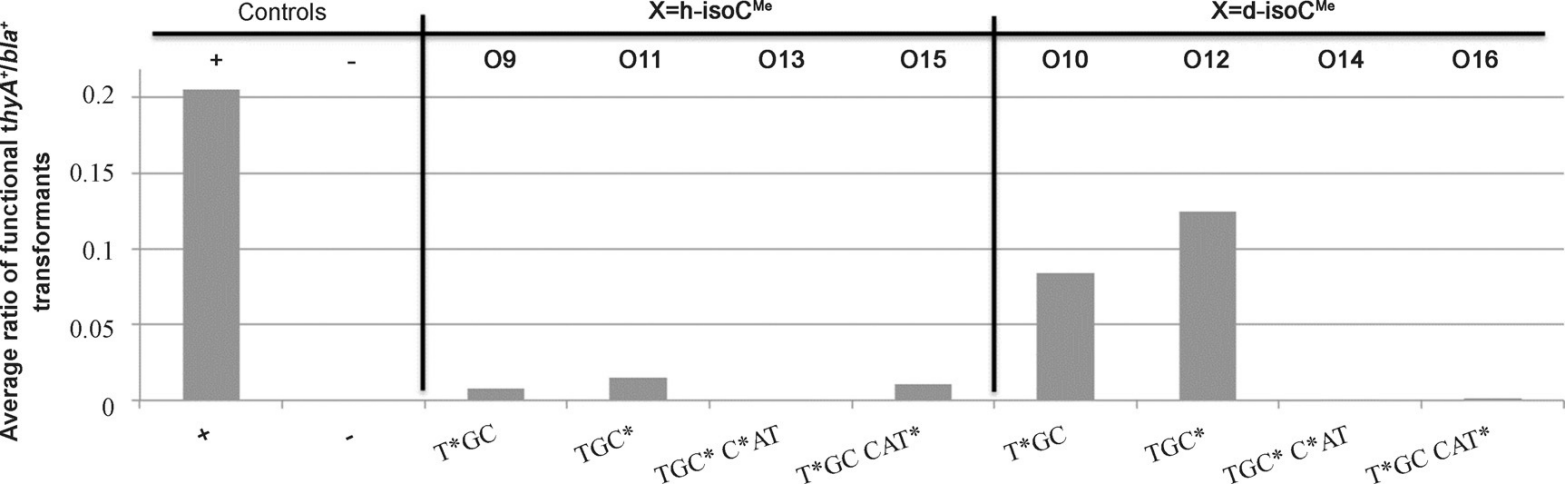
Response of the ligation of h-isoC^Me^ (O9, O11, O13, O15) and d-isoC^Me^ (O10, O12, O14, O16) containing oligomers into the gapped vector as a function of restoring the essential thymidylate synthase (*thyA*) gene. The ratio is taken from the number of thymidine-prototrophic colonies (*bla*^+^ *thyA*^+^) against the total number of colonies (*bla*^+^ *thyA*^−^ and *bla*^+^ *thyA*^+^) as an average of experimental repeats. The modified part of the oligomer sequence is shown with the position of the incorporated h- and d-isoC^Me^ nucleotide/s highlighted.

The h- and d-isoC^Me^ nucleosides were also studied, through incorporation into mosaic oligomers which encode T*GC, TGC*, TGC* C*AT and T*GC CAT* of the *thyA* active site, naturally yielding the essential Cys in all TGC codons and His in the CAT codons. Here, h- and d-isoC^Me^ have replaced single or double T and/or C bases in the oligomers (Figure 6).

In common to previous data, the recognition of hexitol scaffold nucleosides is generally reduced in comparison to deoxyribose scaffold nucleosides. The d-isoC^Me^ nucleoside appears to be recognised mostly as a C and then T, agreeing with the previous in vitro data showing the base pairing of this nucleoside with G most strongly, although in both double incorporations (TGC* C*AT and T*GC CAT*) there was virtually no recognition even as a C (for pairing with G). It would seem that double incorporations are terminal in this system, and are not able to be recognised correctly to permit survival as both T and C residues. The results from the single incorporation hexitol scaffold isoC^Me^ follow generally the pattern of the deoxyribose forms but with lower responses, with the exception of the double incorporation oligomer (T*GC CAT*), which is the only oligomer of those tested where there is apparently higher recognition of the hexitol scaffold relative to deoxyribose. Interpretation of this result against the single incorporations T*GC and TGC* and the double incorporation TGC* C*AT suggest that even if the h- and d-isoC^Me^ nucleosides are mainly base-pairing G (thus being recognised as C) the presence of two adjacent molecules will corrupt this recognition. For both h- and d-isoC^Me^, the survival phenotype is lethal when two of the nucleosides are adjacent.

With these results considered, it was interesting to observe what bases may would have been included in the site of the h- and d-isoG/isoC^Me^ incorporations, as some of the modified codons had permissible encoding redundancies. Thus, colonies that were positive for growth in the absence of thymidine (*bla*^+^ *thyA*^+^) were confirmed twice by restreaking, before plasmid extraction and sequencing. Sequence data from the regions of interest are analysed below (Table 7).

**Table 7:** Sequences of modified codons recovered by DNA sequencing of plasmids from *thyA*^*+*^ strains^[a]^

	Vent (*exo*-)		KF (*exo*-)	
Wild-type codon	h-isoC^Me^	d-isoC^Me^	h-isoG	d-isoG
T*GC	100 % T*GC (Cys)	100 % T*GC (Cys)	100 % T*GC (Cys)	lethal
TGC*	**18 %**­ TGT*­ **(Cys)** 82 % TGC* (Cys)	**100 %**­ TGT*­ **(Cys)**	**13 %**­ TGT*­ **(Cys)** 87 % TGC* (Cys)	lethal
TGC* C*AT	**13 %**­ TGT*­ **(Cys)** 87 % TGC* (Cys)	**58 %**­ TGT*­ **(Cys)** 42 % TGC* (Cys)	N/A	N/A
TGC* C*AT	100 % C*AT (His)	100 % C*AT (His)	N/A	N/A
T*GC CAT*	100 % T*GC (Cys)	100 % T*GC (Cys)	N/A	N/A
T*GC CAT*	100 % CAT* (His)	100 % CAT* (His)	N/A	N/A
G*CG	N/A	N/A	**7 %**­ C*CG­ **(Pro) 7 %**­ A*CG­ **(Thr)** 86 % G*CG (Ala)	**100 %**­ A*CG­ **(Thr)**
CA*T	N/A	N/A	100 % CA*T (His)	100 % CA*T (His)
T*GC	100 % *GC (Cys)	100 % T*GC (Cys)	100 % T*GC (Cys)	lethal

[a] Percentages came from the occurrence of the sequence relative to the number of colonies (*bla*^+^ *thyA*^+^) analysed (typically 16 as minimum). The sequence at the site of incorporation in the oligomer is shown, with the codon under sequence analysis underlined. Deviations from wild-type sequence are bolded and the amino acid coded by the codon (unchanged or otherwise) is indicated in all cases.

With the h- and d-isoC^Me^ bases, the only changes observed were found in the codons encoding the essential active site Cys (T*GC and TGC*). In the former, only T is acceptable, and from the surviving cells only a T was recovered in this position for both bases. In the latter, it was found that for d-isoC^Me^ there was the conversion of the C to a T, confirming more stable pairing with A than G, which occurred at higher percentage rates than with h-isoC^Me^. Both C and T are permissible here, as it must encode unconditionally Cys (TGC or TGT). In the His encoding position (C*AT) no changes were found, which indicated some recognition of the bases as G, although for both nucleosides their incorporation here was deleterious to the survival. It is not yet known what changes to the C*AT (His) motif affect survival, and the extent of what other amino acids are tolerable in this position. Recognition of d-isoC^Me^ as T (base pairing A) is greater than for h-isoC^Me^, indicated by both the in vivo response as well as sequencing data.

The h- and d-isoG bases showed a wider range of changes. In particular there was strikingly strong recognition and resultant misincorporation caused by d-isoG, causing full conversion of G to A in the Ala codon G*CG—changing the amino acid to Thr, resulting in approximately half of the survival rate compared to the ratio of the positive controls. In contrast, the h-isoG sample population showed some change from G to A, as well as to C (encoding Pro, CCG), although it was dominantly recognised as the wild-type G, and in vivo recognition was low.

## Conclusions

We have developed an efficient route for the synthesis of oligonucleotides containing h-isoG and h-isoC^Me^ nucleotides synthesised on solid-phase support by using phosphoramidite building blocks **13** and **25**, respectively. The required h-isoG phosphoramidite **13** was obtained by nucleophilic displacement of 2-aminoadenine on 2-*O*-tosylate of 1,5-anhydro-4,6-*O*-benzylidene-d-glucitol **7** followed by selective deamination and usual protection with DMTr and phosphitylation. The amidite of h-isoC^Me^ **25** has been synthesised from commercially available 1,5:2,3-dianhydro-4,6-*O*-benzylidene-d-allitol, through the formation of an intermediate anhydro sugar **18** that was regioselectively opened with ammonia as a key step. The base-pairing and mismatch discrimination of hexitol-isoG and hexitol-isoC^Me^ in duplex DNA with antiparallel chain orientation was determined by *T*_m_ measurements. The h-isoG and h-isoC^Me^ base-pair engrafted in a double-strand DNA was found to be less stable compared d-isoG:d-isoC^Me^ (Δ*T*_m_=−3.6) base pair, which might be due to the comparison of a mixed backbone duplex (HNA/DNA) versus a fully DNA duplex. Incorporation of a h-isoG:h-isoC^Me^ base pair within a fully HNA duplex should therefore be investigated. The base-pair stability of h-isoG decreases in the order d-isoC^Me^≈h-isoC^Me^>G≥C≫T>A, whereas for d-isoG is d-isoC^Me^>h-isoC^Me^>T>G>C≫A. The HNA sugar showed slightly better mismatch discrimination against dT and increased the selectivity opposite to h-isoC^Me^ compared to deoxyribose sugar. The incorporation studies have shown that a hexitol scaffold carrying an isoC^Me^:isoG base pair can be recognised and incorporated by a selection of polymerases, most favourably the Family B DNA polymerases. However, incorporation of more than two nucleotides with hexitol nucleic acids was not possible.[3a] Sugar modifications may influence the selectivity of a base pair. However, this alteration is polymerase-dependent. Our selected sugar, hexitol, is capable of both improving and reducing the selectivity of the isoC^Me^:isoG base pair. Thus altering the sugar moiety does not seem like being a generic solution for improving the isoC^Me^:isoG base-pairing selectivity. The in vivo and sequencing experiments show the base-pair stabilities of the h- and d-isoG/C^Me^ nucleotides in a genetic context. The penalty to cell survival in the screens was proportional to the level of recognition of the nucleosides as the natural bases. This was established through the in vivo stabilities of base pairing made by the incorporated nucleotides, and as suggested by the in vitro data obtained, this would also depend on the nature of the polymerase. Limited codon redundancies in some positions exposed silent changes in the structure of the genetic code of the *thyA* gene, as well as in the translated enzyme, whereas others generated codon and subsequent amino acid modifications that affected survival response. The role of the hexitol scaffold showed potency for loss of recognition compared to the deoxyribose scaffold, demonstrating further steps towards orthogonality (which seems to be reached with the double h-isoC^Me^ incorporation experiment) that will encourage and inspire future developments towards an orthogonal XNA-based information system.

## Experimental Section

^1^H, ^13^C and ^31^P NMR spectra were recorded on 300 (^1^H, 300; ^13^C, 75; ^31^P NMR, 121 MHz), 500 (^1^H, 500; ^13^C NMR, 125 MHz), or 600 MHz (^1^H, 600; ^13^C NMR, 150 MHz) spectrometers. 2D NMRs (H**-**COSY, HSQC and HMBC) were used for the assignment of all the intermediates and final compounds. Mass spectra were acquired on a quadrupole orthogonal acceleration time-of-flight mass spectrometer. Column chromatographic separations were carried out by gradient elution with suitable combination of two/three solvents and silica gel (100–200 mesh or 230–400 mesh). Solvents for reactions were distilled prior to use: THF and toluene from Na/benzophenone; CH_2_Cl_2_ and CH_3_CN from CaH_2_; Et_3_N and pyridine from KOH.

### 1′,5′-Anhydro-4′,6′-*O*-benzylidene-2′,3′-dideoxy-2′-(2,6-diaminopurin-9-yl)-d-arabino-hexitol (8)

A mixture of 2,6-diaminopurine (2.3 g, 15.37 mmol) and sodium hydride 60 % in mineral oil (409 mg, 10.24 mmol) in anhydrous DMF (20 mL) was stirred at room temperature for 30 min under nitrogen. Then, the tosylate sugar **7** (2.0 g, 5.12 mmol) was added. The temperature was raised up to 90 °C and stirring was continued for 12 h. The reaction mixture was then cooled and evaporated to dryness. The residue was dissolved in ethyl acetate (100 mL), and the organic layer was washed with a saturated solution of NaHCO_3_ (3×30 mL). The organic layer was dried over sodium sulphate, filtered and evaporated to dryness. The crude material was purified by flash column chromatography on silica gel (CH_2_Cl_2_/MeOH, 20:1) to give **8** (1.5 g, 79 %) as a white solid. UV (MeOH): *λ*_max_=256, 283 nm; ^1^H NMR (600 MHz, [D_6_]DMSO): *δ*=7.88 (s, 1 H, H_8_), 7.38–7.33 (m, 5 H, Ar-H), 6.77 (s, 2 H, NH_2_), 5.88 (s, 2 H, NH_2_), 5.63 (s, 1 H, PhCH), 4.65 (br s, 1 H, H_2_′), 4.35 (d, *J*=13.2 Hz, H_1b_′), 4.21 (dd, *J*=10.3, 4.9 Hz, 1 H, H_6b_′), 4.05 (dd, *J*=13.2, 2.7 Hz, 1 H, H_1a_′), 3.78 (t, *J*=10.3 Hz, 1 H, H_6a_′), 3.72 (ddd, *J*=12.6, 9.7, 4.3 Hz, 1 H, H_4_′), 3.50 (td, *J*=9.7, 4.9 Hz, 1 H, H_5_′), 2.42 (m, 1 H, H_3b_′), 2.07 ppm (td, *J*=8.5, 4.3 Hz, 1 H, H_3a_′);^13^C NMR (151 MHz, [D_6_]DMSO): *δ*=160.3 (C6), 156.2 (C2), 151.6 (C4), 137.7 (Ipso), 135.7 (C8), 128.7, 127.9 (s), 126.1 (s) (Ar-C), 112.8 (C5), 100.8 (PhCH), 73.7 (C5′), 73.4 (C4′), 68.9 (C1′), 68.0 (C6′), 49.6 (C2′), 32.1 ppm (C3′); HRMS (ESI+): calcd for C_18_H_20_N_6_O_3_: 369.1669 [*M*+H]^+^; found: 369.1669.

### 1′,5′-Anhydro-2′,3′-dideoxy-2′-(2,6-diaminopurin-9-yl)-d-arabino-hexitol (9)

A solution of compound **8** (1.3 g, 3.53 mmol) in CH_3_COOH/H_2_O (40 mL, 6:4) was stirred at 60 °C for 4 h. The mixture was evaporated under reduced pressure and co-evaporated with toluene. The crude material was purified by flash column chromatography (CH_2_Cl_2_/MeOH, 9:1) to give **9** (903 mg, 91 %) as a white solid. UV (MeOH): *λ*_max_=256, 281 nm. ^1^H NMR (500 MHz, [D_6_]DMSO): *δ*=7.91 (s, 1 H, H_8_), 6.71 (s, 2 H, NH_2_), 5.84 (s, 2 H, NH_2_), 4.56 (s, 1 H, H_2_′), 4.13 (d, *J*=12.5 Hz 1 H, H_1b_′), 3.81 (dd, *J*=12.5, 2.4 Hz 1 H, H_1a_′), 3.69 (dd, *J*=11.5, 1.3 Hz 1 H, H_6b_′), 3.58 (dd, *J*=11.5, 4.9 Hz 1 H, H_6a_′), 3.51 (td, *J*=6.0, 4.8 Hz 1 H, H_4_′), 3.14–3.19 (m, 1 H, H_5_′), 2.20–2.26 (m, 1 H, H_3b_′), 1.81 ppm (td, *J*=13.2, 4.8 Hz 1 H, H_3a_′); ^13^C NMR (125 MHz, [D_6_]DMSO): *δ*=160.2 (C6), 156.1 (C2), 151.6 (C4), 136.2 (C8), 112.6 (C5), 83.0 (C5′), 68.1 (C1′), 60.8 (C5′), 60.5 (C6′), 49.3 C2′), 36.0 ppm (C3′); HRMS (ESI+): calcd for C_11_H_16_N_6_O_3_: 281.1356 [*M*+H]^+^; found: 281.1360.

### 1′,5′-Anhydro-2′,3′-dideoxy-2′-(6-amino-2-hydroxy-purin-9-yl)-d-arabino-hexitol (2)

The diamino compound **9** (800 mg, 2.85 mmol) was suspended in H_2_O (20 mL) at 55 °C and a solution of NaNO_2_ (768 mg, 11.13 mmol) in H_2_O (5 mL) was added dropwise. Then AcOH (1.1 mL, 19.98 mmol) was added over 2 min. The resulting clear solution was stirred for 10 min, then cooled to room temperature and diluted with H_2_O (15 mL), and conc. aq. NH_3_ solution was added to adjust the pH 8. The solution was evaporated and the remaining solid was washed with H_2_O to give a yellow solid. The crude material was purified by flash column chromatography (CH_2_Cl_2_/MeOH, 3:1) to give **2** (612 mg, 76 %) as a yellow solid. UV (MeOH): *λ*_max_=249, 298 nm; ^1^H NMR (600 MHz, [D_6_]DMSO): *δ*=7.94 (s, 1 H, H_8_), 4.90 (d, *J*=4.9 Hz, 1 H, OH), 4.64 (br s, 1 H, H_2_′), 4.52 (s, 1 H, OH), 4.09 (d, *J*=12.6 Hz, 1 H, H_1b_′), 3.79 (d, *J*=12.6, 2.6 Hz, 1 H, H_1a_′), 3.68 (d, *J*=11.6 Hz, 1 H, H_6b_′), 3.59–3.54 (m, 1 H, H_6a_′), 3.53–3.48 (m, 1 H, H_4_′), 3.18–3.13 (m, 1 H, H_5_′), 2.21–2.17 (m, 1 H, H_3b_′), 1.82–1.76 ppm (m, 1 H, H_3a_′); ^13^C NMR (75 MHz, [D_6_]DMSO): *δ*=156.5, 156.4 (C2, C6), 152.2 (C4), 138.1 (C8), 108.7 (C5), 83.1 (C5′), 68.1 (C1′), 60.8, 60.6 (C4′, C6′), 49.5 (C2′), 35.9 ppm (C3′); HRMS (ESI+): calcd for C_11_H_15_N_5_O_4_: 282.1196 [*M*+H]^+^; found: 282.1197.

### 1′,5′-Anhydro-2′,3′-dideoxy-2′-[6-(dimethylamino)methyl-idene-2-hydroxy-purin-9-yl]-d-arabino-hexitol (10)

*N*,*N*-Dimethylformamide dimethyl acetal (1.1 mL, 2.2 mmol) was added to a suspension of **2** (500 mg, 1.7 mmol) in MeOH (20 mL), and the resulting mixture stirred at 25 °C for 12 h. The reaction mixture was evaporated to dryness and purified by flash column chromatography (CH_2_Cl_2_/MeOH, 4:1) to give **10** (480 mg, 80 %) as a white solid. UV (MeOH): *λ*_max_=262, 345 nm; ^1^H NMR (600 MHz, [D_6_]DMSO): *δ*=9.21 (s, 1 H, N=CH), 8.08 (s, 1 H, H_8_), 4.54 (s, 1 H, H_2_′), 4.10 (d, *J*=12.6 Hz, 1 H, H_1b_′), 3.81 (dd, *J*=12.6, 2.3 Hz, 1 H, H_1a_′), 3.67 (dd, *J*=11.8, 1.98 Hz, 1 H, H_6b_′), 3.59 (dd, *J*=11.8, 4.5 Hz, 1 H, H_6a_′), 3.51 (ddd, *J*=11.0, 9.4, 4.9 Hz, 1 H, H_4_′), 3.20 (s, 3 H, NCH_3_), 3.18–3.15 (m, 1 H, H_5_′), 3.09 (s, 3 H, NCH_3_), 2.24–2.18 (m, 1 H, H_3b_′), 1.83–1.76 ppm (m, 1 H, H_3a_′); ^13^C NMR (150 MHz, [D_6_]DMSO): *δ*=161.4 (N=C), 157.4 (C4), 156.7 (C2), 154.3 (C6), 140.7 (C8), 112.4 (C5), 83.0 (C5′), 67.9 (C1′), 60.5 (C4′), 60.4 (C-6′), 49.4 (C2′), 41.1 (N-CH_3_), 35.6 (C3′), 34.3 ppm (N–CH_3_); HRMS (ESI+): *m*/*z*: calcd for C_14_H_20_N_6_O_4_: 337.1618 [*M*+H]^+^; found: 337.1619.

### 1′,5′-Anhydro-2′,3′-dideoxy-2′-[6-*N*-(dimethylamino)methyl-idene-2-*O*-(diphenyl-carbamoyl)purin-9-yl]-d-arabino-hexitol (11)

Diphenylcarbamoyl chloride (350 mg, 1.04 mmol) and *N*,*N*-diisopropylethylamine (0.71 mL, 1.35 mmol) was successively added to a suspension of **10** (350 mg, 1.04 mmol) in dry pyridine (15 mL). The reaction mixture was stirred for 1 h at 25 °C. Then, the mixture was poured into 5 % aqueous solution NaHCO_3_ (10 mL) and extracted with CH_2_Cl_2_ (3×10 mL). The combined organic layers were dried over Na_2_SO_4_ and filtered. After evaporation of the solvent, the residue was purified by flash column chromatography (CH_2_Cl_2_/MeOH, 20:1) to give **11** (420 mg, 76 %) as a pink solid. UV (MeOH): *λ*_max_=236, 315 nm; ^1^H NMR (500 MHz, [D_6_]DMSO): *δ*=8.90 (s, 1 H, N=CH), 8.41 (s, 1 H, H_8_), 7.46–7.40 (m, 8 H, ArH). 7.26–7.35 (m, 2 H, ArH), 4.94 (d, *J*=5.4 Hz, 1 H, OH), 4.77 (s, 1 H, H_2_′), 4.68 (t, *J*=6.2 Hz, 1 H, OH), 4.20 (d, *J*=12.7 Hz, 1 H, H_1b_′), 3.86 (dd, *J*=12.7, 2.5 Hz, 1 H, H_1a_′), 3.69 (ddd, *J*=7.7, 6.2, 2.3 Hz, 1 H, H_6b_′), 3.57–3.56 (m, 1 H, H_6a_′), 3.52 (ddd, *J*=14.2, 10.4, 5.4 Hz, 1 H, H_4_′), 3.22–3.18 (m, 4 H, NCH_3_, H_5_′), 3.13 (s, 3 H, NCH_3_), 2.29–2.24 (m, 1 H, H_3b_′), 1.90 ppm (ddd, *J*=13.6, 10.4, 4.15 Hz, 1 H, H_3a_′); ^13^C NMR (125 MHz, [D_6_]DMSO): *δ*=160.5 (C6), 158.8 (HC=N), 155.3 (C2), 152.5 (C4), 151.6 (C=O), 141.9 (C8), 129.2, 127.1, 126.8 (Ar), 123.0 (C5), 82.9 (C5′), 67.8 (C1′), 60.6 (C4′), 60.4 (C6′), 50.3 (C2′), 40.8 (N-CH_3_), 35.8 (N-CH_3_), 34.6 ppm (C3′); HRMS (ESI+): calcd for C_27_H_29_N_7_O_5_: 532.2302 [*M*+H]^+^; found: 532.2303.

### 1′,5′-Anhydro-2′,3′-dideoxy-2′-[6-*N*-(dimethylamino)methyl-idene-2-O-(diphenyl-carbamoyl)purin-9-yl]-6′-*O*-dimethoxy trityl-d-arabino-hexitol (12)

Compound **11** (1.05 g, 0.75 mmol) was dried by co-evaporation with pyridine (3×10 mL), and was then dissolved in dry pyridine (20 mL). 4,4′-Dimethoxytrityl chloride (305 mg, 0.90 mmol) was added in three portions (each every 10 min) at 0 °C under stirring. The mixture was warmed up to room temperature and stirred for 12 h. After addition of MeOH (5 mL), the reaction mixture was evaporated and the residue was dissolved in CH_2_CI_2_ (50 mL). The solution was washed with 5 % NaHCO_3_ aqueous solution (10 mL) and H_2_O (10 mL), dried over Na_2_SO_4_ and the organic layer was evaporated to afford an oil. The crude material was purified by flash column chromatography (CH_2_Cl_2_/MeOH/TEA, 98:2:1) to give **12** (549 mg, 73 %) as a white foam. UV (MeOH): *λ*_max_=234, 316 nm; ^1^H NMR (500 MHz, [D_6_]DMSO): *δ*=8.98 (s, 1 H, N=CH), 8.42 (s, 1 H, H_8_), 7.44–7.21 (m, 19 H, Ar), 6.90–6.86 (m, 4 H, Ar), 4.87 (d, *J*=5.8 Hz, 1 H, OH), 4.77 (s, 1 H, H_2_′), 4.34 (d, *J*=12.8 Hz, 1 H, H_1b_′), 3.93 (d, *J*=12.8 Hz, 1 H, H_1a_′), 3.74 (s, 3 H, 3 CH_3_), 3.73 (s, 3 H, 3 CH_3_), 3.57–3.52 (m, 1 H, H_4_′), 3.12–3.38 (m, 10 H, H_5_′, H_6a_′, H_6b_′, 2 NCH_3_), 2.38–2.33 (m, 1 H, H_3b_′), 1.93–1.86 ppm (m, 1 H, H_3a_′); ^13^C NMR (150 MHz, CDCl_3_): *δ*=160.8 (C6), 158.8 (C=N), 158.5, 156.0 (C2, Ar), 152.8, 152.2 (C4, C=O), 144.2, 142.1 (Ar), 141.3 (C8), 135.4, 135.4, 129.8, 128.8, 127.9, 127.9, 126.9 (Ar), 123.8 (C5), 113.2 (Ar), 86.8 (CPh_3_), 80.4 (C4′), 69.1 (C1′), 64.6 (C5′), 55.15 (OCH_3_), 53.2 (C6′), 50.1 (C2′), 41.3 (NCH_3_), 35.8 (C3′), 35.2 ppm (NCH_3_); HRMS (ESI+): calcd for C_48_H_47_N_7_O_7_: 834.3609 [*M*+H]^+^; found: 834.3614.

### 1′,5′-Anhydro-2′,3′-dideoxy-2′-[6-*N*-(dimethylamino)methyl-idene-2-O-(diphenyl-carbamoyl)purin-9-yl]-6′-*O*-dimethoxy trityl-d-arabino-hexitol-4′-(2-cyanoethyl-*N*,*N*-diisopropyl)phosphoramidite (13)

A 0.45 molar solution of 1*H*-tetrazole (1.0 mL, 0.47 mmol) was added dropwise to a mixture of 1 m cyanoethyl-bis(*N*,*N*-diisopropylamino)phosphine (0.87 mL, 0.87 mmol) and **12** (330 mg, 0.39 mmol) in CH_2_Cl_2_ (15 mL) at 0 °C, and the reaction mixture was allowed to stir at room temperature for 1 h. The reaction mixture was diluted with CH_2_Cl_2_ (40 mL) and washed with 5 % NaHCO_3_ aqueous solution (10 mL). The organic layer was dried over Na_2_SO_4_ and concentrated. The crude mixture was quickly purified by flash column chromatography (*n*-hexane/acetone/TEA, 50:50:1) to give diastereomeric mixture **13** (305 mg, 75 %) as a white foam. ^31^P NMR (202 MHz, CDCl_3_): *δ*=149.3, 148.5 ppm; ^1^H NMR (500 MHz, CDCl_3_): *δ*=9.02 (s, 1 H, N=CH), 8.45 (s, 1 H, H_8_), 7.50–7.19 (m, 19 H, Ar), 6.85–6.80 (m, 4 H, Ar), 4.91 (s, 1 H, H_2_′), 4.38 (d, *J*=13.0 Hz, 1 H, H_1b_′), 4.04–3.97 (m, 1 H, H_1b_′), 3.89–3.78 (m, 7 H, 2 OCH_3_, H_4_′), 3.72–3.14 (m, 13 H, OCH_2_, H_6a_′, H_6b_′, 2 NCH_3_, H_5_′, 2 CHN), 2.69–2.59 (m, 1 H, H_3b_′), 2.22 (t, *J*=6.4 Hz, 2 H, CH_2_CN), 2.04–1.90 (m, 1 H, H_3a_′), 1.05 −0.81 ppm (m, 12 H, 4 CH_3_); HRMS (ESI+): calcd for C_57_H_64_N_9_O_8_P: 1034.4687 [*M*+H]^+^; found 1034.4692.

### 1′,5′-Anhydro-4′,6′-*O*-benzylidene-2′-deoxy-2′-(5-methyl-isocytosin-1-yl)-d-manno-hexitol (19)

A solution of compound **17** (1.0 g, 2.28 mmol) in methanolic ammonia (saturated at 0 °C, 50 mL) was heated at 160 °C for 60 h in a sealed tube. After cooling down at room temperature the reaction mixture was concentrated, and the residue was purified by flash column chromatography (CH_2_Cl_2_/MeOH, 20:1) to give **22** (580 mg, 70 %) as a white solid. UV (MeOH): *λ*_max_=260; ^1^H NMR (500 MHz, [D_6_]DMSO): *δ*=7.73 (s, 1 H, H_6_), 7.49–7.35 (m, 5 H, Ar), 5.69 (s, 1 H, CHPh), 4.53 (br s, 1 H, H_2_′), 4.22 (dd, *J*=6.1, 2.9 Hz, 1 H, H_6b_′), 4.19 (d, *J*=8.1 Hz, 1 H, H_1b_′), 4.12 (dd, *J*=5.9, 3.3 Hz, 1 H, H_3_′), 4.04 (dd, *J*=8.1, 2.1 Hz, 1 H, H_1a_′), 3.85 (t, *J*=6.1 Hz, 1 H, H_6a_′), 3.78 (t, *J*=5.9 Hz, 1 H, H_4_′), 3.52 (td, *J*=5.9, 2.9 Hz, 1 H, H_5_′), 1.80 ppm (s, 1 H, CH_3_); ^13^C NMR (125 MHz, [D_6_]DMSO): *δ*=170.1 (C4), 157.0 (C2), 137.7 (Ar), 135.7 (C6), 128.9, 128.0, 126.3 (Ar), 114.6 (C5), 101.0 (CPh), 77.7 (C4′), 71.6 (C5′), 68.7 (C3′), 68.3 (C1′), 67.5 (C6′), 57.3 (C2′), 14.1 ppm (CH_3_); HRMS (ESI+): calcd for C_18_H_21_N_3_O_5_: 360.1553 [*M*+H]^+^; found: 360.1555.

### 1′,5′-Anhydro-4′,6′-*O*-benzylidene-2′-deoxy-2′-[6-(Dimethyl-amino)methylidene-5-methyl-isocytosin-1-yl)-d-manno-hexitol (20)

*N*,*N*-Dimethylformamide dimethyl acetal (0.37 mL, 2.78 mmol) was added to a suspension of **19** (500 mg, 1.39 mmol) in MeOH (20 mL). The reaction mixture was stirred at 65 °C for 3 h. The reaction mixture was evaporated to dryness and purified by flash column chromatography (CH_2_Cl_2_/MeOH, 97:3) to give **20** (510 mg, 88 %) as a white solid. UV (MeOH): *λ*_max_=247, 280 nm; ^1^H NMR (500 MHz, CDCl_3_): *δ*=8.82 (s, 1 H, N=CH), 7.97 (s, 1 H, H_6_), 7.50–7.30 (m, 5 H, Ar), 5.63 (s, 1 H, CHPh), 5.26–5.22 (m, 1 H, H_2_′), 4.37 (dd, *J*=6.3, 2.9 Hz, 1 H, H_6b_′), 4.34 (dd, *J*=5.8, 3.2 Hz, 1 H, H_3_′), 4.27 (d, *J*=8.3 Hz, 1 H, H_1b_′), 4.06 (dd, *J*=8.3, 2.1 Hz, 1 H, H_1a_′), 3.85 (t, *J*=6.3 Hz, 1 H, H_6a_′), 3.79 (t, *J*=6.3 Hz, 1 H, H_4_′), 3.58 (td, *J*=5.8, 2.9 Hz, 1 H, H_5_′), 3.18 (s, 3 H, NCH_3_), 3.06 (s, 3 H, NCH_3_), 2.05 ppm (s, 3 H, CH_3_);^13^C NMR (126 MHz, CDCl_3_): *δ*=171.7 (C4), 159.2 (C2), 158.9 (C=N), 137.1 (Ar), 136.3 (C6), 129.2, 128.3, 126.4 (Ar), 119.0 (C5), 102.2 (CHPh), 78.3 (C4′), 72.2 (C5′), 71.3 (C3′), 68.5, 68.2 (C6′, C1′), 56.8 (C2′), 41.9, 35.8 (NCH_3_), 14.6 ppm (CH_3_); HRMS (ESI+): calcd for C_21_H_26_N_4_O_5_: 415.1975 [*M*+H]^+^; found: 415.1978.

### 1′,5′-Anhydro-4′,6′-*O*-benzylidene-3-*O*-(imidazol-1-ylthiocarbonyl)-2′-deoxy-2′-[6-(di-methylamino)methylidene-5-methyl-isocytosin-1-yl)-d-manno-hexitol (21)

1,1′-Thiocarbonyldiimidazole (316 mg, 1.77 mmol) was added to a solution of compound **20** (490 mg, 1.18 mmol) in DMF. The reaction mixture was stirred for 12 h at room temperature. The reaction mixture was diluted with ethyl acetate (100 mL) and washed with H_2_O (3×10 mL). The organic layer was dried over Na_2_SO_4_ and evaporated. The residue was purified by flash column chromatography (CH_2_Cl_2_/MeOH, 97:3) to give **21** (549 mg, 88 %) as a yellow solid. UV (MeOH): *λ*_max_=247, 280 nm; ^1^H NMR (500 MHz, CDCl_3_): *δ*=8.26 (s, 1 H, CH=N), 8.24 (s, 1 H, Im-H), 8.07 (s, 1 H, H_6_), 7.42–7.30 (m, 5 H, Ar), 7.23 (s, 1 H, Im-H), 6.98 (s, 1 H, Im-H), 6.43 (dd, *J*=10.5, 5.5 Hz, 1 H, H_3_′), 5.87 (dd, *J*=5.5, 3.5 Hz, 1 H, H_2_′), 5.61 (s, 1 H, CHPh), 4.47–4.40 (m, 2 H, H_6b_′, H_1b_′), 4.25 (dd, *J*=13.9, 3.5 Hz, 1 H, H_1a_′), 4.18 (dd, *J*=10.5, 9.6 Hz, 1 H, H_4_′), 3.92 (t, *J*=10.5 Hz, 1 H, H_6a_′), 3.77 (td, *J*=9.6, 4.9 Hz, 1 H, H_5_′), 2.90 (s, 3 H, NCH_3_), 2.82 (s, 3 H, NCH_3_), 2.09 ppm (s, 3 H, CH_3_); ^13^C NMR (125 MHz, CDCl_3_): *δ*=183.8 (C=S), 171.4 (C4), 159.0 (C2), 158.6 (C=N), 138.3 (Im-C), 136.4 (Ar), 135.8 (C6), 131.3 (Im-C), 129.4, 128.3, 126.2 (Ar), 118.7 (Im-C), 116.8 (Im-C), 102.1 (CHPh), 78.1 (C3′), 74.9 (C4′), 73.0 (C5′), 69.1 (C1′), 68.4 (C6′), 52.5 (C2′), 41.4 (NCH_3_), 35.4 (NCH_3_), 14.7 ppm (CH_3_); the HRMS analysis showed degradation of **21** to compound **20**.

### 1′,5′-Anhydro-4′,6′-*O*-benzylidene-2′,3′-dideoxy-2′-[6-(dimethylamino)methylidene-5-methyl-isocytosin-1-yl]-d-arabino-hexitol (22)

*n*Bu_3_SnH (0.81 mL, 3.03 mmol) was added at room temperature to a solution of **21** (510 mg, 1.01 mmol) and AIBN (82 mg. 0.50 mmol) in anhydrous and degassed toluene (40 mL). This solution was degassed three times under argon. The reaction mixture was heated at 80 °C for 3 h. The volatile was evaporated under reduced pressure, and the residue was purified by flash column chromatography (CH_2_Cl_2_/MeOH, 97:3) to give **22** (280 mg, 89 %) as a thick liquid. UV (MeOH): *λ*_max_=247, 282 nm; ^1^H NMR (500 MHz, CDCl_3_): *δ*=8.86 (s, 1 H, CH=N), 7.87 (s, 1 H, H_6_), 7.47–7.33 (m, 5 H, Ar), 5.59 (s, 1 H, CHPh), 5.08 (br s, 1 H, H_2_′), 4.36 (dd, *J*=10.6, 4.9 H, 1 H, H_6b_′), 4.31 (d, *J*=13.7 Hz, 1 H, H_1b_′), 4.01(dd, *J*=13.7, 3.6 Hz, 1 H, H_1a_′), 3.81 (t, *J*=10.6 Hz, 1 H, H_1a_′), 3.71–3.77 (m, 1 H, H_4_′), 3.54–3.46 (m, 1 H, H_5_′), 3.14 (s, 1 H, NCH_3_), 3.05 (s, 1 H, NCH_3_), 2.57–2.51 (m, 1 H, H_3b_′), 1.99–2.09 ppm (m, 4 H, CH_3_, H_3a_′); ^13^C NMR (75 MHz, CDCl_3_): *δ*=172.4 (C4), 158.8 (C=N), 157.9 (C2), 137.2 (Ar), 136.3 (C6), 129.2, 128.4, 126.1 (Ar), 117.7 (C5), 102.1 (CHPh), 74.3, 73.7 (C4′, C5′), 69.1, 69.0 (C6′, C1′), 53.2 (C2′), 41.3, 35.2 (NCH_3_), 33.7 (C3′), 14.6 ppm (CH_3_); HRMS (ESI+): calcd for C_21_H_26_N_4_O_4_: 399.2026 [*M*+H]^+^; found: 399.2025.

### 1′,5′-Anhydro-2′,3′-dideoxy-2′-[6-(dimethylamino)methyl-idene-5-methyl-isocytosin-1-yl)-d-arabino-hexitol (23)

A solution of compound **22** (470 mg, 1.13 mmol) in acetic acid/water (20 mL, 6:4) was stirred at 60 °C for 4 h. The mixture was evaporated under reduced pressure and co-evaporated with toluene. The crude material was purified by flash column chromatography (CH_2_Cl_2_/MeOH, 9:1) to give **23** (903 mg, 93 %) as a white solid. UV (MeOH): *λ*_max_=247, 282 nm; ^1^H NMR (500 MHz, CD_3_OD): *δ*=8.65 (s, 1 H, CH=N), 8.19 (s, 1 H, H_6_), 5.12 (br s, 1 H, H_2_′), 4.24 (d, *J*=13.6 Hz, 1 H, H_1b_′), 3.92 (dd, *J*=13.6, 3.5 Hz, 1 H, H_1a_′), 3.84 (dd, *J*=12.0, 9.6 Hz, 1 H, H_6b_′), 3.77 (dd, *J*=12.0, 5.0 Hz, 1 H, H_6a_′), 3.70–3.66 (m, 1 H, H_4_′), 3.32–3.22 (m, 1 H, H_5_′), 3.21 (s, 3 H, NCH_3_), 3.13 (s, 3 H, NCH_3_), 2.37 (d, *J*=13.6 Hz, 1 H, H_3b_′), 1.94 (s, 3 H, CH_3_), 1.84–1.92 ppm (m, 1 H, H_3a_′); ^13^C NMR (125 MHz, CD_3_OD): *δ*=174.7 (C4), 160.1 (C=N), 159.9 (C2), 140.2 (C6), 117.2 (C5), 83.9 (C5′), 69.0 (C1′), 62.4 (C4′), 62.3 (C6′), 54.9 (C2′), 41.5 (NCH_3_), 37.2 (NCH_3_), 35.4 (C3′), 14.1 ppm (CH_3_); HRMS (ESI+): calcd for C_14_H_22_N_4_O_4_: 311.1713 [*M*+H]^+^; found: 311.1714.

### 1′,5′-Anhydro-2′,3′-dideoxy-2′-[6-(dimethylamino)methylidene-5-methyl-isocytosin-1-yl]-6′-*O*-dimethoxytrityl d-arabino-hexitol (24)

Reaction of compound **23** (300 mg, 0.96 mmol) with 4,4′-dimethoxytrityl chloride (360 mg, 1.06 mmol) in dry pyridine (20 mL) was carried out as described for compound **12** and purified by flash column chromatography (CH_2_Cl_2_/MeOH/TEA, 97:3:1) to give **24** (520 mg, 88 %) as a white foam. UV (MeOH): *λ*_max_=236, 282 nm; ^1^H NMR (500 MHz, [D_6_]DMSO): *δ*=8.82 (s, 1 H, CH=N), 8.02 (s, 1 H, H_6_), 7.45–7.27 (m, 9 H, Ar), 6.81–6.86 (m, 4 H, Ar), 5.05 (br s, 1 H, H_2_′), 4.23 (d, *J*=13.5 Hz, 1 H, H_1b_′), 3.95 (m, 1 H, H_4_′), 3.85 (dd, *J*=13.5, 3.5 Hz, 1 H, H_1a_′), 3.78 (s, 3 H, OCH_3_), 3.77 (s, 3 H, OCH_3_), 3.47 (dd, *J*=10.2, 4.0 Hz, 1 H, H_6b_′), 3.41 (dd, *J*=10.2, 4.0 Hz, 1 H, H_6a_′), 3.36–3.29 (m, 1 H, H_5_′), 3.13 (s, 3 H, NCH_3_), 3.06 (s, 3 H, NCH_3_), 2.47–2.39 (m, 1 H, H_3b_′), 1.95 (s, 3 H, CH_3_), 1.86–1.77 ppm (m, 1 H, H_3a_′); ^13^C NMR (125 MHz, [D_6_]DMSO): *δ*=172.3 (C4), 158.8, 158.6, 157.9 (Ar, C=N, C2), 144.6 (C6), 136.6, 135.8, 135.7, 130.0, 128.0, 127.0 (Ar), 117.5 (C5), 113.3 (Ar), 86.5 (CPh_3_), 80.9 (C5′), 68.6 (C1′), 63.5, 63.3 (C4′, C6′), 55.3 (OCH_3_), 52.7 (C2′), 41.2, 36.1 (NCH_3_), 35.1 (C3′), 14.6 ppm (CH_3_); HRMS (ESI+): calcd for C_35_H_40_N_4_O_6_: 613.3020 [*M*+H]^+^; found: 613.3027.

### 1′,5′-Anhydro-2′,3′-dideoxy-2′-[6-(dimethylamino)methyl-idene-5-methyl-isocytosin-1-yl)-6′-*O*-dimethoxytrityl-d-arabino-hexitol-4′-(2-cyanoethyl-*N*,*N*-diisopropyl)phosphoramidite (25)

Reaction of **24** (300 mg, 0.96 mmol) with 1 m cyanoethyl-bis(*N*,*N*-diisopropylamino) phosphine (0.97 mL, 0.97 mmol) and 1*H*-tetrazole (1.20 mL, 0.53 mmol) in dry CH_2_Cl_2_ (15 mL) was carried out as described for **13** and purified by flash column chromatography (*n*-hexane/acetone/TEA, 60:40:1) to give diastereomeric mixture **25** (325 mg, 81 %) as a white foam. ^31^P NMR (121 MHz, CDCl_3_): *δ*=148.4, 148.1 ppm; ^1^H NMR (500 MHz, CDCl_3_): *δ*=8.85 (s, 1 H, CH=N), 8.07 (s, 1 H, H_6_), 7.49–7.20 (m, 9 H, Ar), 6.85–6.80 (m, 4 H, Ar), 5.06 (s, 1 H, H_2_′), 4.27 (d, *J*=13.1 Hz, 1 H, H_1b_′), 4.17–4.10 (m, 1 H, H_4_′), 3.95–3.90 (m, 1 H, H_1a_′), 3.79 (s, 3 H, OCH_3_), 3.78 (s, 3 H, OCH_3_), 3.55–3.23 (m, 7 H, H_6a_′, H_6b_′, H_5_′, OCH_2_, 2 NCH), 3.14 (s, 3 H, NCH_3_), 3.05 (s, 3 H, NCH_3_), 2.50 (br d, *J*=14.3 Hz, 1 H, H_3b_′), 2.27 (t, *J*=6.2 Hz, 2 H, CH_2_CN), 1.93–1.86 (m, 1 H, H_3a_′), 1.82 (s, 3 H, CH_3_), 0.90–1.08 ppm (m, 12 H, 4 CH_3_); HRMS (ESI+): calcd for C_44_H_57_N_6_O_7_P: 813.4098 [*M*+H]^+^; found: 813.4091.

### 1′,5′-Anhydro-2′,3′-dideoxy-2′-(isoguanin-1-yl)-d-arabino-hexitol-6′-triphosphate-tetrabutylammonium salt (26)

Phosphoryl chloride (20 μL, 0.22 mmol) was added to an ice-cold solution of protected h-isoC^Me^ **11** (60 mg, 0.11 mmol) in trimethylphosphate (TMP) (1.0 mL) and the solution was stirred at 0 °C for 5 h. Tributylamine (300 μL, 1.6 mmol) and tetrabutylammonium pyrophosphate solution (0.5 m in DMF, 1.1 mmol) was added simultaneously, and the solution stirred for a further 30 min. The reaction was then quenched by the addition of 0.5 m triethylammonium bicarbonate (TEAB) buffer (10 mL), and stored at 4 °C overnight. The solvent was evaporated and the residue was treated with 25 % ammonia (4 mL). The solution was evaporated to dryness and re-dissolved in water (5 mL) and applied to a Sephadex A25 column in 0.1 m TEAB buffer. The column was eluted with a linear gradient of 0.1–1.0 m TEAB. Appropriate fractions were pooled and evaporated to dryness to give desired product. HPLC (Alltima 5μ C-18 reverse phase column 10×250 mm, buffer A, 0.1 m TEAB; buffer B, 0.1 m TEAB, 25 % MeCN. 0 % to 100 % buffer B over 60 min at 3 mL min^−1^) showed the product to be pure. ^31^P NMR (121 MHz, D_2_O): *δ*=−10.21 (1 P, d), −10.72 (1 P,d), −23.49 ppm (1 P, t); HRMS (ESI): calcd for C_11_H_18_N_5_O_13_P_3_: 520.0041 [*M*−H]^−^; found: *m*/*z*: 520.0043.

### 1′,5′-Anhydro-2′,3′-dideoxy-5′-acetoxy-2′-[6-(Dimethylamino)methylidene-5-methyl-isocytosin-1-yl]-d-arabino-hexitol (27)

Acetic anhydride (91.9 mmL, 972 μmol) was added dropwise, over one min, at room temperature to a THF (5 mL) solution of compound **24** (149 mg, 243 μmol), 4-methylaminopyridine (11.8 mg, 97.27 μmol), and diisopropylethylamine (251 mg, 1.95 μmol). After stirring for 20 min, ethyl acetate (20 mL) was added and the solution was washed with saturated brine (3×5 mL). The organic phase was dried (anhydrous Na_2_SO_4_) and solvent removed under vacuum to yield white foam, which was used directly without further purification for the next step. A 2.5 % solution of dichloroacetic acid (3 mL ) was added at 0 °C to a solution of acetylated h-isoC^Me^ in 10 mL of CH_2_Cl_2_. The reaction mixture was stirred at room temperature for 10 min. After completion of the reaction (monitored by TLC), the reaction mixture was diluted with CH_2_Cl_2_ (20 mL), and the organic layer was then washed 2 times with 5 mL of saturated aqueous NaHCO_3_. The organic layer was dried over Na_2_SO_4_ and concentrated in vacuum, and the residue was purified by flash column chromatography (CH_2_Cl_2_/MeOH, 95:5) to give **27** (50 mg, 59 %) as a white solid. ^1^H NMR (500 MHz, CD_3_OD): *δ*=8.67 (s, 1 H, CH=N), 8.15 (s, 1 H, H_6_), 5.12 (br s, 1 H, H_2_′), 4.37 (dd, *J*=11.9, 2.1 Hz, 1 H, H_6b_′), 4.32 (dd, *J*=11.9, 4.8 Hz, 1 H, H_6a_′), 4.24 (d, *J*=13.5 Hz, 1 H, H_1b_′), 3.93 (dd, *J*=13.5, 3.4 Hz, 1 H, H_1a_′), 3.71–3.63 (m, 1 H, H_4_′), 3.46–3.42 (m, 1 H, H_5_′), 3.22 (s, 3 H, NCH_3_), 3.13 (s, 3 H, NCH_3_), 2.43–2.37 (m, 1 H, H_2b_′), 2.07 (s, 3 H, CH_3_), 1.96 (s, 3 H, CH_3_), 1.85–1.92 ppm (m, 1 H, H_2a_′); ^13^C NMR (125 MHz, CD_3_OD): *δ*=173.2, 171.1 (C4, C=O), 158.7 (C=N), 158.4 (C2), 138.5 (C6), 115.6 (C5), 79.7 (C5′), 67.5 (C1′), 63.0(C6′), 60.9 (C4′), 53.4 (C2′), 40.0 (NCH_3_), 35.6 (NCH_3_), 33.9 (C3′), 19.2 (CH_3_), 12.8 ppm (CH_3_); HRMS (ESI+): calcd for C_16_H_24_N_4_O_5_: 353.1819 [*M*+H]^+^; found: 353.1819.

### 1′,5′-Anhydro-2′,3′-dideoxy-2′-(5-methylisocytosin-1-yl)-d-arabino-hexitol-6′-triphosphate-tetrabutylammonium salt (28)

Protected h-isoC^Me^ **27** (20 mg, 0.057 mmol) was dried overnight and dissolved in a mixture of anhydrous pyridine (0.6 mL) and dioxane (1 mL). Salicyl phosphorochloridite (14.0 mg, 0.068 mmol) was added as a solution in dioxane (0.3 mL) and the reaction mixture was stirred at 25 °C for 1 min under nitrogen. The solution became dark brown. A solution of tributylammonium pyrophosphate (41.3 mg, 0.091 mmol) in DMF (0.5 mL) and tributylamine (0.064 mL, 0.267 mmol) were added to the reaction mixture and stirred for 20 min. A solution of iodine (0.68 mL, 0.068 mmol) in pyridine/H_2_O (98/2) was added and the resulting solution was stirred for 20 min, then quenched with 5 % Na_2_SO_3_ until the red colour disappeared. The solvent was evaporated and the residue was treated with 25 % ammonia (4 mL). The solution was evaporated to dryness, the residue was re-dissolved in water (5 mL) and purified as described for h-isoGTP above to give h-isoC^Me^TP. ^31^P NMR (121 MHz, D_2_O): *δ*=−10.85 (2 P, br s), −23.24 ppm (1 P, t); HRMS (ESI): calcd for C_11_H_20_N_3_O_13_P_3_: 494.0136 [*M*−H]^−^; found: 494.0141.

### Oligonucleotide synthesis

Oligonucleotide assemblywas performed with an Expedite DNA synthesiser (Applied Biosystems) by using the phosphoramidite approach. The oligomers were deprotected and cleaved from the solid support by treatment with aqueous ammonia (30 %) for 2 h. After gel filtration on a NAP-25 column (Sephadex G25-DNA grade; Pharmacia) with water as the eluent, the crude mixture was analysed by using a Mono-Q HR 5/5 anion exchange column, after which purification was achieved by using a Mono-Q HR 10/100 GL column (Pharmacia) with the following gradient system: *A*=10 mm NaClO_4_ in 15 % CH_3_CN, pH 7.4, *B*=600 mm NaClO_4_ in 15 % CH_3_CN, pH 7.4. The low-pressure liquid chromatography system consisted of a Merck–Hitachi l-6200 A intelligent pump, a Mono-Q HR 10/100 GL column (Pharmacia), an Uvicord SII 2138 UV detector (Pharmacia-LKB) and a recorder. The product-containing fraction was desalted on a NAP-25 column and lyophilised (Table 8).

**Table 8:** MS analysis of oligonucleotides

Oligonucleotide sequence	Mass calcd	Mass found
GGT AGC A(d-isoG)C GGT G	4053.7	4053.4
GTC CTT T GTC GAT ACT G (d-isoG) GT CAA-	7042.2	7042.5
GTC CTT T GTC GAT ACT G (d-isoG)^3^ GT CAA	7703.6	7703.6
GGT AGC A(h-isoG)C GGT G	4067.7	4067.6
GTC CTT T GTC GAT ACT G (h-isoG)GT CAA	7056.2	7056.5
GTC CTT T GTC GAT ACT G (h-isoG)^3^GT CAA	7745.7	7744.8
CCA TCG T(d-isoC^Me^)G CCA C	3867.7	3867.8
GTC CTT T GTC GAT ACT G (d-isoC^Me^)GT CAA	7016.2	7016.6
GTC CTT T GTC GAT ACT G (d-isoC^Me^)^3^GT CAA	7622.3	7622.8
CCA TCG T(h-isoC^Me^)G CCA C	3881.8	3881.7
GTC CTT T GTC GAT ACT G (h-isoC^Me^)GT CAA	7030.2	7030.3
GTC CTT T GTC GAT ACT G (h-isoC^Me^)^3^GT CAA	7664.4	7664.5

### UV melting experiments

Oligomers were dissolved in a buffer solution containing NaCl (0.1 m), potassium phosphate (0.02 m, pH 7.5) and EDTA (0.1 mm). The concentration was determined by measuring the absorbance in Milli-Q water at 260 nm at 80 °C, and by assuming that hexitol nucleosides have the same extinction coefficients per base moiety in the denatured state as the natural nucleosides (A*, *ε*=15 000; T*, *ε*=8500). The concentration for each strand was 4 μm in all experiments. Melting curves were determined with a Varian Cary 100 BIO spectrophotometer. Cuvettes were maintained at constant temperature by water circulation through the cuvette holder. The temperature of the solution was measured with a thermistor that was directly immersed in the cuvette. Temperature control and data acquisition were carried out automatically with an IBM-compatible computer by using Cary WinUV thermal application software. A quick heating and cooling cycle was carried out to allow proper annealing of both strands. The samples were then heated from 10 to 80 °C at a rate of 0.2 °C min^−1^, and were cooled again at the same speed. Melting temperatures were determined by plotting the first derivative of the absorbance as a function of temperature; data plotted were the average of two runs. Up and down curves in general showed identical *T*_m_ values.

### Incorporation experiments

All the natural triphosphates were purchased from GE Healthcare (HPLC purification) or TriLink Biotechnologies (HPLC purification). The natural oligonucleotides were purchased from IDT (in lyophilised form) and reconstituted with Milli-Q water to give 100 μm. Vent (*exo*-), Taq and KF (*exo*-) polymerases were purchased from New England Biolabs, Pfu (*exo*-) from Agilent Technologies, Tfi (*exo*-) from Life technologies, T7 sequenase from Affymetrix, T4(*exo*-) from Lucigen and Pol III α-subunit was expressed and purified as described.[32]

First, the primer (P1) was 5′- labeled with [^33^P]ATP (3000 Ci mmol^−1^, 10 mCi mL^−1^) (Perkin–Elmer) using T4 polynucleotide kinase (10 U/μL) (New England Biolabs), following the procedures suggested by the manufacturer. The unincorporated radionucleotides were removed using Illustra Microspin G-25 columns (GE Healthcare). The end-labelled primer was annealed to each template (primer: template molar ratio of 1:2) by heating the mixture at 70 °C for 4 min, followed by slowing cooling to room temperature over a period of 2 h. A series of 10 μL reactions were performed for all of the DNA polymerases. Incorporation experiments were duplicated and triplicates were obtained for the selectivity tests to ensure the accuracy of the results. Each batch reaction contained 125 nm of the primer:template, 1×reaction buffer (as provided by the supplier), 0.08 U μL^−1^ of TIPP, 0.08 U μL^−1^ of the DNA polymerases except 1.6 U μL^−1^ for Pol III α-subunit and varying nucleoside triphosphate concentrations (d-NTP or h-NTP). The reaction mixtures were incubated at 75 °C for Vent (*exo*-), Pfu (*exo*-), Taq and Tfi (*exo*-), at 37 °C for KF (*exo*-), T4 (*exo*-) and T7 sequenase version and at 30 °C for Pol III α-subunit. 2.0 μL aliquots were removed and quenched (by addition of 5 μL loading dye) at the desired time points. Loading dye consists of: 90 % formamide, 0.05 % xylene cyanol, 0.05 % bromophenol blue and 50 mm EDTA. The samples were analysed by gel electrophoresis for 2–3 h at 2000 V on a 30 cm×40 cm×0.4 mm 20 % denaturing gel in the presence of 100 mm Tris-Borate and 2.5 mm EDTA buffer (pH 8.3). The bands corresponding to the enzymatic reaction products were visualised using the Optiquant Image Analysis software (Perkin–Elmer).

### In vivo assay of XNA-oligonucleotides

Oligomers were dissolved in Milli-Q water to 100 mm and diluted ten-fold before assay. Oligomers were tested inside a gapped heteroduplex vector generated through the enzymatic digestion and PCR assisted denaturation and hybridisation of the ampicillin resistance gene containing pAK1 and pAK2 plasmids.[24] The form of the plasmids are described elsewhere in detail.[23, 24] Here, a mix of equimolar (25 ng each) purified NheI and NsiI cut pAK1 and purified two-fold EcoRI cut and dephosphorylated pAK2 were diluted in 10 mm Tris-HCl (pH 7.5) with 100 mm NaCL. The mixture was denatured at 95 °C for 5 min before cooling to ambient temperature over 2 h, before subsequent dialysis with water through 0.05 μm nitrocellulose filters (Millipore) for 30 min. Each oligomer for testing (0.02 pmol), as well as a positive (CTAGCGCCGTGCCATGCA) and negative (CTAGCGCCGCATGCA) oligomer control, was added to the dialysed heteroduplex mixture in 1× DNA ligase T4 reaction buffer (NEB) to 20 μL each. The mixture was denatured at 85 °C as before. Ligation was performed by adding 1 mm ATP and 5 U T4 DNA Ligase (NEB) to the samples before overnight incubation at 16 °C. Ligated mixtures were dialysed as before, and transformed by electroporation into a competent strain of *E. coli* K12 (Δ*thyA*:*aadA*). Incubation of the electroporated mixture was performed at 37 °C for 1 hour, before plating 100 μL of sub-dilutions of the mixture onto Muller-Hinton (MH) media containing 100 μg mL^−1^ ampicillin (10^−1^ and 10^−2^ dilutions) and onto the same media supplemented with 0.3 mm thymidine (10^−3^ and 10^−4^ dilutions).

### Data analysis and sequencing

The total number of thymidine–prototrophic and of ampicillin resistant colonies was calculated from the plate counts, and the ratio (former/latter) was recorded. Positive thymidine-prototrophic colonies were picked and restreaked twice overnight to MH ampicillin (100 μg mL^−1^), before overnight growth in 2 mL liquid cultures of the same medium. Plasmids were prepared by Miniprep (Qiagen) and the plasmids sequenced using an inter-*thyA* oligo (AACAGTGGCGCGCCTGG).

## Supporting Information

As a service to our authors and readers, this journal provides supporting information supplied by the authors. Such materials are peer reviewed and may be re-organized for online delivery, but are not copy-edited or typeset. Technical support issues arising from supporting information (other than missing files) should be addressed to the authors.

miscellaneous_information
