# Supplementary material for: Isoguanine and 5-Methyl-Isocytosine Bases, In Vitro and In Vivo
Source: Chemistry. 2015 Feb 13;21(13):5009–22. doi: 10.1002/chem.201406392 (PMC4531829; doi:10.1002/chem.201406392)
Supplement: Supplementary file 1 — miscellaneous_information [file chem0021-5009-sd1.pdf]

CHEMISTRY
A European Journal

Supporting Information

Isoguanine and 5-Methyl-Isocytosine Bases, In Vitro and In Vivo

Omprakash Bande,[a] Rania Abu El Asrar,[a] Darren Braddick,[b] Shrinivas Dumbre,[a] Valérie Pezo,[b, c] Guy Schepers,[a] Vitor B. Pinheiro,[d] Eveline Lescrinier,[a] Philipp Holliger,[e] Philippe Marlière,[b] and Piet Herdewijn*[a, b]

chem_201406392_sm_miscellaneous_information.pdf

Supporting Information

**Compound**	**NMR data**	**Page No**
**8**	^{1}H and ^{13}C,	S2
**9**	^{1}H and ^{13}C	S3
**2**	^{1}H, ^{13}C,	S4
**10**	^{1}H and ^{13}C	S5
**11**	^{1}H and ^{13}C	S6
**12**	^{1}H and ^{13}C	S7
**13**	^{31}P and ^{1}H	S8
**19**	^{1}H and ^{13}C,	S9
**20**	^{1}H, ^{13}C	S10
**21**	^{1}H and ^{13}C	S11
**22**	^{1}H and ^{13}C	S12
**23**	^{1}H and ^{13}C	S13
**24**	^{1}H and ^{13}C	S14
**25**	^{31}P and ^{1}H	S15
**26**	^{31}P and HRMS	S16
**27**	^{1}H and ^{13}C	S17
**28**	^{31}P and HRMS	S18

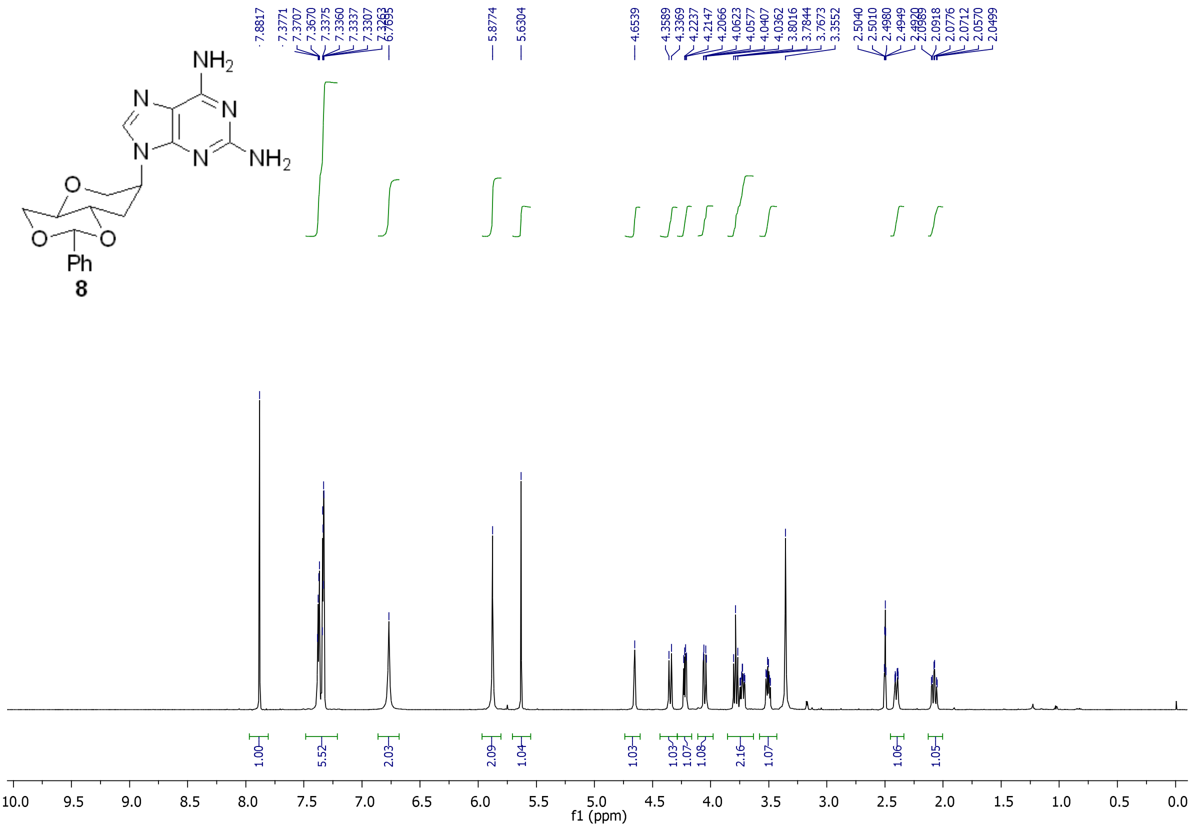
NH2
N
N
N
N
NH2
O
O
O
Ph
7.8817
7.3771
7.3707
7.3670
7.3375
7.3360
7.3337
7.3307
5.8774
5.6304
4.6539
4.3589
4.3369
4.2237
4.2147
4.2066
4.0623
4.0577
4.0407
4.0362
3.8016
3.7844
3.7673
3.3552
2.5040
2.5010
2.4980
2.4949
2.0918
2.0776
2.0712
2.0570
2.0499
1.00
5.52
2.03
2.09
1.04
1.03
1.03
1.07
1.08
2.16
1.07
1.06
1.05
10.0
9.5
9.0
8.5
8.0
7.5
7.0
6.5
6.0
5.5
5.0
4.5
4.0
3.5
3.0
2.5
2.0
1.5
1.0
0.5
0.0
f1 (ppm)

8

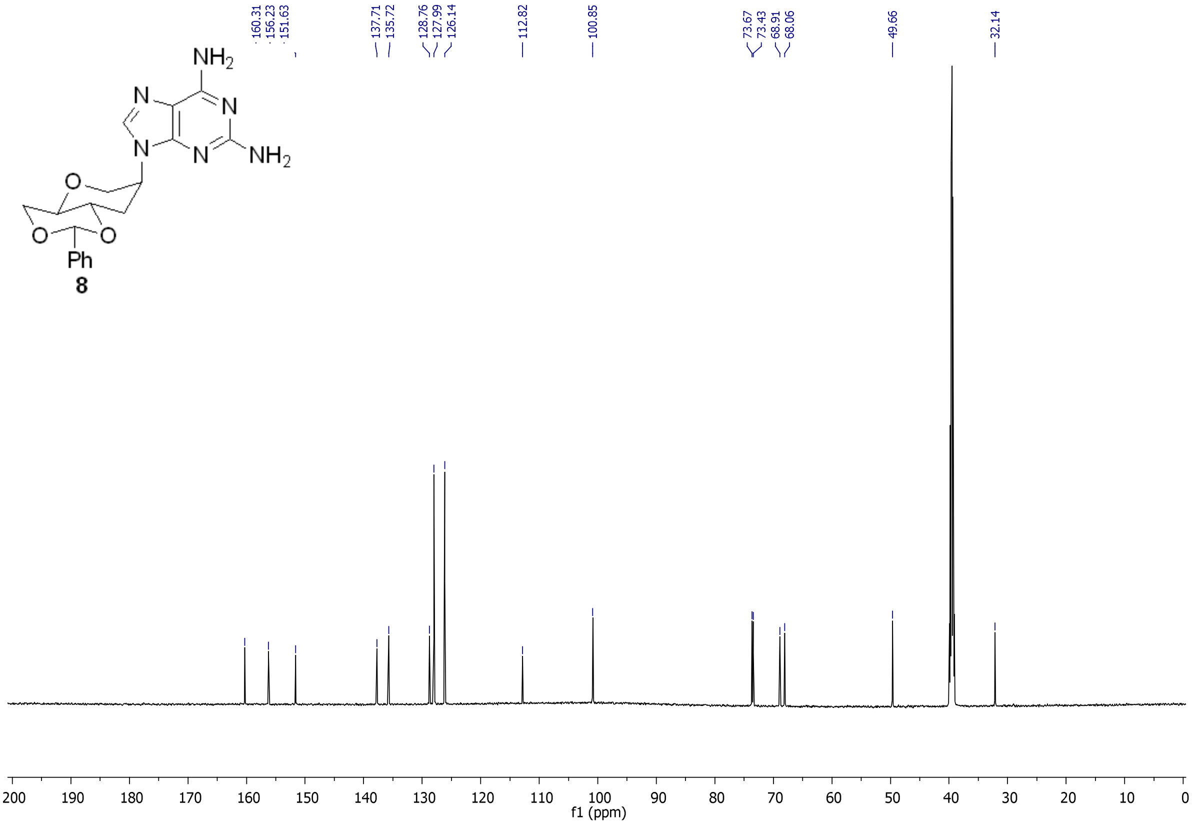
NH2
N
N
N
N
NH2
O
O
O
Ph
160.31
156.23
151.63
137.71
135.72
128.76
127.99
126.14
112.82
100.85
73.67
73.43
68.91
68.06
49.66
32.14
200
190
180
170
160
150
140
130
120
110
100
90
80
70
60
50
40
30
20
10
0
f1 (ppm)

8

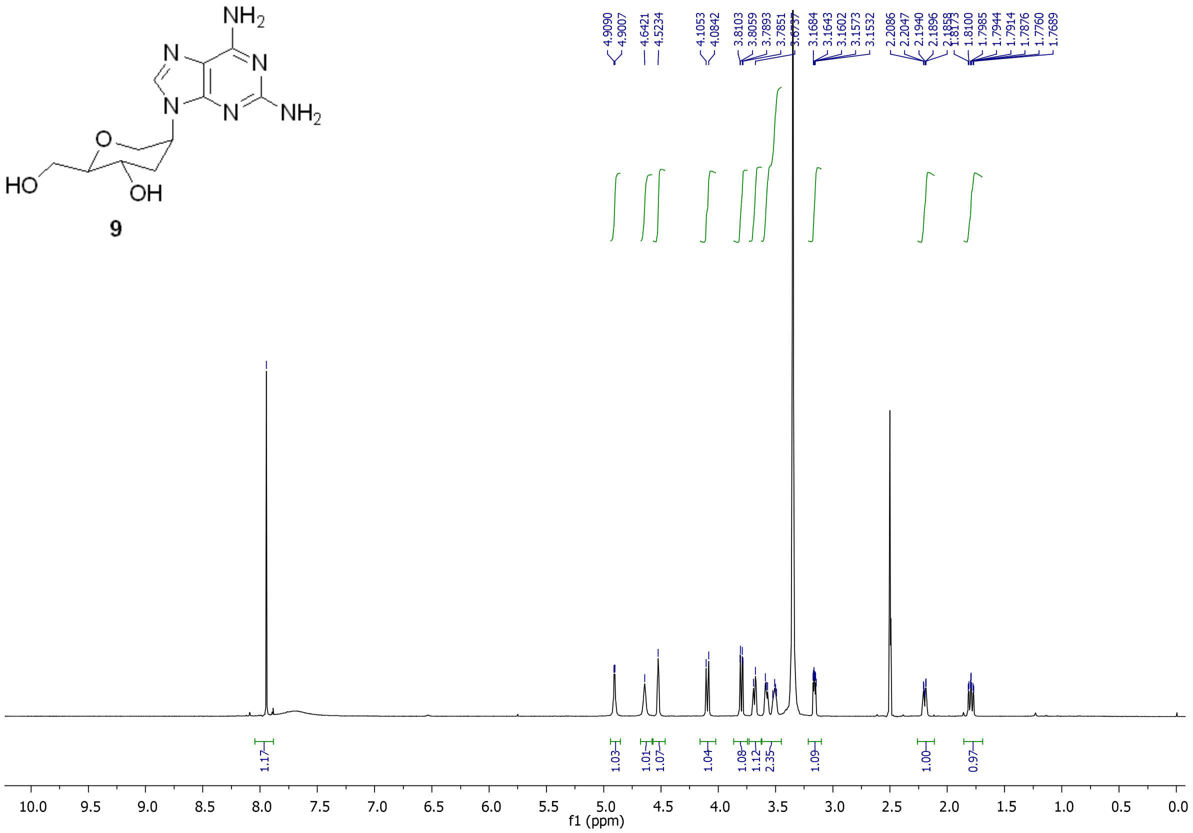
NH2
N
N
N
N
NH2
O
HO
OH
9
4.9090
4.9007
4.6421
4.5234
4.1053
4.0842
3.8103
3.8059
3.7893
3.7851
3.1684
3.1643
3.1602
3.1573
3.1532
2.2086
2.2047
2.1940
2.1896
1.8100
1.7985
1.7944
1.7914
1.7876
1.7760
1.7689
1.17
1.03
1.01
1.07
1.04
1.08
1.12
2.35
1.09
1.00
0.97
f1 (ppm)

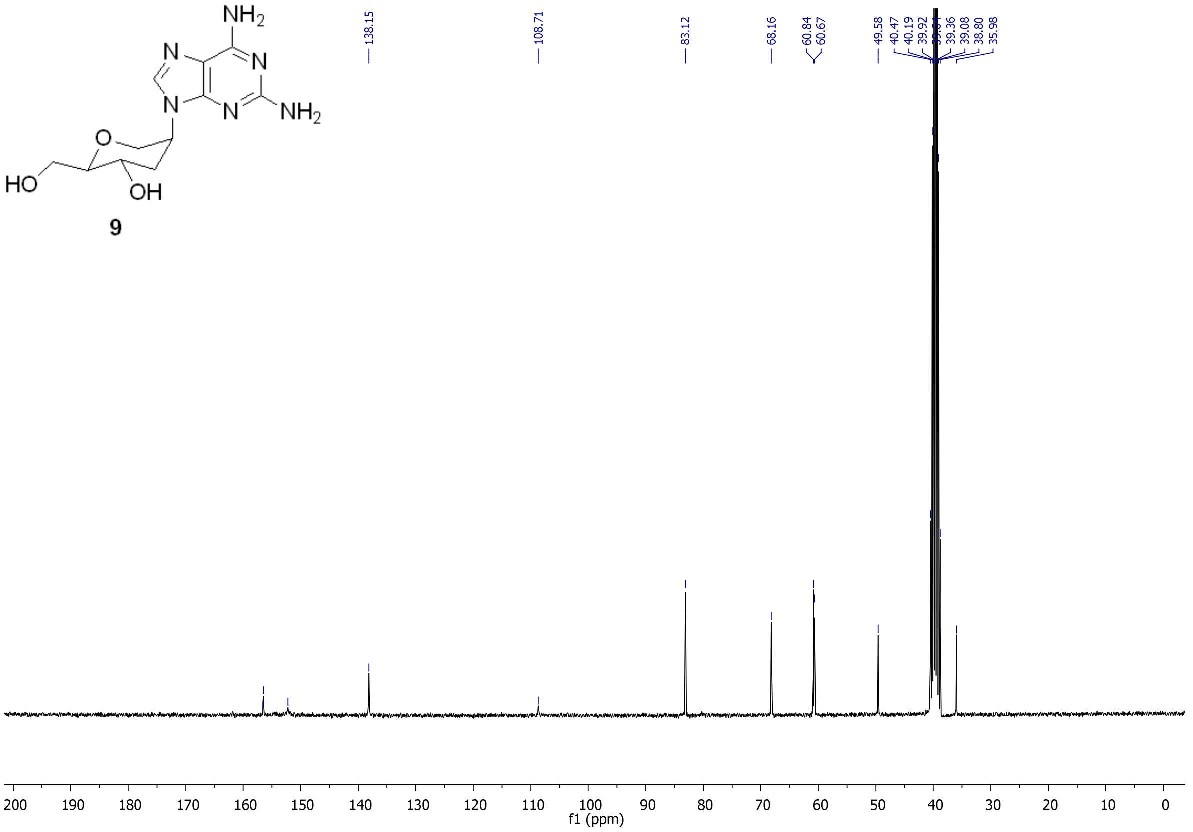
NH2
N
N
N
N
NH2
O
HO
OH
9
138.15
108.71
83.12
68.16
60.84
60.67
49.58
40.47
40.19
39.92
39.36
39.08
38.80
35.98
f1 (ppm)

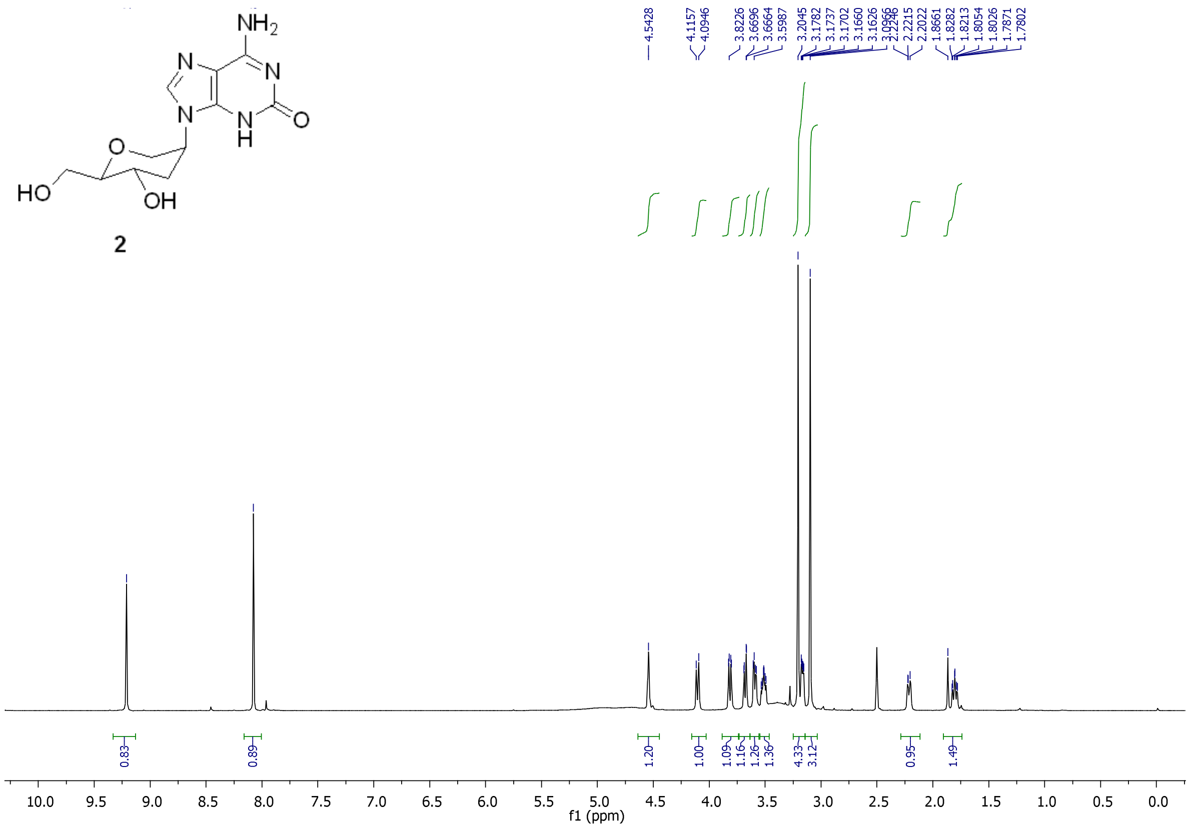
NH2
N
N
N
N
H
O
O
HO
OH
2
4.5428
4.1157
4.0946
3.8226
3.6696
3.6664
3.5987
3.2045
3.1782
3.1737
3.1702
3.1660
3.1626
3.0966
2.2246
2.2215
2.2022
1.8661
1.8282
1.8213
1.8054
1.8026
1.7871
1.7802
0.83
0.89
1.20
1.00
1.09
1.16
1.26
1.36
4.33
3.12
0.95
1.49
10.0
9.5
9.0
8.5
8.0
7.5
7.0
6.5
6.0
5.5
5.0
4.5
4.0
3.5
3.0
2.5
2.0
1.5
1.0
0.5
0.0
f1 (ppm)

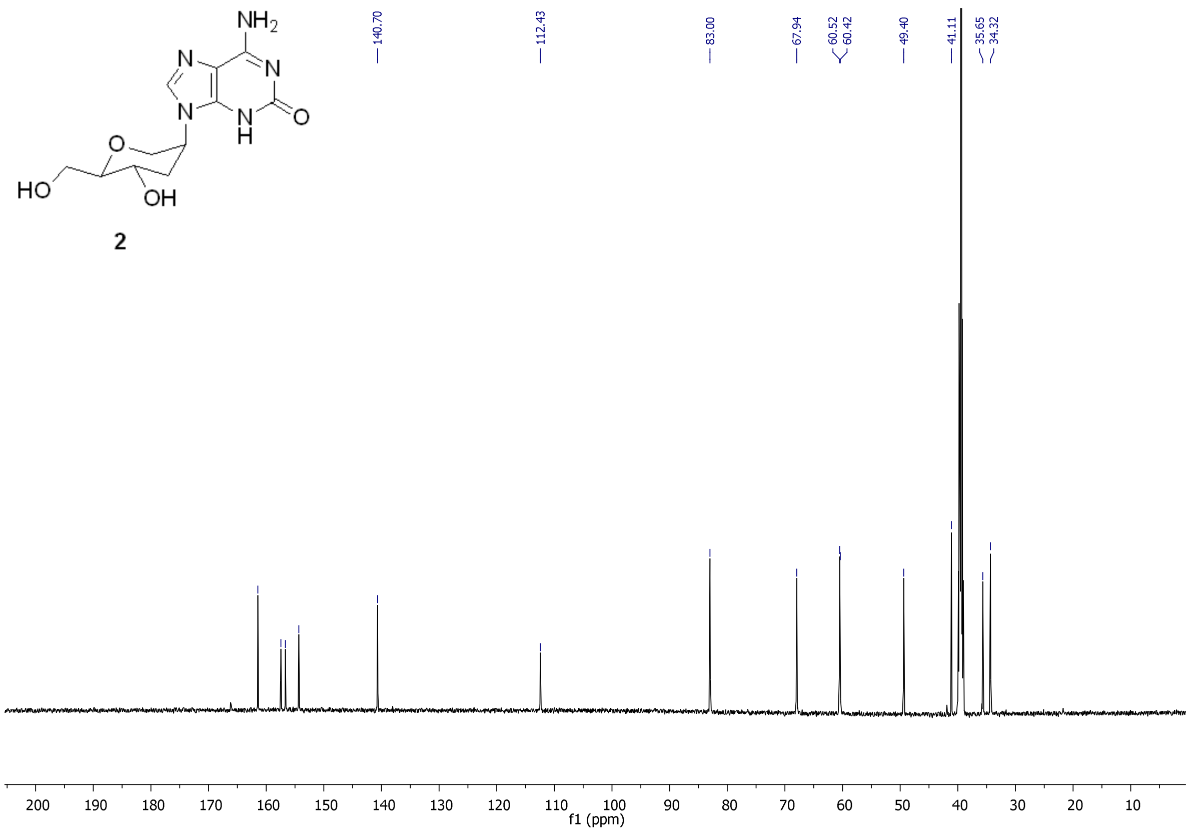
NH2
N
N
N
N
H
O
O
HO
OH
2
140.70
112.43
83.00
67.94
60.52
60.42
49.40
41.11
35.65
34.32
200
190
180
170
160
150
140
130
120
110
100
90
80
70
60
50
40
30
20
10
f1 (ppm)

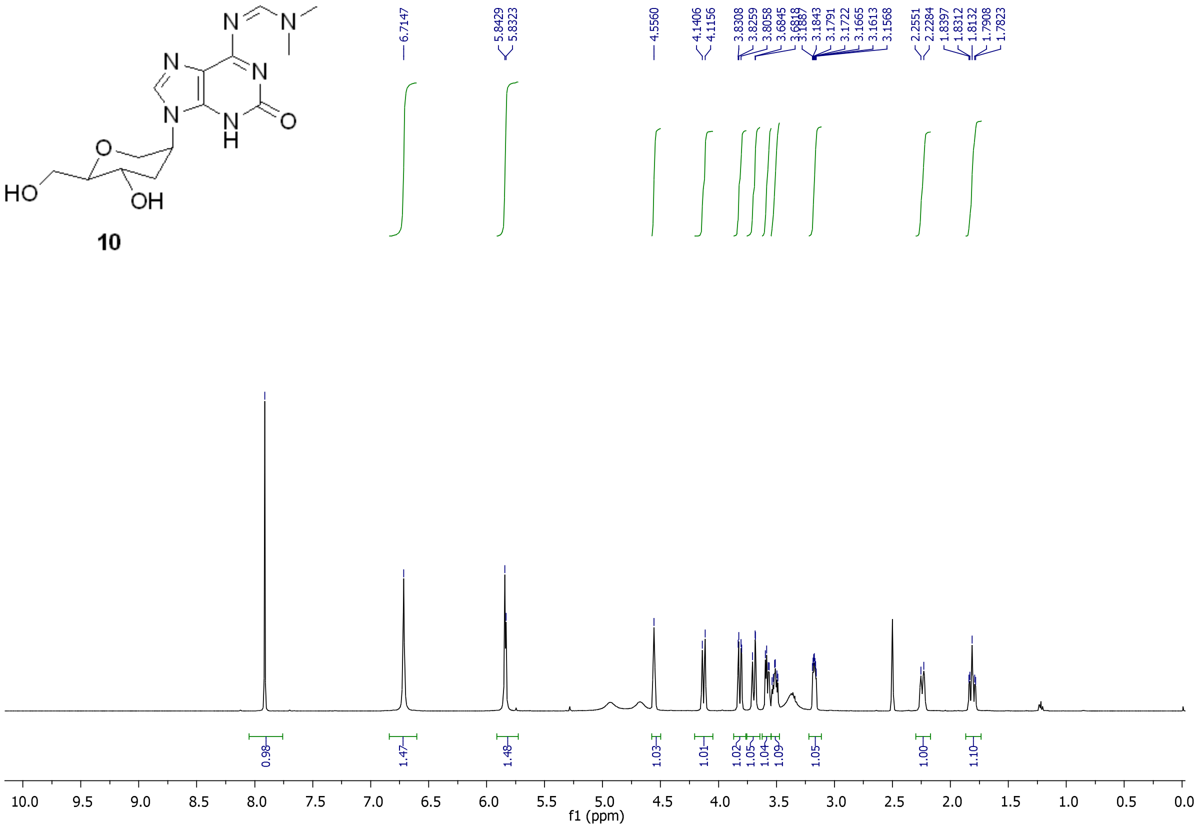
10
6.7147
5.8429
5.8323
4.5560
4.1406
4.1156
3.8308
3.8259
3.8058
3.6845
3.1843
3.1791
3.1722
3.1665
3.1613
3.1568
2.2551
2.2284
1.8397
1.8312
1.8132
1.7908
1.7823
0.98
1.47
1.48
1.03
1.01
1.02
1.05
1.04
1.09
1.05
1.00
1.10
f1 (ppm)

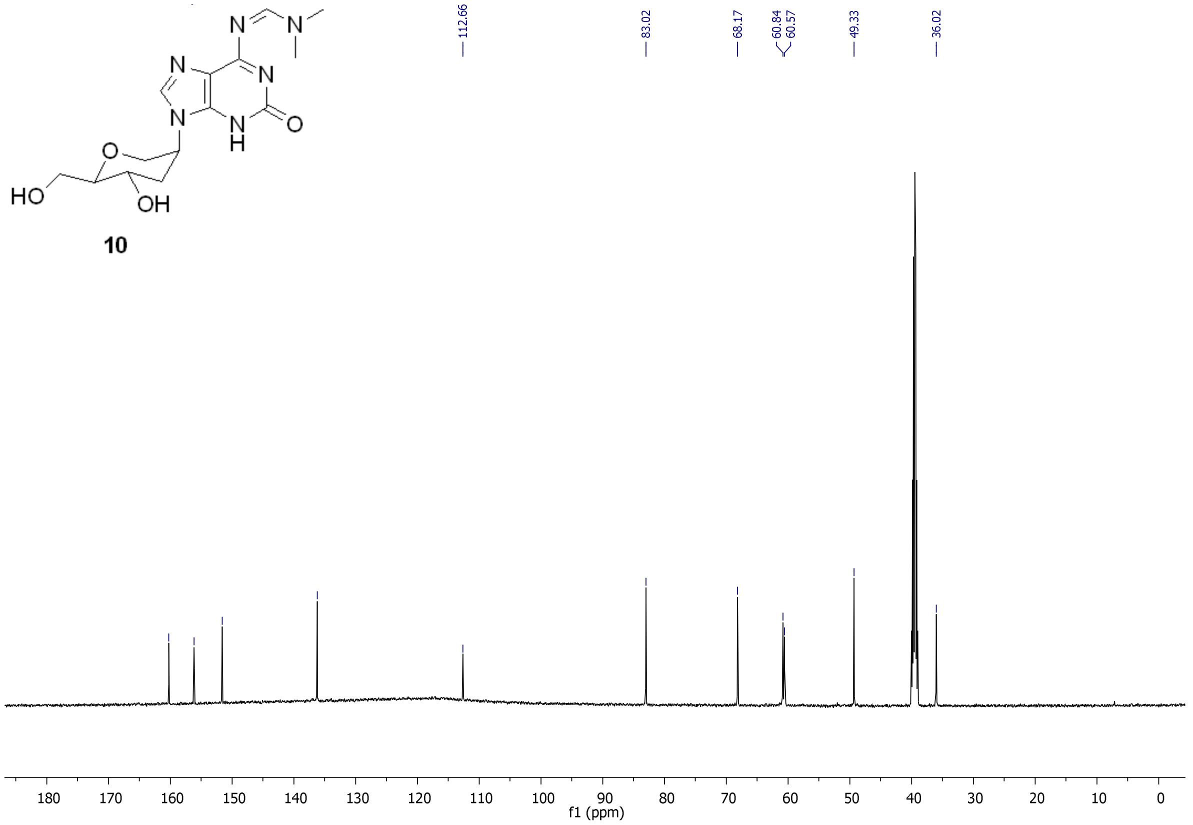
10
112.66
83.02
68.17
60.84
60.57
49.33
36.02
f1 (ppm)

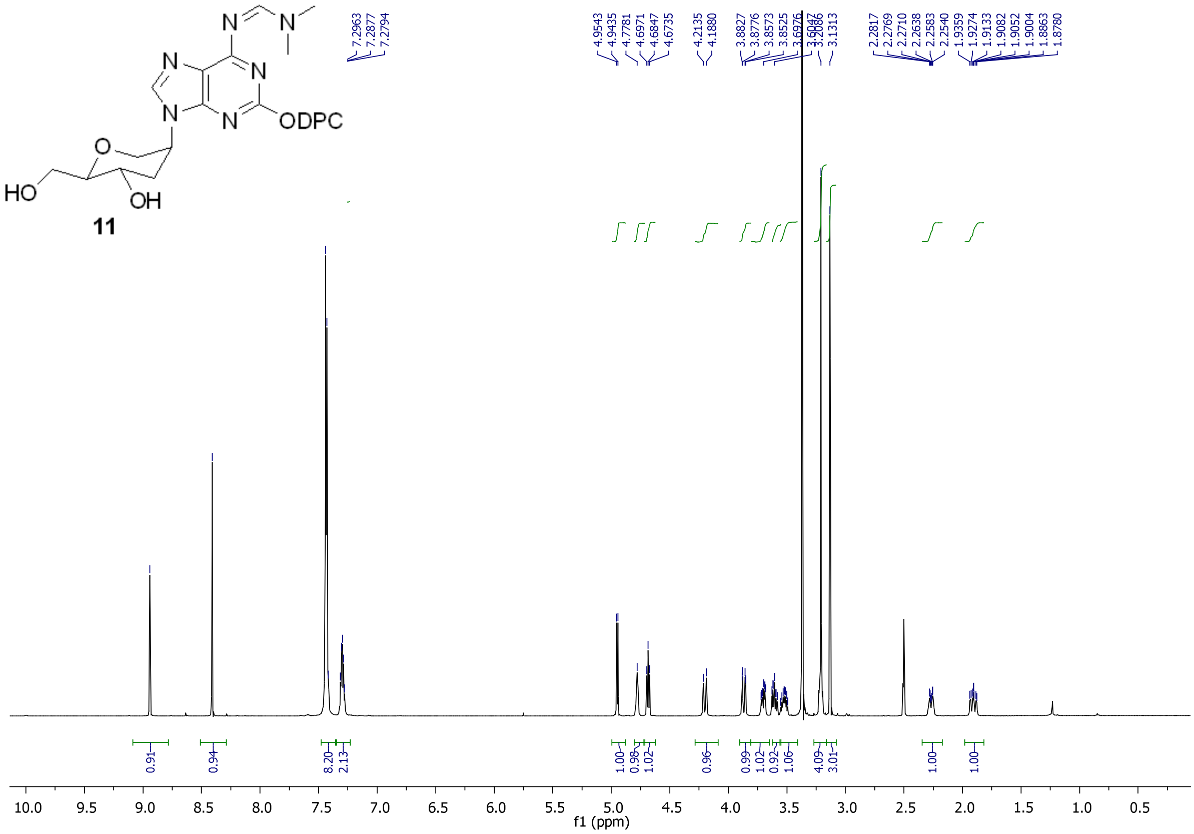
ODPC
HO
OH
11
f1 (ppm)

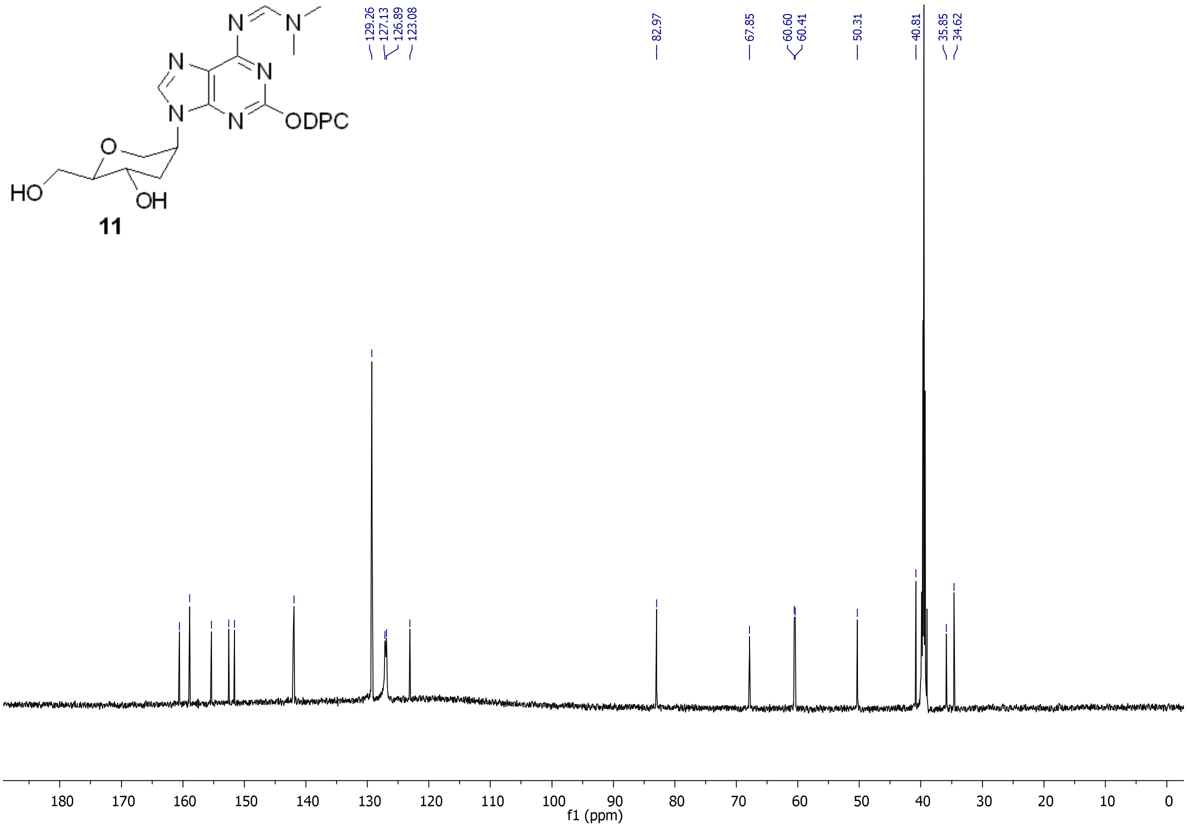
ODPC
HO
OH
11
f1 (ppm)

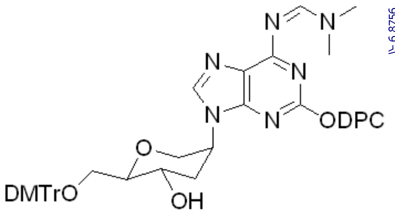

N
N
N
N
N
N
ODPC
O
DMTrO
OH

12

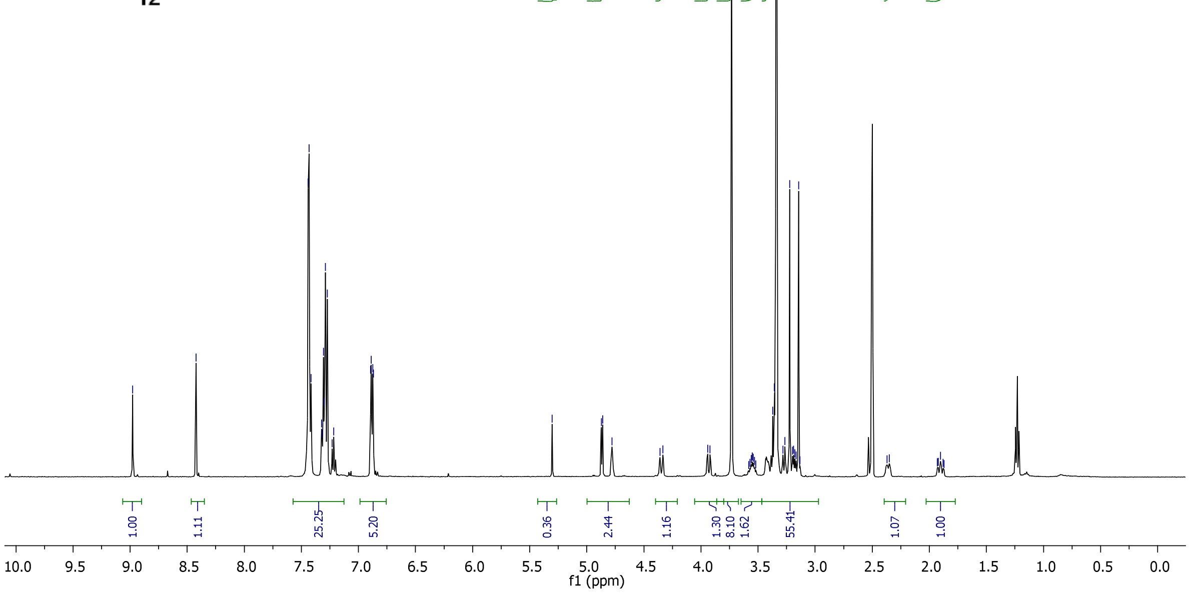

6.8756
6.8698
5.3029
4.8732
4.8616
4.7792
4.3595
4.3339
3.9420
3.9208
3.7338
3.7319
3.3708
3.3561
3.2814
3.2645
3.2236
3.1979
3.1879
3.1778
2.3513
1.9299
1.9218
1.9011
1.8802
1.8723
1.00
1.11
25.25
5.20
0.36
2.44
1.16
1.30
8.10
1.62
55.41
1.07
1.00
f1 (ppm)

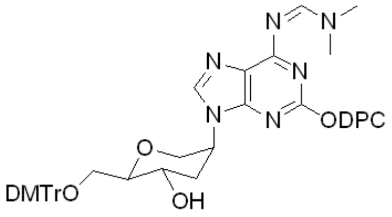

N
N
N
N
N
N
ODPC
O
DMTrO
OH

12

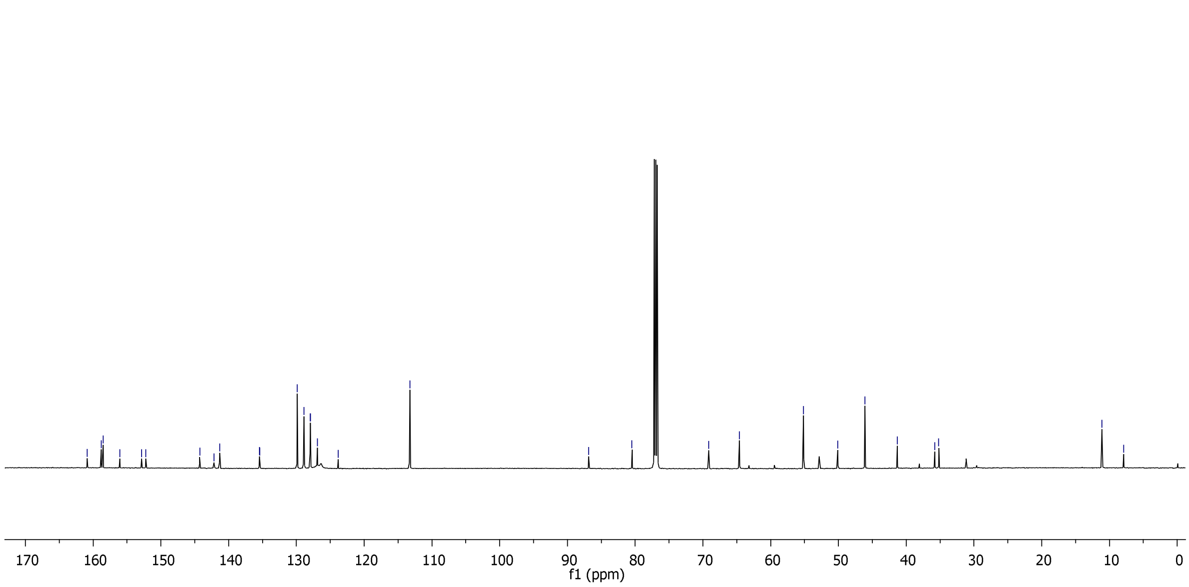

113.26
86.87
80.47
69.14
64.63
55.15
50.11
46.08
41.32
35.80
35.20
11.11
7.90
f1 (ppm)

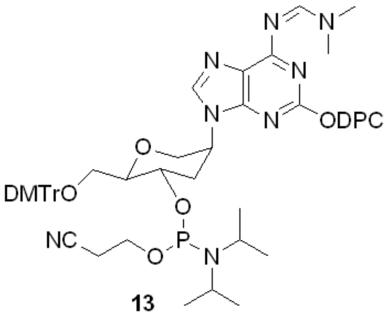

13

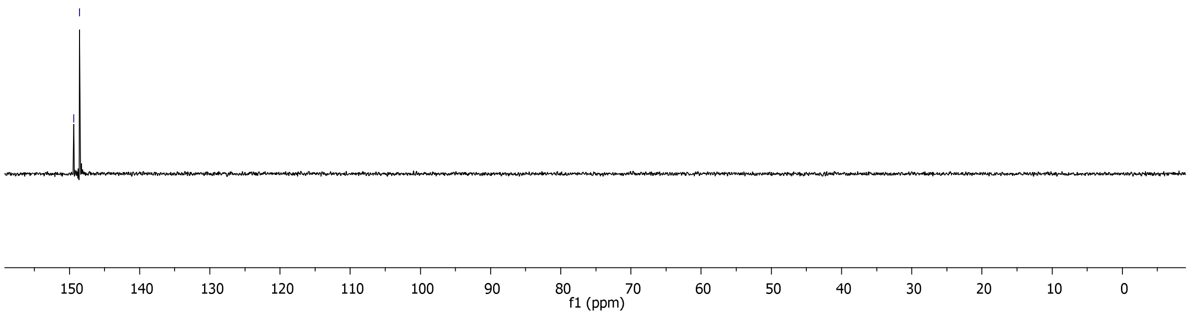

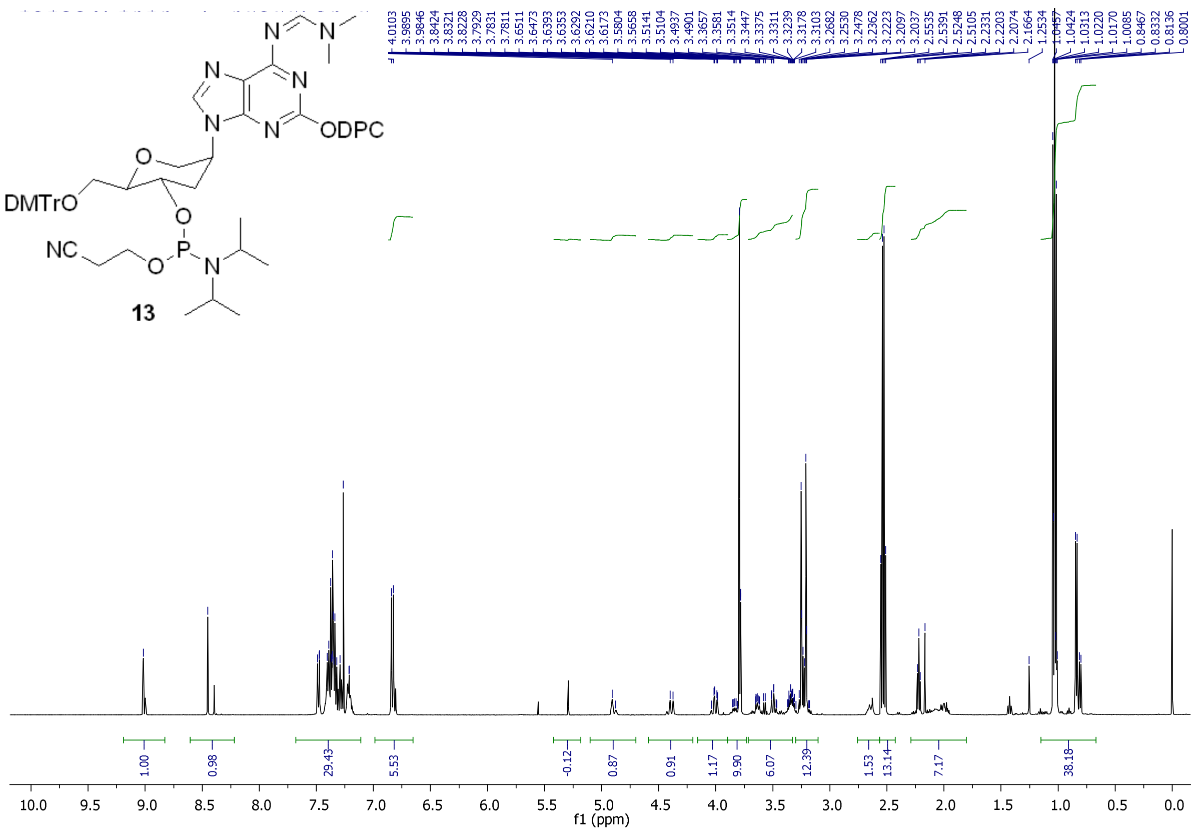

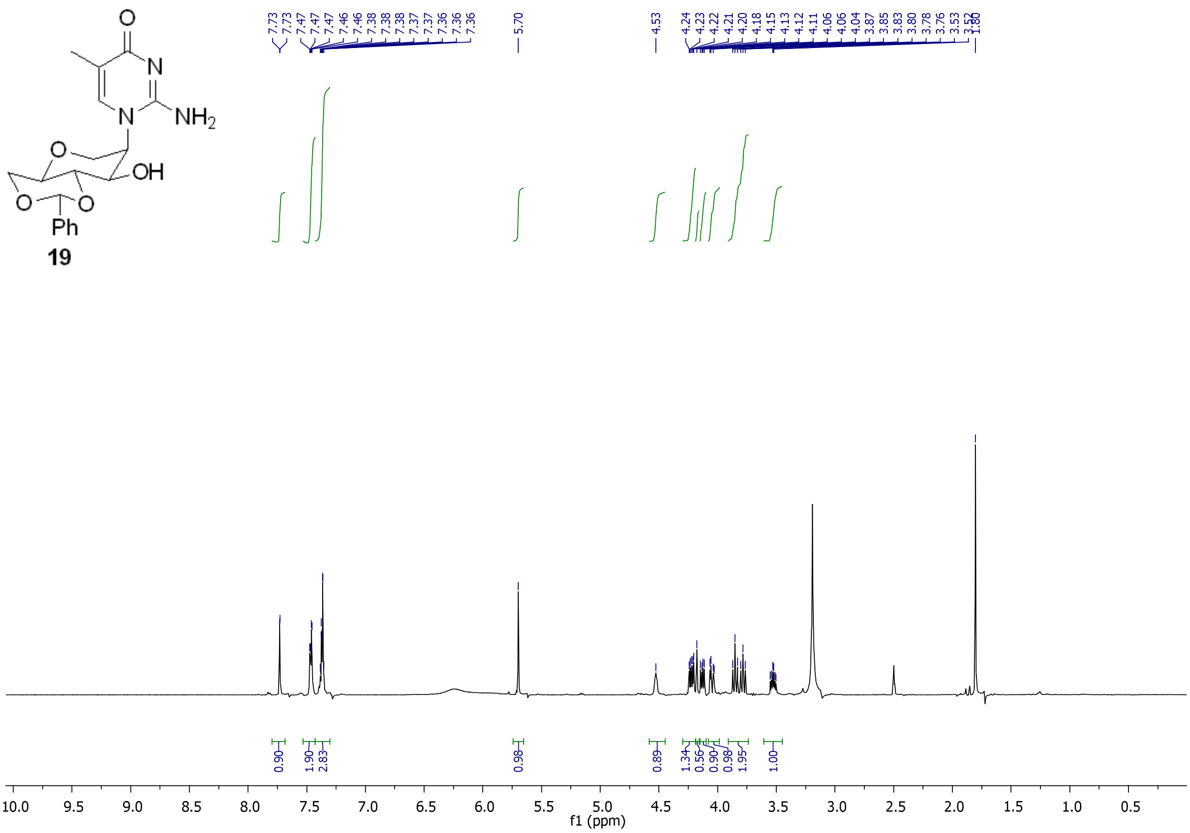
O
N
NH2
N
O
OH
O
O
Ph
19
7.73
7.73
7.47
7.47
7.47
7.46
7.38
7.38
7.38
7.37
7.37
7.36
7.36
7.36
5.70
4.53
4.24
4.23
4.22
4.21
4.20
4.18
4.15
4.13
4.12
4.11
4.06
4.04
3.87
3.85
3.83
3.80
3.78
3.76
3.53
3.52
1.80
0.90
1.90
2.83
0.98
0.89
1.34
0.56
0.90
0.98
1.95
1.00
10.0 9.5 9.0 8.5 8.0 7.5 7.0 6.5 6.0 5.5 5.0 4.5 4.0 3.5 3.0 2.5 2.0 1.5 1.0 0.5
f1 (ppm)

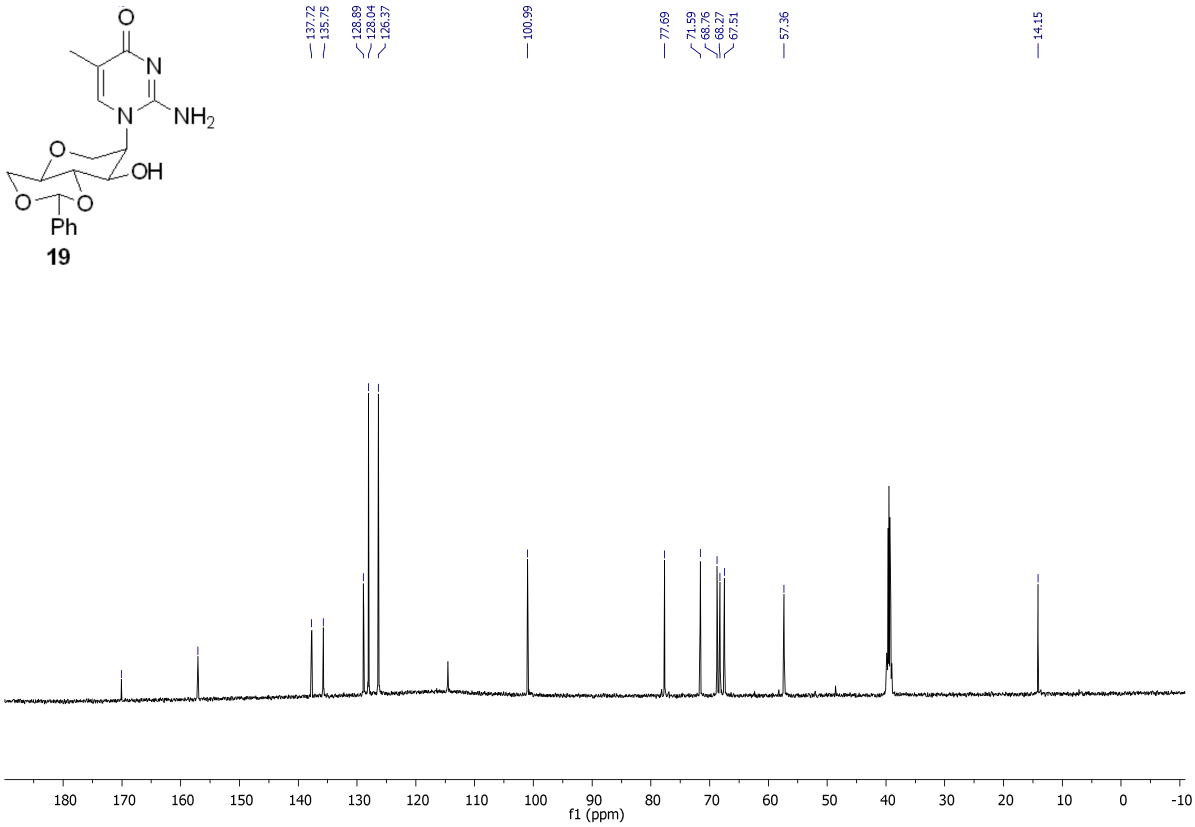
O
N
NH2
N
O
OH
O
O
Ph
19
137.72
135.75
128.89
128.04
126.37
100.99
77.69
71.59
68.76
68.27
67.51
57.36
14.15
180 170 160 150 140 130 120 110 100 90 80 70 60 50 40 30 20 10 0 -10
f1 (ppm)

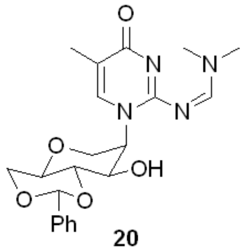

20

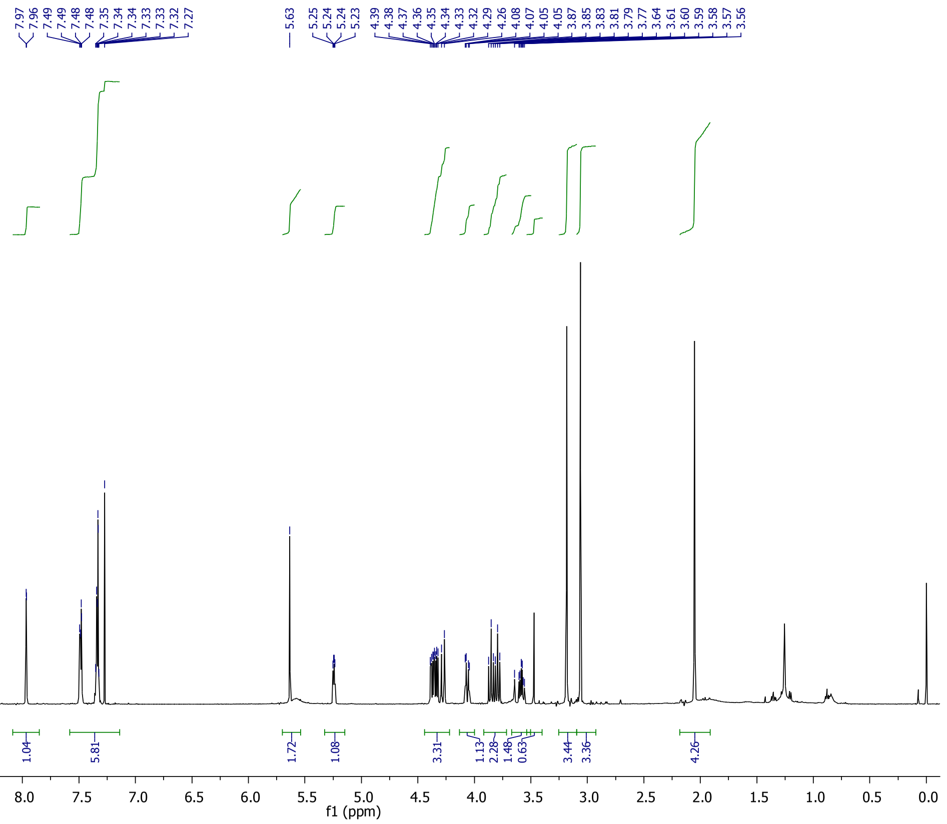

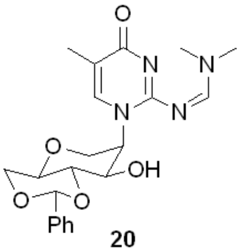

20

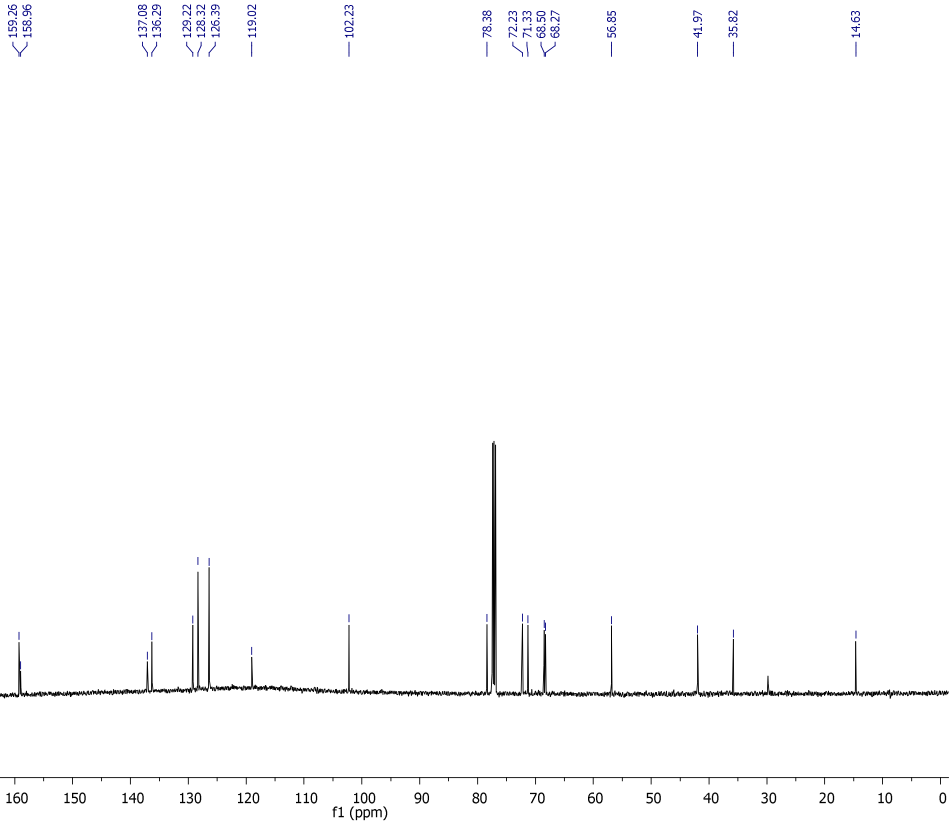

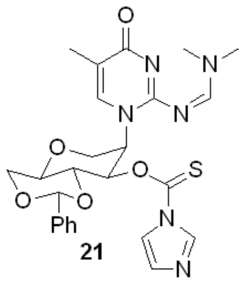

21

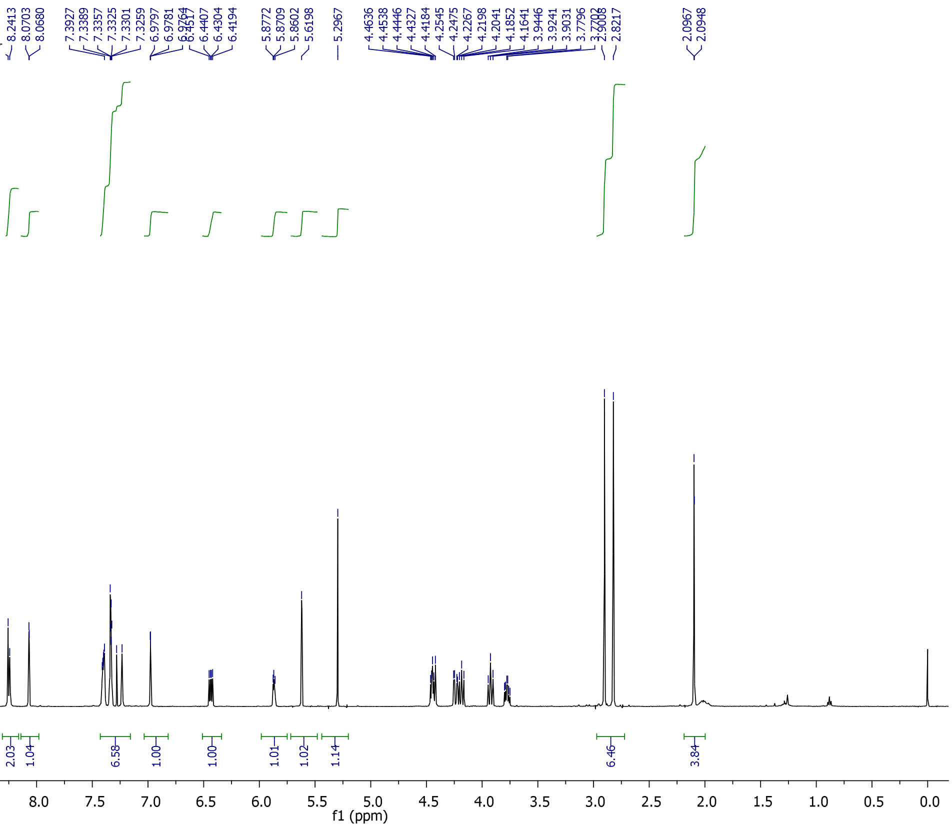

8.2413
8.0703
8.0680
7.3927
7.3389
7.3357
7.3325
7.3301
7.3259
6.9797
6.9781
6.4407
6.4304
6.4194
5.8772
5.8709
5.8602
5.6198
5.2967
4.4636
4.4538
4.4446
4.4327
4.4184
4.2545
4.2475
4.2267
4.2198
4.2041
4.1852
4.1641
3.9446
3.9241
3.9031
3.7796
2.8217
2.0967
2.0948
2.03
1.04
6.58
1.00
1.00
1.01
1.02
1.14
6.46
3.84
f1 (ppm)

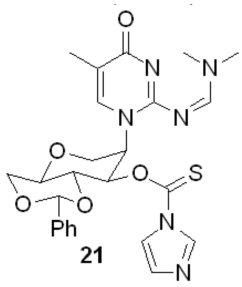

21

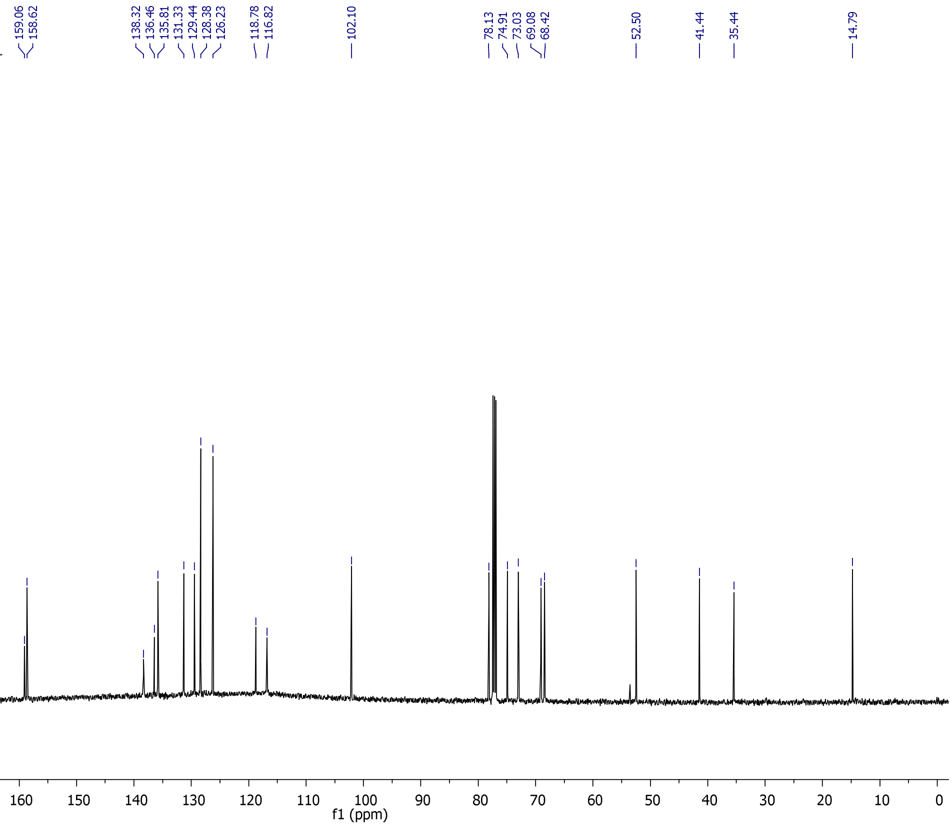

159.06
158.62
138.32
136.46
135.81
131.33
129.44
128.38
126.23
118.78
116.82
102.10
78.13
74.91
73.03
69.08
68.42
52.50
41.44
35.44
14.79
f1 (ppm)

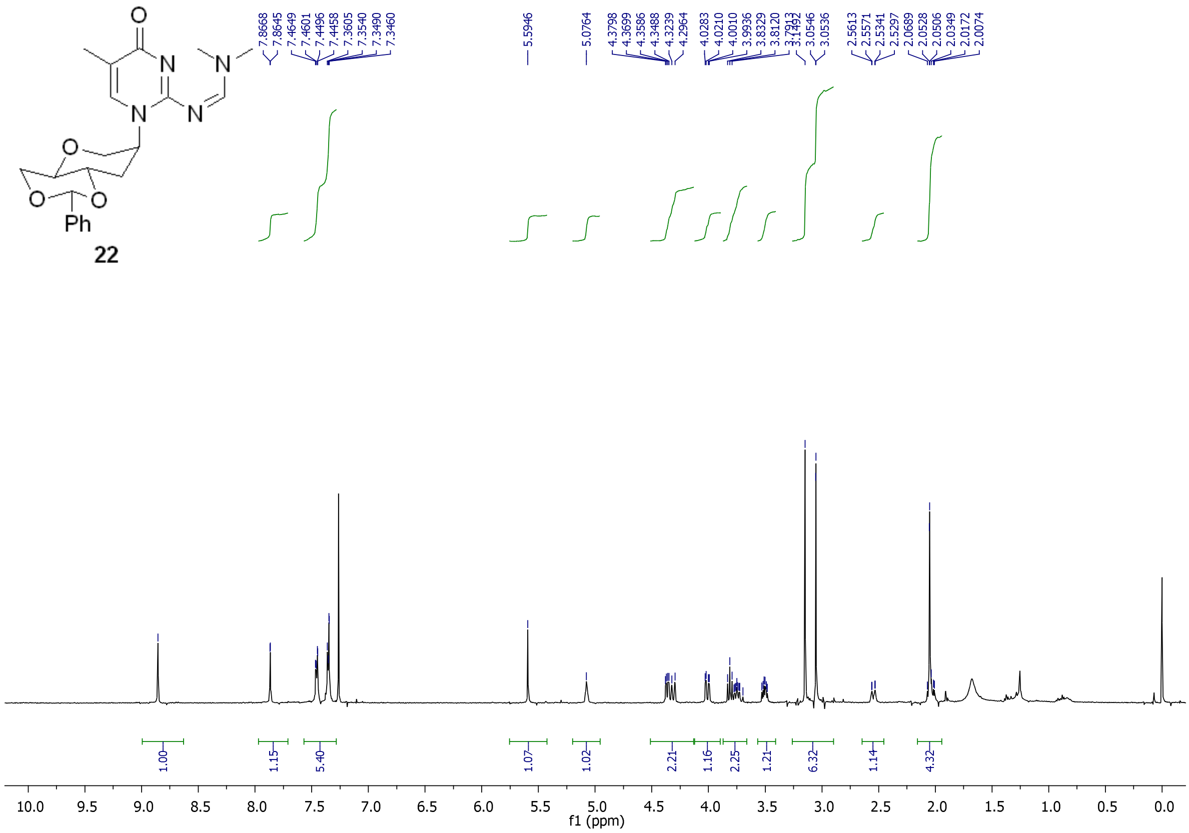
22
f1 (ppm)

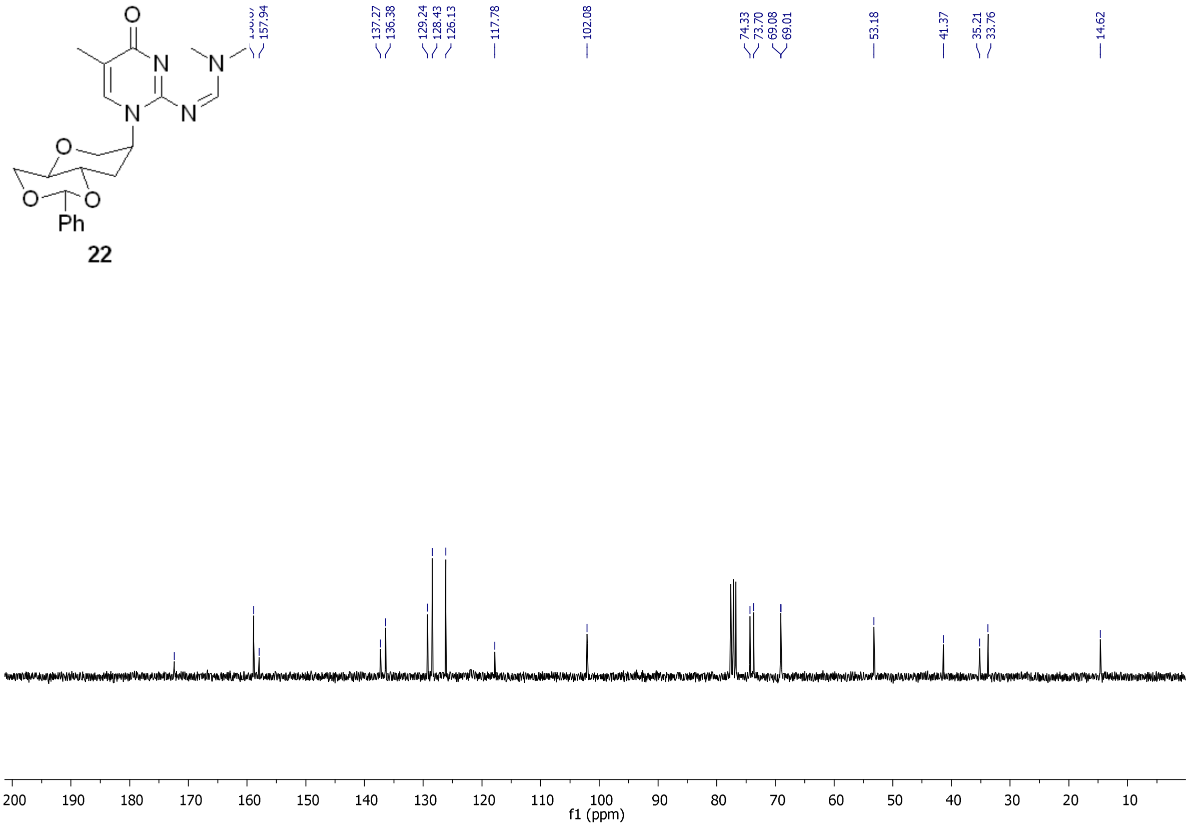
22
f1 (ppm)

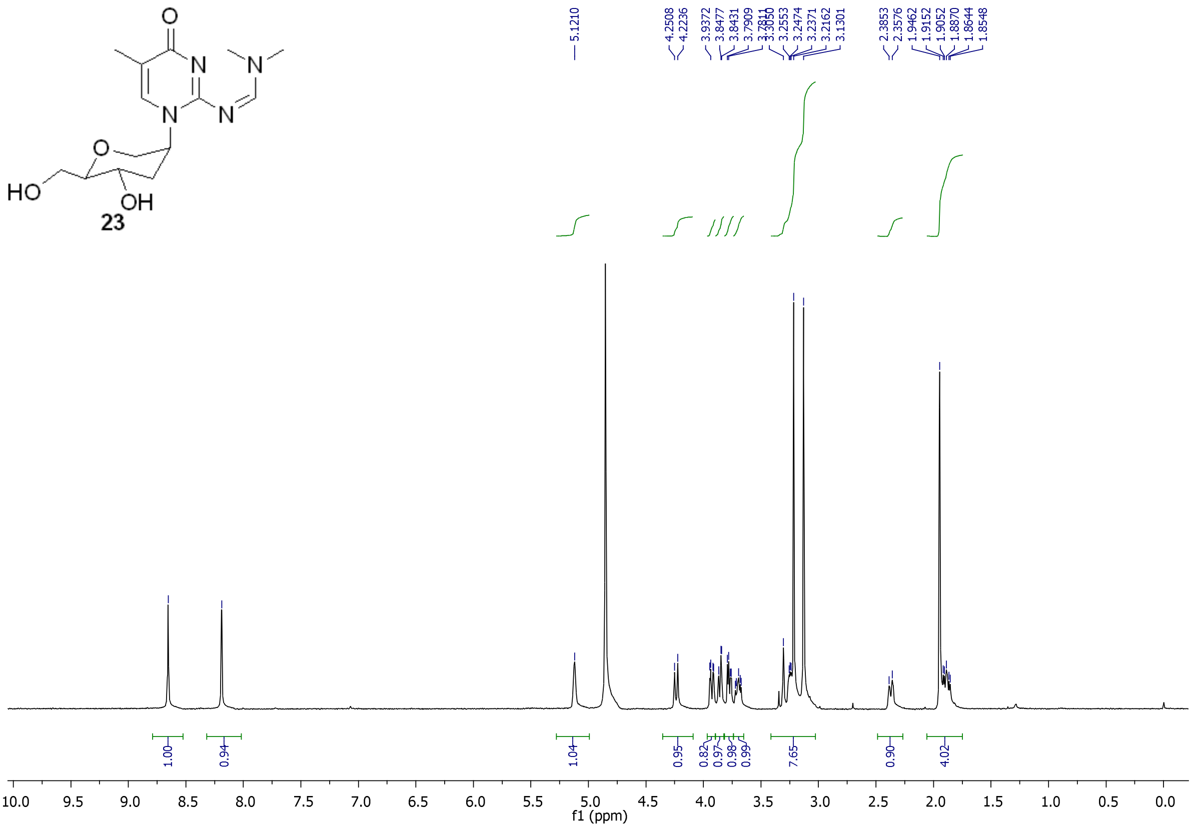
23
f1 (ppm)

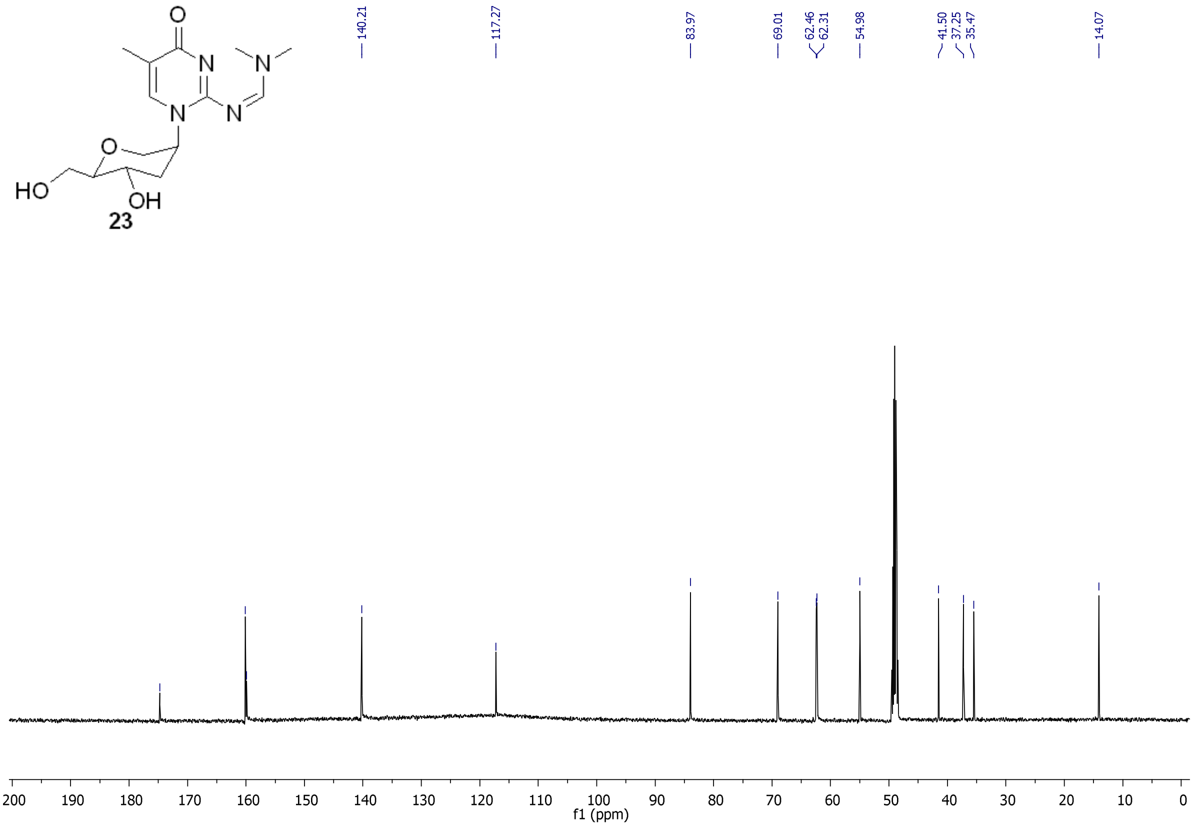
23
f1 (ppm)

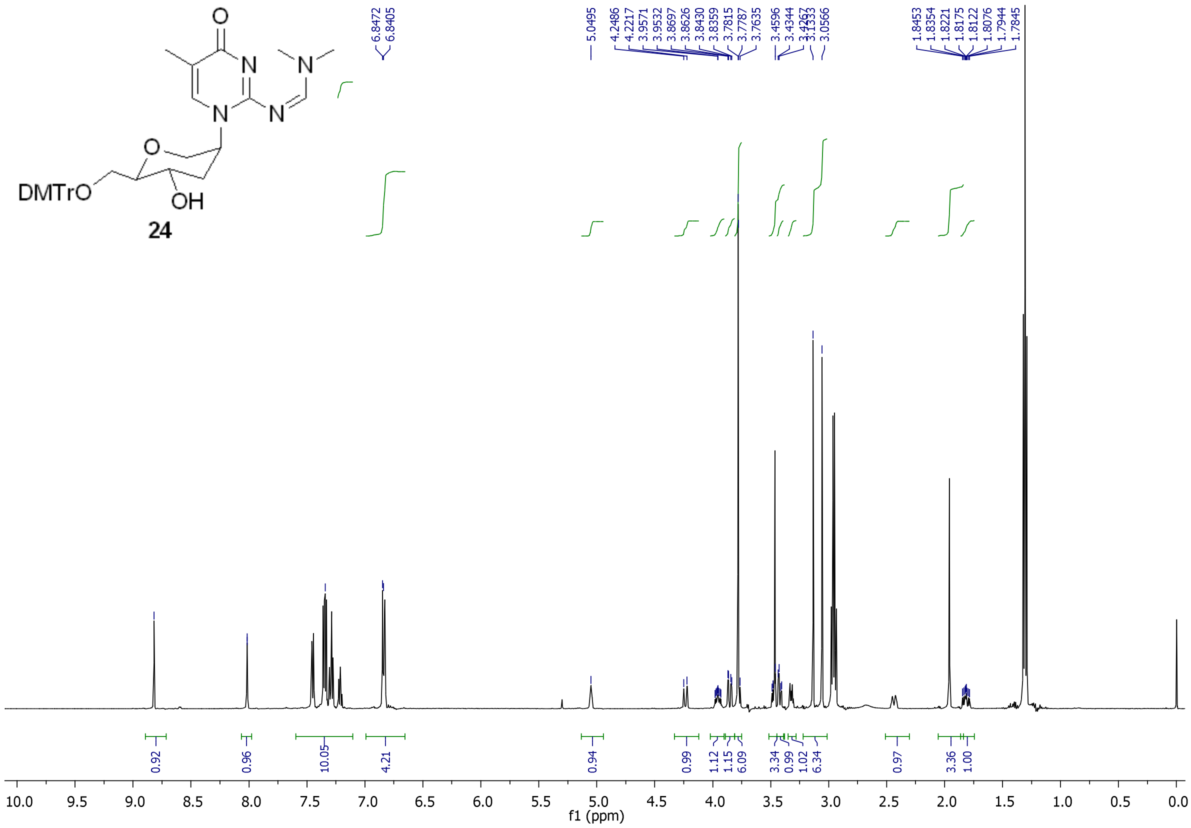
DMTrO
OH
24
6.8472
6.8405
5.0495
4.2486
4.2217
3.9571
3.9532
3.8697
3.8626
3.8430
3.8359
3.7815
3.7787
3.7635
3.4596
3.4344
3.0566
1.8453
1.8354
1.8221
1.8175
1.8122
1.8076
1.7944
1.7845
0.92
0.96
10.05
4.21
0.94
0.99
1.12
1.15
6.09
3.34
0.99
1.02
6.34
0.97
3.36
1.00
f1 (ppm)

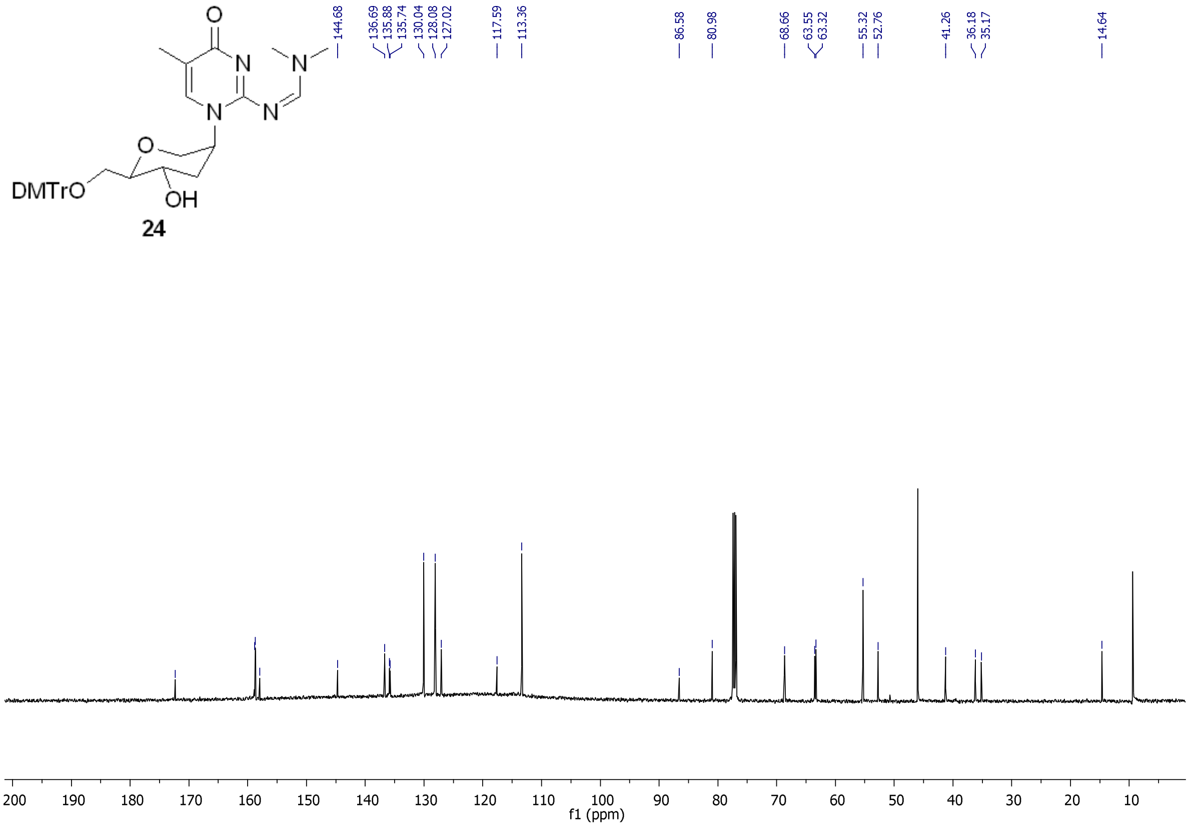
DMTrO
OH
24
144.68
136.69
135.88
135.74
130.04
128.08
127.02
117.59
113.36
86.58
80.98
68.66
63.55
63.32
55.32
52.76
41.26
36.18
35.17
14.64
f1 (ppm)

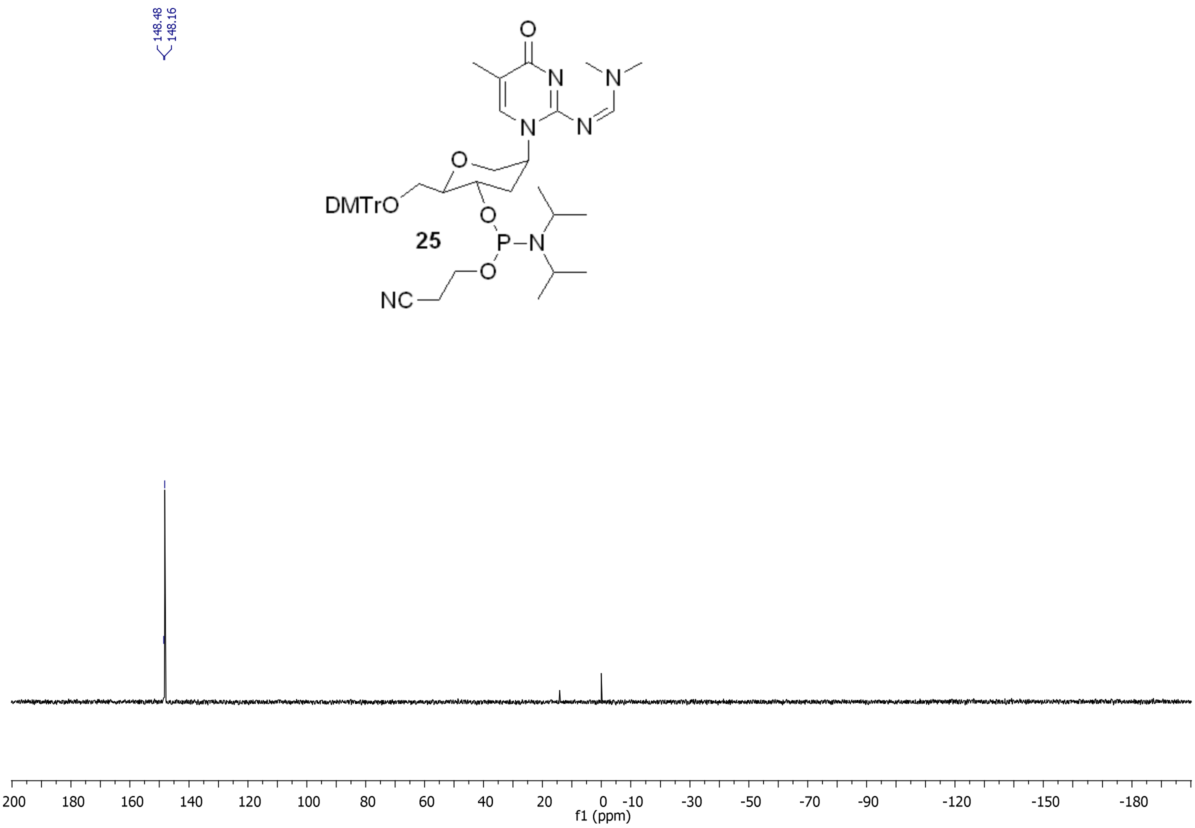
148.48
148.16
DMTrO
25
NC
200 180 160 140 120 100 80 60 40 20 0 -10 -30 -50 -70 -90 -120 -150 -180
f1 (ppm)

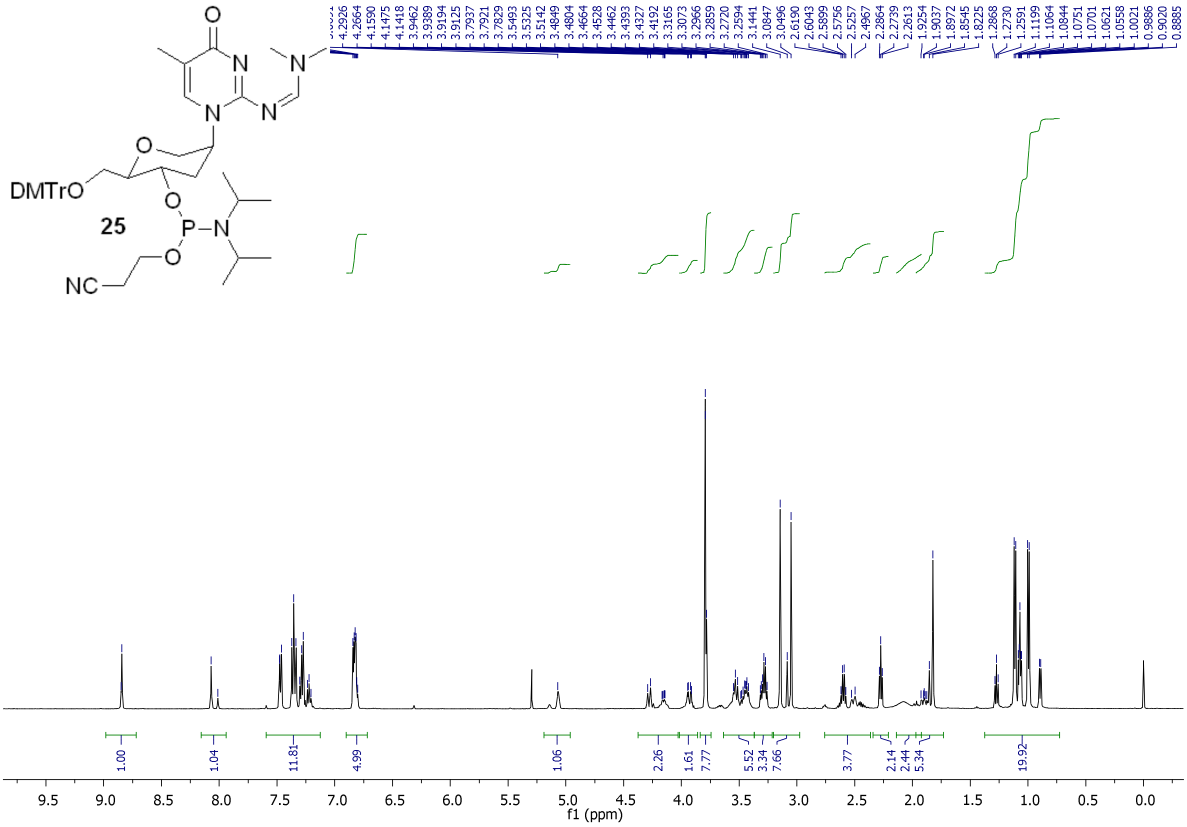
DMTrO
25
NC
4.2926 4.2664 4.1590 4.1475 4.1418 3.9462 3.9389 3.9194 3.9125 3.7937 3.7921 3.7829 3.5493 3.5325 3.5142 3.4849 3.4804 3.4664 3.4528 3.4462 3.4393 3.4327 3.4192 3.3165 3.3073 3.2966 3.2859 3.2720 3.2594 3.1441 3.0847 3.0496 2.6190 2.6043 2.5899 2.5756 2.5257 2.4967 2.2864 2.2739 2.2613 1.9254 1.9037 1.8972 1.8545 1.8225 1.2868 1.2730 1.2591 1.1199 1.1064 1.0844 1.0751 1.0701 1.0621 1.0558 1.0021 0.9886 0.9020 0.8885
1.00 1.04 11.81 4.99 1.06 2.26 1.61 7.77 5.52 3.34 7.66 3.77 2.14 2.44 5.34 19.92
9.5 9.0 8.5 8.0 7.5 7.0 6.5 6.0 5.5 5.0 4.5 4.0 3.5 3.0 2.5 2.0 1.5 1.0 0.5 0.0
f1 (ppm)

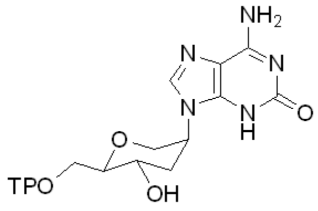

26

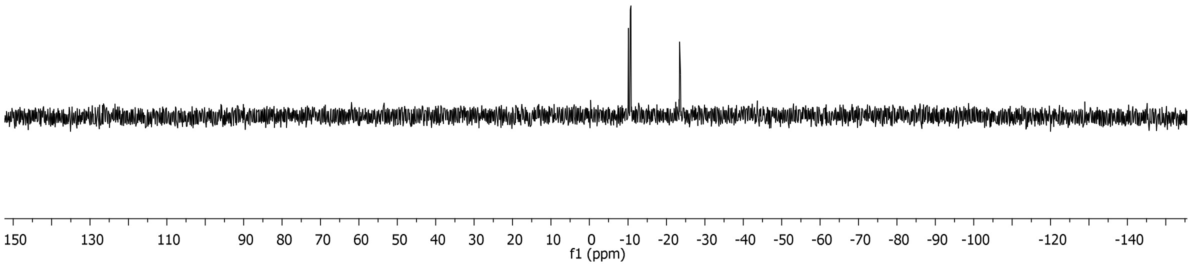

150 130 110 90 80 70 60 50 40 30 20 10 0 -10 -20 -30 -40 -50 -60 -70 -80 -90 -100 -120 -140
f1 (ppm)

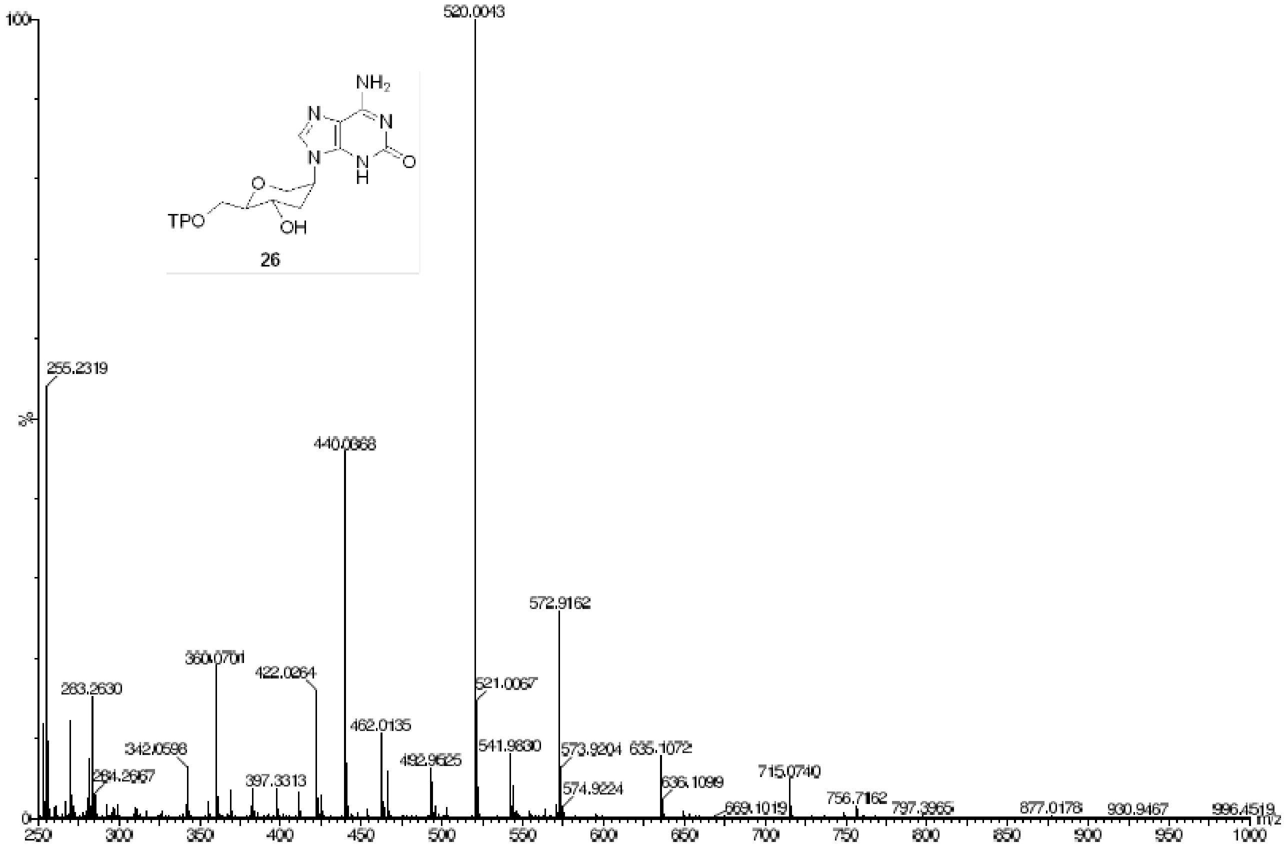

520.0043
255.2319
440.0368
572.9162
360.0701
422.0264
283.2630
521.0067
462.0135
342.0598
541.9630
492.9625
573.9204
635.1072
284.2667
397.3313
715.0740
636.1099
574.9224
756.7162
669.1019
797.3965
877.0178
930.9467
996.4519
26
TPO
OH
m/z
100
%
0
250 300 350 400 450 500 550 600 650 700 750 800 850 900 950 1000

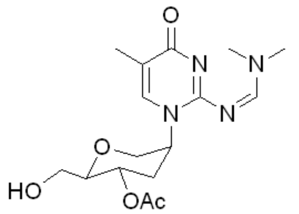

27

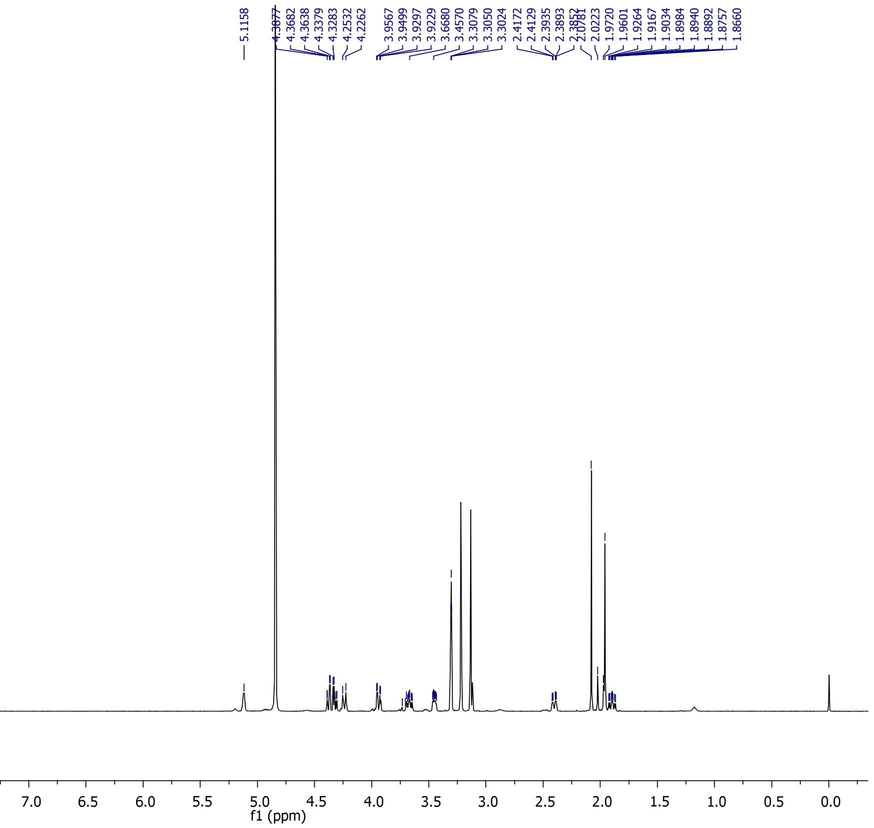

5.1158
4.3877
4.3682
4.3638
4.3379
4.3283
4.2532
4.2262
3.9567
3.9499
3.9297
3.9229
3.6680
3.4570
3.3079
3.3050
3.3024
2.4172
2.4129
2.3935
2.3893
2.3852
2.0781
2.0223
1.9720
1.9601
1.9264
1.9167
1.9034
1.8984
1.8940
1.8892
1.8757
1.8660
10.0 9.5 9.0 8.5 8.0 7.5 7.0 6.5 6.0 5.5 5.0 4.5 4.0 3.5 3.0 2.5 2.0 1.5 1.0 0.5 0.0
f1 (ppm)

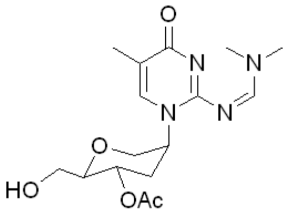

27

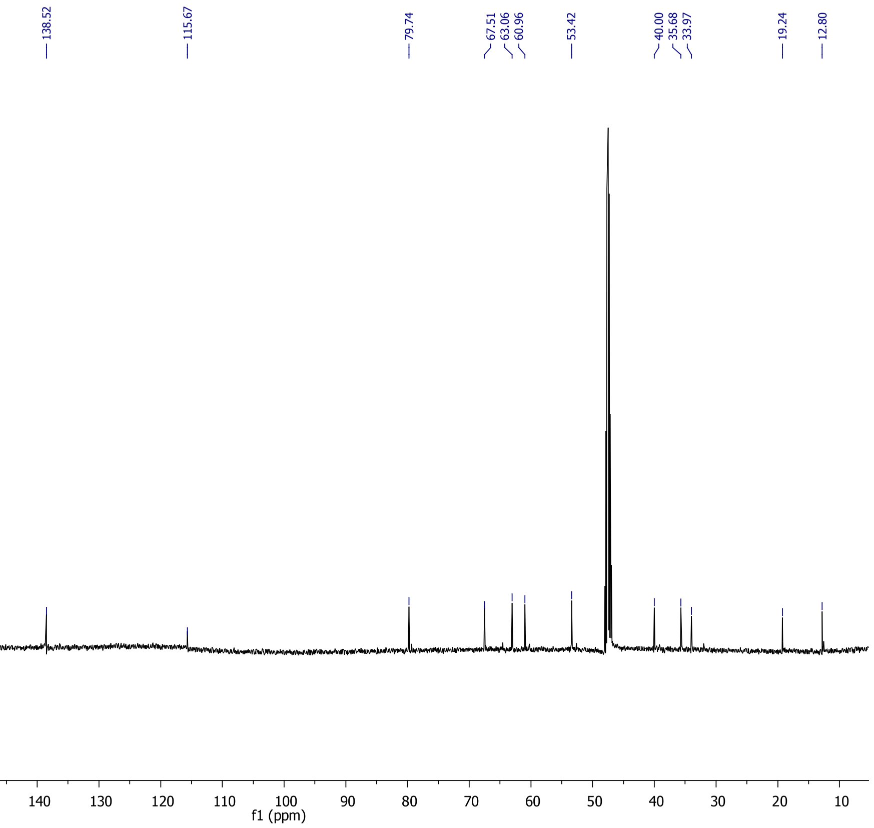

138.52
115.67
79.74
67.51
63.06
60.96
53.42
40.00
35.68
33.97
19.24
12.80
190 180 170 160 150 140 130 120 110 100 90 80 70 60 50 40 30 20 10
f1 (ppm)

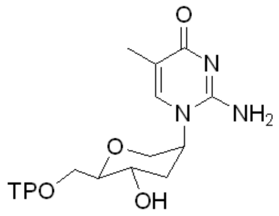
O
N
NH2
N
O
TPO
OH

28

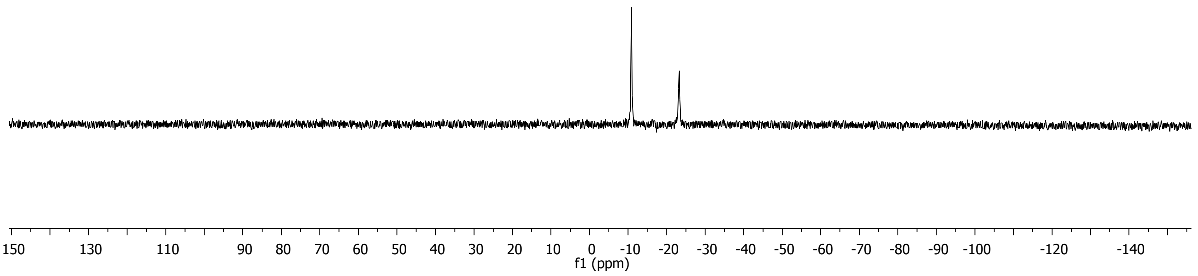
150 130 110 90 80 70 60 50 40 30 20 10 0 -10 -20 -30 -40 -50 -60 -70 -80 -90 -100 -120 -140
f1 (ppm)

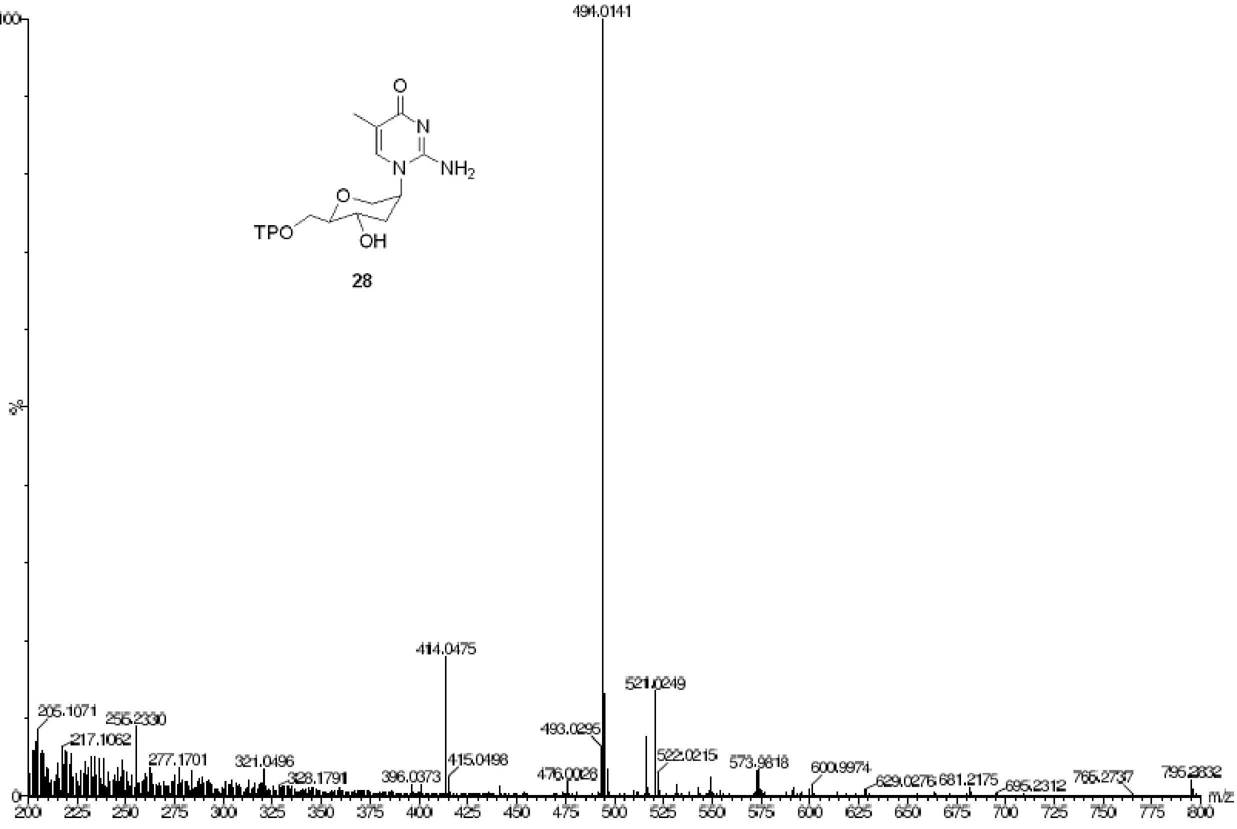
494.0141
100
%
0
414.0475
521.0249
205.1071
255.2330
217.1062
493.0295
277.1701
321.0496
328.1791
396.0373
415.0498
476.0028
522.0215
573.9818
600.9974
629.0276
681.2175
695.2312
765.2737
795.2832
m/z
200 225 250 275 300 325 350 375 400 425 450 475 500 525 550 575 600 625 650 675 700 725 750 775 800
28
